# A Bioinspired Catalytic Aerobic Oxidative C–H Functionalization of Primary Aliphatic Amines: Synthesis of 1,2-Disubstituted Benzimidazoles

**DOI:** 10.1002/chem.201502487

**Published:** 2015-07-23

**Authors:** Khac Minh Huy Nguyen, Martine Largeron

**Affiliations:** [a] UMR 8638 CNRS-Université Paris Descartes (Paris 5), Sorbonne Paris Cité, Faculté de Pharmacie de Paris 4 avenue de l'Observatoire, 75270 Paris cedex 06 (France) E-mail: martine.largeron@parisdescartes.fr

**Keywords:** aerobic oxidation, amines, benzimidazoles, C–H functionalization, homogeneous catalysis

## Abstract

Aerobic oxidative C–H functionalization of primary aliphatic amines has been accomplished with a biomimetic cooperative catalytic system to furnish 1,2-disubstituted benzimidazoles that play an important role as drug discovery targets. This one-pot atom-economical multistep process, which proceeds under mild conditions, with ambient air and equimolar amounts of each coupling partner, constitutes a convenient environmentally friendly strategy to functionalize non-activated aliphatic amines that remain challenging substrates for non-enzymatic catalytic aerobic systems.

Naturally occurring metalloenzymes constitute a rich source of inspiration for the design of synthetic catalysts because of their ability to perform controlled aerobic oxidations under very mild conditions.[1] Among metalloenzymes, copper amine oxidases (CuAOs) promote selective aerobic oxidation of primary amines through the cooperation of a quinone-based cofactor (topaquinone) and copper.[2] Recently, there has been a boost in the development of various catalytic methods for the aerobic oxidation of amines to imines,[3] owing to the importance of imines as pivotal intermediates in the synthesis of fine chemicals and pharmaceuticals. As a consequence, the use of CuAO-like catalytic systems has emerged in synthetic strategies,[4] especially because some biomimetic catalysts may offer advantages over CuAOs for expanding the scope of possible substrates. For example, a simple bioinspired *ortho*-quinone catalyst was reported for the oxidation of α-branched primary benzylic amines[5] and cooperative catalytic systems have been identified as effective mediators for the oxidation of secondary amines including nitrogen heterocycles such as indoline or tetrahydroquinoline,[6] two reaction types that natural CuAOs are not able to accomplish.

In contrast, the catalytic oxidation of non-activated primary alkylamines, which are natural substrates for CuAOs, has received little attention, probably because the generated alkylimines very easily isomerize into the unstable enamine tautomers. This is also the reason why alkylimines are difficult to prepare from the condensation of aliphatic aldehydes with amines. As a result, the catalytic oxidation of non-activated primary amines to imines remains a challenging task.

Recently, we reported a CuAO-like homogeneous co-catalytic system for the atom-economical oxidation of primary amines to imines under ambient air.[7] The catalytic process combines two redox couples in a way reminiscent of other biomimetic catalytic systems:[1b] the *o*-iminoquinone organocatalyst **1_ox_**, generated in situ from the corresponding *o*-aminophenol **1_red_**, is the substrate-selective catalyst, whereas the copper(II) salt serves as an electron transfer mediator. Interestingly, low loadings of biocompatible Cu^II^ and organocatalyst **1_ox_** are sufficient to activate the α-C–H bond of primary aliphatic amines, which are converted to cross-coupled imines through a transamination process that leads to the homocoupled imine intermediate, followed by dynamic transimination.[7b] The mild reaction conditions are highly favorable from a synthetic viewpoint, in particular for trapping the unstable alkylimines in situ for further reactions. We therefore envisioned the use of our bioinspired co-catalytic system in the one-pot synthesis of 1,2-disubstituted benzimidazoles. These compounds are important targets in drug discovery, as shown by the profusion in the market of pharmaceutical products such as telmisartan and candesartan as antihypertensives, or astemizole, clemizole, and bilastine as antihistaminic agents.[8]

Over the past few years, novel methodologies involving transition-metal-catalyzed C–H functionalization reactions[9] have been directed toward the regiocontrolled synthesis of 1,2-disubstituted benzimidazoles.[10] Meanwhile, aerobic catalytic oxidative cross-coupling reactions employing either alcohols or amines as substrates have also been reported for the synthesis of benzazoles.[11] However, several of these methods suffer drawbacks such as elevated temperatures (90–160 °C) and/or oxygen pressure. In particular, non-activated primary alcohols or amines are generally ineffective starting materials for this transformation. In addition, metal-free oxidative cross-coupling reactions of primary alcohols or amines with *o*-amino anilines have been described, but the scope is limited to benzylic-type substrates.[12]

Herein, we describe a bioinspired catalytic oxidative coupling of a diverse range of activated and non-activated primary amines with *o*-amino anilines under ambient air that leads to 1,2-disubstituted benzimidazoles through multistep oxidation and nucleophilic addition reactions (Scheme 1).

**Scheme 1:**
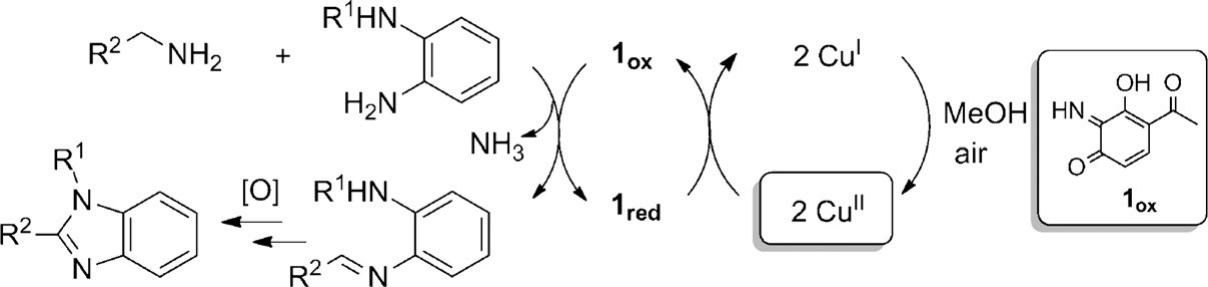
Cross-coupling of primary amines to give 1,2-disubstituted benzimidazoles by using a bioinspired co-catalytic system.

As a starting point, we examined the aerobic oxidative cross-coupling of benzylamine **2 a** with *o*-aminoaniline **3 a** under our previously optimized ambient conditions for the preparation of cross-coupled imines,[7b] but no significant amounts of 1,2-benzimidazole **4 a** were isolated after 24 h (Table 1, entry 1). Significant improvements were achieved by heating the coupling-reaction mixture at 45 °C and by using CuBr_2_ as the copper catalyst,[11c] which afforded 1,2-benzimidazole **4 a** in 82 % yield of the isolated product (Table 1, entry 5). When the reaction was conducted at higher temperature (60 °C), the yield of **4 a** decreased to 75 % due to the slow decomposition of the *o*-iminoquinone organocatalyst **1_ox_** to melanin-like polymers (Table 1, entry 6).[13] As expected,[7b] further variation of the solvent led only to a decrease in the yield (Table 1, entries 7–9). Control studies revealed that the synthesis of **4 a** could also be performed in the absence of copper catalyst, albeit with a markedly reduced yield (30 %), owing to the slow spontaneous oxidation of **1_red_** to organocatalyst **1_ox_**, whereas no reaction occurred at 45 °C when CuBr_2_ was used as the sole catalyst. These results confirmed the cooperative action of organocatalyst **1_ox_** as the substrate-selective catalyst and copper salt as the electron transfer mediator to facilitate the aerobic oxidation of amines to imines.[7]

**Table 1:** Optimization of the Cu^II^/1_ox_-catalyzed aerobic oxidative cross-coupling of benzylamine 2 a with *o*-aminoaniline 3 a^[a]^

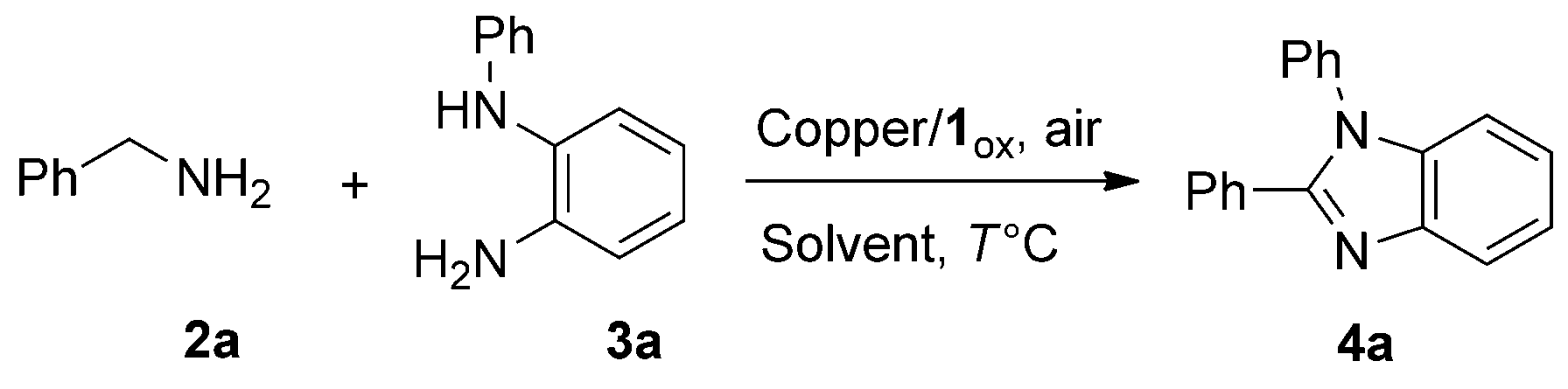				
Entry	Copper catalyst	Solvent	Temperature [°C]	Yield [%]^[b]^
1	Cu(OAc)_2_	MeOH	25	5
2	Cu(OAc)_2_	MeOH	40	48
3	CuCN	MeOH	40	40
4	CuBr_2_	MeOH	40	70
5	CuBr_2_	MeOH	45	82
6	CuBr_2_	MeOH	60	75
7	CuBr_2_	EtOH	45	68
8	CuBr_2_	MeCN	45	45
9	CuBr_2_	THF	45	10

[a] The reactions were carried out using equimolar amounts of benzylamine **2 a** and *o*-aminoaniline **3 a** on a 1.25 mmol scale, in the presence of 4 mol % of **1_red_** and 0.4 mol % of copper salt, in 25 mL of solvent, under ambient air at the indicated temperature for 24 h. After 6 h, an additional aliquot of **1_red_** (2 mol %) was added; [b] yield of isolated product.

With the optimized reaction conditions in hand (Table 1, entry 5), we examined the reactions of a series of primary amine substrates. Variously substituted benzylamines afforded the corresponding 1,2-benzimidazoles in 74–82 % yield regardless of the electronic character of the substituents (Table 2, entries 1–8). In contrast, the rate of the reaction was affected by steric effects[11b, 14] as shown by benzylic amine **2 i** which gave benzimidazole **4 i** in 75 % yield after 48 h (Table 2, entry 9). 1-Naphthylmethylamine **2 j** could also be used as the amine substrate leading to 1,2-benzimidazole **4 j** in 71 % yield (Table 2, entry 10). Likewise, heterocyclic compounds such as 2-thiophenemethylamine **2 k** and furfurylamine **2 l** could be converted into the corresponding 1,2-benzimidazoles **4 k** and **4 l** in good yields with a prolonged reaction time (Table 2, entries 11 and 12).

**Table 2:** CuBr_2_/1_ox_-catalyzed aerobic oxidative cross-coupling of a range of primary activated and non-activated amines 2 with *o*-aminoaniline 3 a^[a]^

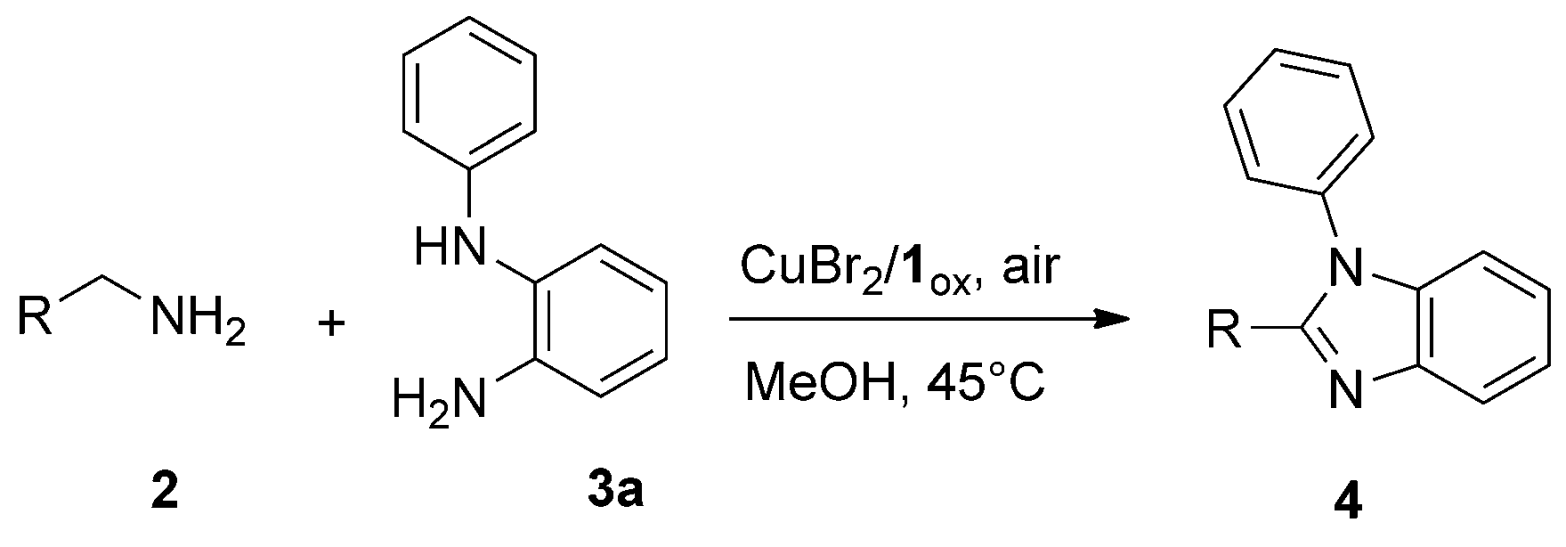					
Entry	Amine Substrate2		Benzimidazole Product4		Yield [%]^[c]^
1	**2 a**	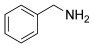	**4 a**	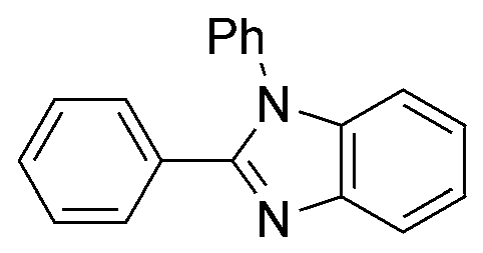	82
2	**2 b**	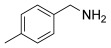	**4 b**	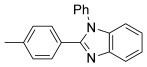	80
3	**2 c**	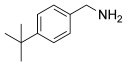	**4 c**	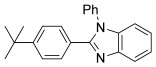	78
4	**2 d**	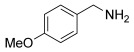	**4 d**	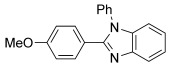	80
5	**2 e**	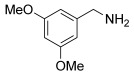	**4 e**	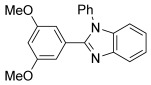	74^[d]^
6	**2 f**	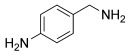	**4 f**	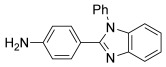	75
7	**2 g**	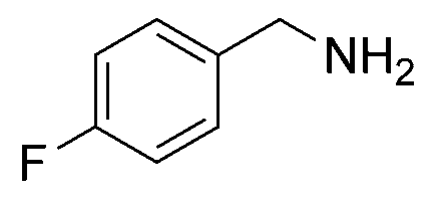	**4 g**	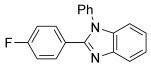	80
8	**2 h**	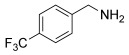	**4 h**	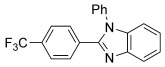	74
9	**2 i**	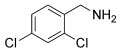	**4 i**	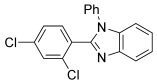	75^[d]^
10	**2 j**	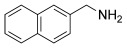	**4 j**	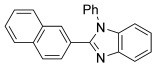	71
11	**2 k**	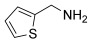	**4 k**	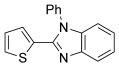	81^[d]^
12	**2 l**	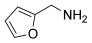	**4 l**	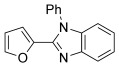	70^[d]^
13^[b]^	**2 m**	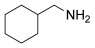	**4 m**	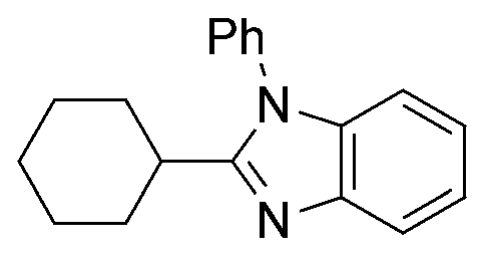	58
14^[b]^	**2 n**	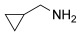	**4 n**	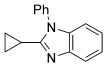	68^[e]^
15^[b]^	**2 o**	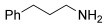	**4 o**	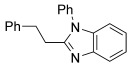	55
16^[b]^	**2 p**	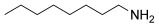	**4 p**	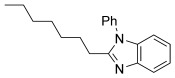	61
17^[b]^	**2 q**	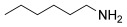	**4 q**	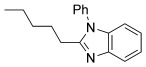	58
18^[b]^	**2 r**	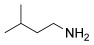	**4 r**	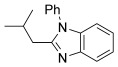	53
19^[b]^	**2 s**	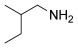	**4 s**	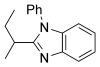	51^[d]^

[a] The reactions were carried out using equimolar amounts of primary amines **2** and *o*-aminoaniline **3 a** on a 1.25 mmol scale, in the presence of 4 mol % of **1_red_** and 0.4 mol % of CuBr_2_, in 25 mL of MeOH, under ambient air for 24 h. After 6 h, an additional aliquot of **1_red_** (2 mol %) was added; [b] *T*=60 °C; [c] yield of isolated product; [d] yield after 48 h; [e] as volatile alkylamine **2 n** was lost at 60 °C, an additional 0.5 equivalent of alkylamine was added after 6 h.

Our optimized conditions were further applied to the oxidation of non-activated primary aliphatic amines (Table 2, entries 13–19). Then, the aerobic oxidative cross-coupling of aminomethylcyclohexane **2 m** with *o*-aminoaniline **3 a** afforded 1,2-benzimidazole **4 m** in only 36 % yield after 24 h. The lower yield could be due to the instability of the generated alkylimine intermediate which readily isomerizes into the enamine tautomer.[7b, 11b, 14] However, the yield of 1,2-benzimidazole **4 m** could be improved to 58 % at a slightly elevated reaction temperature of 60 °C (Table 2, entry 13). Other aliphatic amines were also oxidized to the corresponding benzimidazoles in moderate to good yields (Table 2, entries 14–19). As previously reported for catalyzed aerobic oxidation of non-activated alcohols,[15] we observed a decreased reactivity for β-branched alkylamines, but some activity could be regained by increasing the reaction time (Table 2, entry 19).

In a final series of experiments, we carried out reactions using two commercially available *o*-aminoanilines as in situ imine traps. *N*-methyl-1,2-phenylenediamine (Table 3, entries 1 and 3) gave similar results to its *N*-phenyl congener (Table 2, entries 1 and 14), whereas *N*-(4-chlorophenyl)-1,2-phenylenediamine delivered the desired products with acceptable yields only after 48 h (Table 3, entries 2 and 4).

**Table 3:** CuBr_2_/1_ox_-catalyzed aerobic oxidative cross-coupling of benzylamine 2 a or aminomethylcyclopropane 2 n with *o*-aminoanilines 3^[a]^

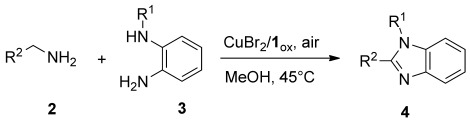				
Entry	R^2^	R^1^	Benzimidazole Product4	Yield [%]^[c]^
1	phenyl	methyl	**4 t**	81
2	phenyl	*p*-chlorophenyl	**4 u**	84^[d]^
3^[b]^	cyclopropyl	methyl	**4 v**	73^[e]^
4^[b]^	cyclopropyl	*p*-chlorophenyl	**4 w**	72^[d,e]^

[a] The reactions were carried out using equimolar amounts of primary amine **2 a** (or **2 n**) and *o*-aminoaniline **3** on a 1.25 mmol scale, in the presence of 4 mol % of **1_red_** and 0.4 mol % of CuBr_2_, in 25 mL of MeOH, under ambient air for 24 h. After 6 h, an additional aliquot of **1_red_** (2 mol %) was added; [b] *T*=60 °C; [c] yield of isolated product; [d] yield after 48 h; [e] as volatile alkylamine **2 n** was lost at 60 °C, an additional 0.5 equivalent of alkylamine was added after 6 h (entries 3 and 4) and after 24 h (entry 4).

A proposed reaction pathway is shown in Scheme 2. The initial step is the formation of the cross-coupled imine through a transamination process that leads to the homocoupled imine intermediate followed by dynamic transimination.[7b] The intramolecular addition of the amine group affords the cyclic intermediate **A**, which is further oxidized to 1,2-disubstituted benzimidazole **4**.

**Scheme 2:**
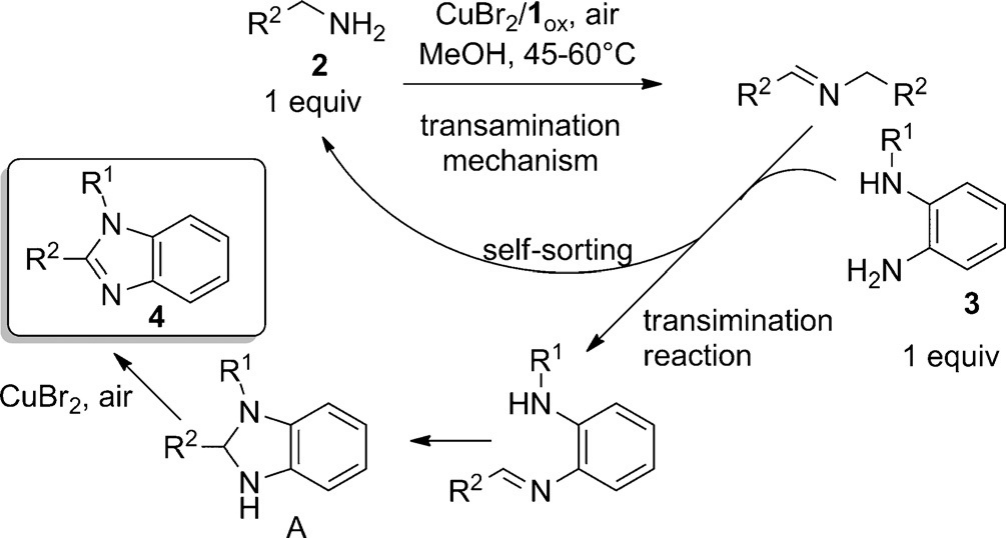
Proposed mechanism for the CuBr_2_/1_ox_-mediated C–H functionalization of primary aliphatic amines giving rise to 1,2-disubstituted benzimidazoles.

We have validated our cascade reaction sequence with two control experiments (Scheme 3). Firstly, *N*-benzylidenebenzylamine was reacted with two equivalents of *o*-amino aniline **3 a** under the standard conditions (Table 1, entry 5). Then, 1,2-disubstituted benzimidazole **4 a** was isolated in 82 % yield, in agreement with *N*-benzylidenebenzylamine as the first imine intermediate. Secondly, when the same reaction was realized in the absence of CuBr_2_ catalyst, only 60 % yield of benzimidazole **4 a** was isolated. This result indicated that CuBr_2_ also mediated the ultimate oxidation step that led to the benzimidazole product.[11b,c]

**Scheme 3:**
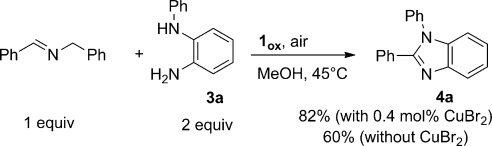
Control experiments starting from *N*-benzylidenebenzylamine.

In conclusion, we have developed a bioinspired catalytic aerobic oxidative C–H functionalization of primary amines that leads to 1,2-disubstituted benzimidazoles under environmentally benign reaction conditions. Notably, low loadings of biocompatible CuBr_2_ electron transfer mediator and topaquinone-like organocatalyst are sufficient to activate, under ambient air, the α-C–H bond of non-activated primary amines that are known to be challenging substrates for non-enzymatic catalytic aerobic systems. This biomimetic approach should be useful for other synthetic applications involving unstable alkylimines as the intermediate.

## Experimental Section

**General experimental procedure for the synthesis of 1,2-disubstituted benzimidazoles**: Equimolar amounts of benzylamine **2** (1.25 mmol) and *o*-amino aniline **3** (1.25 mmol) with reduced organocatalyst **1_red_**[16] (0.05 mmol, 4 mol %) and copper(II) bromide (0.005 mmol, 0.4 mol %) were mixed in methanol (25 mL) in an air atmosphere. The reaction mixture was stirred at 45 °C for 6 h. Then, an additional aliquot of **1_red_** (0.025 mmol, 2 mol %) was introduced into the reaction mixture and the reaction was continued for 18 h. The solvent was then removed by evaporation under reduced pressure and the residue was purified by column chromatography on silica gel (eluent: dichloromethane/methanol 99:1 *v*/*v*) to afford the desired benzimidazole **4** (see the Supporting Information).

The above procedure is generally representative for all of the products shown in Tables 2 and 3. Any deviations from this protocol are specified in the footnotes of the tables.

## Supporting Information

As a service to our authors and readers, this journal provides supporting information supplied by the authors. Such materials are peer reviewed and may be re-organized for online delivery, but are not copy-edited or typeset. Technical support issues arising from supporting information (other than missing files) should be addressed to the authors.

miscellaneous_information
