# Supplementary material for: A Bioinspired Catalytic Aerobic Oxidative C–H Functionalization of Primary Aliphatic Amines: Synthesis of 1,2-Disubstituted Benzimidazoles
Source: Chemistry. 2015 Jul 23;21(36):12606–10. doi: 10.1002/chem.201502487 (PMC4557039; doi:10.1002/chem.201502487)

CHEMISTRY
A European Journal

Supporting Information

A Bioinspired Catalytic Aerobic Oxidative C−H Functionalization of Primary Aliphatic Amines: Synthesis of 1,2-Disubstituted Benzimidazoles

Khac Minh Huy Nguyen and Martine Largeron*[a]

chem_201502487_sm_miscellaneous_information.pdf

Supporting Information

Table of Contents

I. General considerations

II. Analytical data for known compounds whose NMR data are not reported

III. References

IV. Analytical data for new compounds

V. High field ^{1}H NMR spectra and 1D proton-decoupled ^{13}C NMR spectra for all compounds **4a-w**

I. General considerations

^{1}H NMR and 1D proton decoupled ^{13}C NMR spectra were recorded in $CDCl_3$ on a Brucker AC-300 spectrometer operating at 300 MHz and 75 MHz, respectively. Chemical shifts δ are given in ppm relative to TMS and coupling constants *J*, in Hertz. The measurements were carried out using the standard pulse-sequences. The carbon type (methyl, methylene, methine, or quaternary) was determined by DEPT experiments. High-resolution mass spectra (HRMS) were performed on a Bruker maXis mass spectrometer operating in the positive ion mode. Chemicals were commercial products of the highest available purity and were used as supplied.

The following compounds are known with analytical data reported in the literature: benzimidazole **4a**[1-3] ; benzimidazole **4b**[2] ; benzimidazole **4d**[2] ; benzimidazole **4g**[2]; benzimidazole **4h**[2]; benzimidazole **4k**[3]; benzimidazole **4q**[4]; benzimidazole **4s**[5]; benzimidazole **4t**[6]; benzimidazole **4u**[3]; benzimidazole **4v**[7]

II. Analytical data for known compounds for which NMR data are not reported

Benzimidazole 4c.[8] ^{1}H NMR (300 MHz, $CDCl_3$, 25°C, TMS): δ = 1.35 (s, 9H, CH_3), 7.28 (m, 2H, Ar), 7.35-7.40 (m, 5H, Ar), 7.55-7.58 (m, 5H, Ar), 7.95 (d, *J* = 8 Hz, 1H, Ar); ^{13}C NMR (75 MHz, $CDCl_3$, 25°C, TMS): δ = 31.2 (3 x CH_3), 34.8 (Cq), 110.4 (CH, Ar), 119.7 (CH, Ar), 122.9 (CH, Ar), 123.2 (CH, Ar), 125.3 (2 x CH, Ar), 126.9 (Cq), 127.6 (2 x CH, Ar), 128.6 (CH, Ar), 129.1 (2 x CH, Ar), 129.9 (2 x CH, Ar), 137.2 (Cq), 137.3 (Cq), 143.0 (Cq), 152.4 (Cq), 152.7 (Cq); HRMS (ESI+) *m/z* calcd for $C_{23}H_{22}N_2$ $[M+H]^+$ 327.1856, Found 327.1856.

Benzimidazole 4e.[9] ^{1}H NMR (300 MHz, $CDCl_3$, 25°C, TMS): δ = 3.66 (s, 6H, 2 x CH_3), 6.49 (m, 1H, Ar), 6.78 (m, 2H, Ar), 7.26-7.28 (m, 2H, Ar), 7.33-7.36 (m, 3H, Ar), 7.50-7.53 (m, 3H, Ar), 7.95 (d, *J* = 8 Hz, 1H, Ar) ; ^{13}C NMR (75 MHz, $CDCl_3$, 25°C, TMS): δ = 55.3 (2 x CH_3), 102.7 (CH, Ar), 107.2 (2 x CH, Ar), 110.5 (CH, Ar), 119.9 (CH, Ar), 123.1 (CH, Ar), 123.5 (CH, Ar), 127.5 (2 x CH, Ar), 128.6 (CH, Ar), 129.9 (2 x CH, Ar), 131.5, (Cq), 137.1 (Cq), 137.3 (Cq), 142.8 (Cq), 152.1 (Cq), 160.5 (2 x Cq); HRMS (ESI+) *m/z* calcd for $C_{21}H_{18}N_2O_2$ $[M+H]^+$ 331.1441, Found 331.1441.

Benzimidazole 4f.[10] ^{1}H NMR (300 MHz, $CDCl_3$, 25°C, TMS): δ = 3.65 (m, 2H, NH, D_2O exchanged), 6.60 (d, *J* = 8.55 Hz, 2H, Ar), 7.25-7.43 (m, 7H, Ar), 7.53-7.56 (m, 3H, Ar), 7.90 (d, *J* = 7.9 Hz, 1H, Ar) ; ^{13}C NMR (75 MHz, $CDCl_3$, 25°C, TMS): δ = 110.5 (CH, Ar), 115.4 (CH, Ar), 115.7 (CH, Ar), 119.8 (CH, Ar), 123.2 (CH, Ar), 123.5 (CH, Ar), 126. 09 (CH, Ar), 126.13 (CH, Ar), 127.4 (CH, Ar), 128.8 (CH, Ar), 130.0 (CH, Ar), 131.4 (CH, Ar), 131.5 (CH, Ar), 136.8 (Cq), 137.2 (Cq), 142.8 (Cq), 151.4

(Cq), 161.8 (Cq), 165.1 (Cq); HRMS (ESI+) *m/z* calcd for $C_{19}H_{15}N_3$ $[M+H]^+$ 286.1344, Found 286.1347.

Benzimidazole 4j.[9] ^1H NMR (300 MHz, $CDCl_3$, 25°C, TMS): δ = 7.23-7.55 (m, 12H, Ar), 7.89-7.94 (m, 2H, Ar), 8.05 (d, *J* = 8.7 Hz, 1H, Ar), 8.18 (m, 1H, Ar) ; ^{13}C NMR (75 MHz, $CDCl_3$, 25°C, TMS): δ =110.7 (CH, Ar), 120.2 (CH, Ar), 123.1 (CH, Ar), 123.5 (CH, Ar), 124.6 (CH, Ar), 125.9 (CH, Ar), 126.3 (CH, Ar), 126.5 (2 x CH, Ar), 126.7 (CH, Ar), 127.7 (Cq), 128.1 (CH, Ar), 128.3 (CH, Ar), 129.5 (3 x CH, Ar), 130.0 (CH, Ar), 132.2 (Cq), 133.6 (Cq), 136.1 (Cq), 136.4 (Cq), 143.2 (Cq), 151.9 (Cq); HRMS (ESI+) *m/z* calcd for $C_{23}H_{16}N_2$ $[M+H]^+$ 321.1386, Found 321.1387.

Benzimidazole 4l.[11] ^1H NMR (300 MHz, $CDCl_3$, 25°C, TMS): δ = 6.21 (d, *J* = 3.4 Hz, 1H, furyl), 6.4 (m, 1H, furyl), 7.13 (d, *J* = 7.8 Hz, 1H, furyl), 7.25-7.40 (m, 2H, Ar), 7.44-7.47 (m, 2H, Ar), 7.52-7.53 (m, 1H, Ar), 7.62-7.63 (m, 3H, Ar), 7.92 (d, *J* = 8.0 Hz, 1H, Ar) ; ^{13}C NMR (75 MHz, $CDCl_3$, 25°C, TMS): δ = 110.2 (CH, Ar), 111.5 (CH, Ar), 112.2 (CH, Ar), 119.9 (CH, Ar), 123.1 (CH, Ar), 123.6 (CH, Ar), 128.0 (2 x CH, Ar), 129.5 (CH, Ar), 130.0 (2 x CH, Ar), 136.4 (Cq), 137.2 (Cq), 142.9 (Cq), 144.2 (2 x CH, Ar), 144.5 (Cq); HRMS (ESI+) *m/z* calcd for $C_{17}H_{12}N_2O$ $[M+H]^+$ 261.1028, Found 261.1035.

Benzimidazole 4m.[12] ^1H NMR (300 MHz, $CDCl_3$, 25°C, TMS): δ = 1.22-1.97 (m, 10H, cyclohexyl), 2.73-2.78 (m, 1H, cyclohexyl), 7.10 (d, *J* = 8 Hz, 1H, Ar), 7.22 (t, *J* = 8 Hz, 1H, Ar), 7.31 (d, *J* = 8 Hz, 1H, CH, Ar), 7.40 (m, 2H, Ar), 7.61-7.64 (m, 3H, Ar), 7.86 (d, *J* = 7.9 Hz, 1H, Ar) ; ^{13}C NMR (75 MHz, $CDCl_3$, 25°C, TMS): δ = 25.7 (CH_2), 26.2 (2 x CH_2), 32.0 (2 x CH_2), 36.2 (CH), 110.1 (CH, Ar), 119.1 (CH, Ar), 122.4 (CH, Ar), 122.5 (CH, Ar), 127.6 (2 x CH, Ar), 129.0 (CH, Ar), 130.0 (2 x CH, Ar), 136.0 (Cq), 136.4 (Cq), 142.3 (Cq), 159.3 (Cq); HRMS (ESI+) *m/z* calcd for $C_{19}H_{20}N_2$ $[M+H]^+$ 277.1705, Found 277.1693.

Benzimidazole 4n.[13] ^1H NMR (300 MHz, $CDCl_3$, 25°C, TMS): δ = 1.05-1.10 (m, 2H, cyclopropyl), 1.37-1.40 (m, 2H, cyclopropyl), 1.90 (m, 1H, cyclopropyl), 7.18-7.20 (m, 2H, Ar), 7.25-7.29 (m, 1H, Ar), 7.50-7.54 (m, 3H, Ar), 7.60-7.62 (m, 2H, Ar), 7.77 (d, *J* = 7.9 Hz, 1H, Ar) ; ^{13}C NMR (75 MHz, $CDCl_3$, 25°C, TMS): δ = 8.3 (CH), 9.6 (2 x CH_2), 109.7 (CH, Ar), 118.8 (CH, Ar), 122.2 (CH, Ar), 122.4 (CH, Ar), 127.4 (2 x CH, Ar), 128.7 (CH, Ar), 129.9 (2 x CH, Ar), 136.0 (Cq), 136.5 (Cq), 142.4 (Cq), 156.9 (Cq); HRMS (ESI+) *m/z* calcd for $C_{16}H_{14}N_2$ $[M+H]^+$ 235.1235, Found 235.1228.

III. References

[1] H. Baars, A. Beyer, S. V. Kohlhepp, C. Bolm, *Org. Lett.* **2014**, *16*, 536-539.
[2] J. Jayabharathi, V. Thanikachalam, K. Jayamoorthy, *Photochem. Photobiol. Sci*, **2013,** *12,* 1761-1773.
[3] D. Zhao, J. Hu, N. Wu, X. Huang, X. Qin, J. Lan, J. You, *Org. Lett.* **2011**, *13,* 6516-6519.
[4] P. Saha, Md Ashif Ali, P. Ghosh, T. Punniyamurthy , *Org. Biomol. Chem,* **2010**, *8,* 5692-5699.

[5] G. Brasche, S. L. Buchwald, N. Zheng, K. W. Anderson, X. Huang, H. N. Nguyen, S. L. Buchwald, *Angew. Chem. Int. Ed.* **2007**, *46*, 7509-7512; *Angew. Chem.* **2007**, *119*, 7653-7656.
[6] T. B. Nguyen, L. Ermolenko, W. A. Dean, A. Al-Mourabit, *Org. Lett.* **2012**, *14*, 5948-5951.
[7] R. Murakami, K. Tsunoda, T. Iwai, M. Sawamura, *Chem. Eur. J.*, **2014**, *20*, 13127-13131.
[8] J. Shi, E. Forsythe, D. C. Morton, US Patent US2013/172570 A1, **2013**.
[9] Eli Lilly and Company, WO1997/33873 A1; US Patent US6025379 A1, **2000**.
[10] Yu. F. Milyaev, S. A. Khorishko, L. N. Balyatinskaya, *J.Gen. Chem. USSR (Engl. Transl.)*, **1981**, *51,* 168-171 and 192-196.
[11] D. Secci, A. Bolasco, M. D'Ascenzio, F. della Sala, M. Yanez, S. Carradori, *J. Heterocyclic Chem.* **2012**, *49,* 1187-1195.
[12] S. Iyengar, M.A. Mulhauser, K.B. Thor, PCT Int. Appl. (1977), WO 9733873 A1, **1997**
[13] A. Spencer, *J. Organomet. Chem.,* **1985**, *295*, 79-89.

IV. Analytical data for new compounds

Benzimidazole 4i. Colorless crystal (recrystallized from petroleum ether) m.p. 120-124°C; ^{1}H NMR (300 MHz, $CDCl_3$, 25°C, TMS): δ = 7.28-7.35 (m, 3H, Ar), 7.41-7.53 (m, 8H, Ar), 7.98 (d, J = 9.0 Hz, 1H, Ar) ; ^{13}C NMR (75 MHz, $CDCl_3$, 25°C, TMS): δ = 110.7 (CH, Ar), 120.3 (CH, Ar), 123.2 (CH, Ar), 123.9 (CH, Ar), 126.6 (2 x CH, Ar), 127.2 (CH, Ar), 128.4 (CH, Ar), 128.8 (CH, Ar), 129.6 (2 x CH, Ar), 129.8 (CH, Ar), 133.3 (Cq), 135.0 (Cq), 135.8 (Cq), 135.9 (Cq), 136.5 (Cq), 142.8 (Cq), 149.4 (Cq); HRMS (ESI+) *m/z* calcd for $C_{19}H_{12}Cl_2N_2$ $[M+H]^+$ 339.0450. Found 339.0450.

Benzimidazole 4o. Colorless crystal (recrystallized from petroleum ether): m.p. 80-84°C; ^{1}H NMR (300 MHz, $CDCl_3$, 25°C, TMS): δ = 3.10-3.30 (m, 4H, 2 x CH_2), 7.13-7.36 (m, 10H, Ar), 7.57 (m, 3H, Ar), 7.88 (d, J = 7.9 Hz, 1H, Ar) ; ^{13}C NMR (75 MHz, $CDCl_3$, 25°C, TMS): δ = 29.8 (CH_2), 34.2 (CH_2), 110.2 (CH, Ar), 119.1 (CH, Ar), 122.6 (CH, Ar), 122.8 (CH, Ar), 126.3 (CH, Ar), 127.4 (2 x CH, Ar), 128.4 (2 x CH, Ar), 128.5 (2 x CH, Ar), 129.0 (CH, Ar), 129.9 (2 x CH, Ar), 135.7 (Cq), 136.4 (Cq), 140.8 (Cq), 142.2 (Cq), 154.3 (Cq); HRMS (ESI+) *m/z* calcd for $C_{21}H_{18}N_2$ $[M+H]^+$ 299.1548. Found 299.1548.

Benzimidazole 4p. Pale yellow oil: ^{1}H NMR (300 MHz, $CDCl_3$, 25°C, TMS): δ = 0.85-0.89 (t, J = 7.0 Hz, 3H, *n*-octyl), 1.25-1.40 (m, 8H, *n*-octyl), 1.76-7.86 (m, 2H, *n*-octyl), 2.78-2.83 (t, J = 7.0 Hz, 2H, *n*-octyl), 7.10 (d, J = 7.5 Hz, 1H, Ar), 7.20 (t, J = 7.5 Hz, 1H, Ar), 7.3 (t, J = 7.0 Hz, 1H, Ar), 7.36 (d, J = 7 Hz, 2H, Ar), 7.57 (m, 3H, Ar), 7.82 (d, J = 7.0 Hz, 1H, Ar) ; ^{13}C NMR (75 MHz, $CDCl_3$, 25°C, TMS): δ = 14.1 (CH_3), 22.6 (CH_2), 27.7 (CH_2), 27.8 (CH_2), 28.9 (CH_2), 29.3 (CH_2), 31.6 (CH_2), 109.9 (CH, Ar), 119.1 (CH, Ar), 122.3 (CH, Ar), 122.5 (CH, Ar), 127.4 (2 x CH, Ar), 128.9 (CH, Ar), 129.9 (2 x CH, Ar), 136.1 (Cq), 136.5 (Cq), 142.6 (Cq), 155.4 (Cq); HRMS (ESI+) *m/z* calcd for $C_{20}H_{24}N_2$ $[M+H]^+$ 293.2018. Found 293.2018.

Benzimidazole 4r. Colorless crystal (recrystallized from petroleum ether) : m.p. 120-124°C, ^{1}H NMR (300 MHz, $CDCl_3$, 25°C, TMS): δ = 0.96 (d, J = 6.6 Hz, 6H, isobutyl),

2.20-2.29 (m, 1H, isobutyl), 2.73-2.76 (d, *J* = 6.6 Hz, 2H, isobutyl), 7.15 (d, *J* = 7.9 Hz, 1H, Ar), 7.25 (t, *J* = 7.1 Hz, 1H, CH, Ar), 7.35 (m, 1H, Ar), 7.4 (d, *J* = 7.1 Hz, 2H, Ar), 7.58-7.64 (m, 3H, Ar), 7.85 (d, *J* = 7.9 Hz, 1H, Ar) ; ^{13}C NMR (75 MHz, $CDCl_3$, 25°C, TMS): δ = 22.5 (2 x CH_3) , 28.0 (CH, isobutyl), 36.4 (CH_2), 110.1 (CH, Ar), 119.0 (CH, Ar), 122.5 (CH, Ar), 122.7 (CH, Ar), 127.6 (2 x CH, Ar), 129.0 (CH, Ar), 130.0 (2 x CH, Ar), 136.0 (Cq), 136.4 (Cq), 142.1 (Cq), 154.6 (Cq); HRMS (ESI+) *m/z* calcd for $C_{17}H_{18}N_2$ $[M+H]^+$ 251.1548. Found 251.1560.

Benzimidazole 4w. Colorless crystal (recrystallized from petroleum ether) : mp 92-96°C ^{1}H NMR (300 MHz, $CDCl_3$, 25°C, TMS): δ = 1.04-1.10 (m, 2H, cyclopropyl), 1.34-1.39 (m, 2H, cyclopropyl), 1.85 (m, 1H, cyclopropyl), 7.12-7.30 (m, 3H, Ar), 7.45 (d, *J* = 8.6 Hz, 2H, Ar), 7.58 (d, *J* = 8.6 Hz, 2H, Ar), 7.75 (d, *J* = 7.9 Hz, 1H, Ar) ; ^{13}C NMR (75 MHz, $CDCl_3$, 25°C, TMS): δ = 8.3 (CH), 9.7 (2 x CH_2), 109.4 (CH, Ar), 119.0 (CH, Ar), 122.5 (CH, Ar), 122.7 (CH, Ar), 128.6 (2 x CH, Ar), 130.1 (2 x CH, Ar), 134.52 (Cq), 134.55 (Cq), 136.3 (Cq),142.4 (Cq), 156.6 (Cq); HRMS (ESI+) *m/z* calcd for $C_{16}H_{13}ClN_2$ $[M+H]^+$ 269.0846. Found 269.0841.

V.High field ^{1}H NMR spectra and 1D proton-decoupled ^{13}C NMR spectra for all compounds 4a-w *(see next page)*

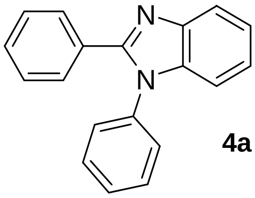

4a

1D proton NMR spectrum

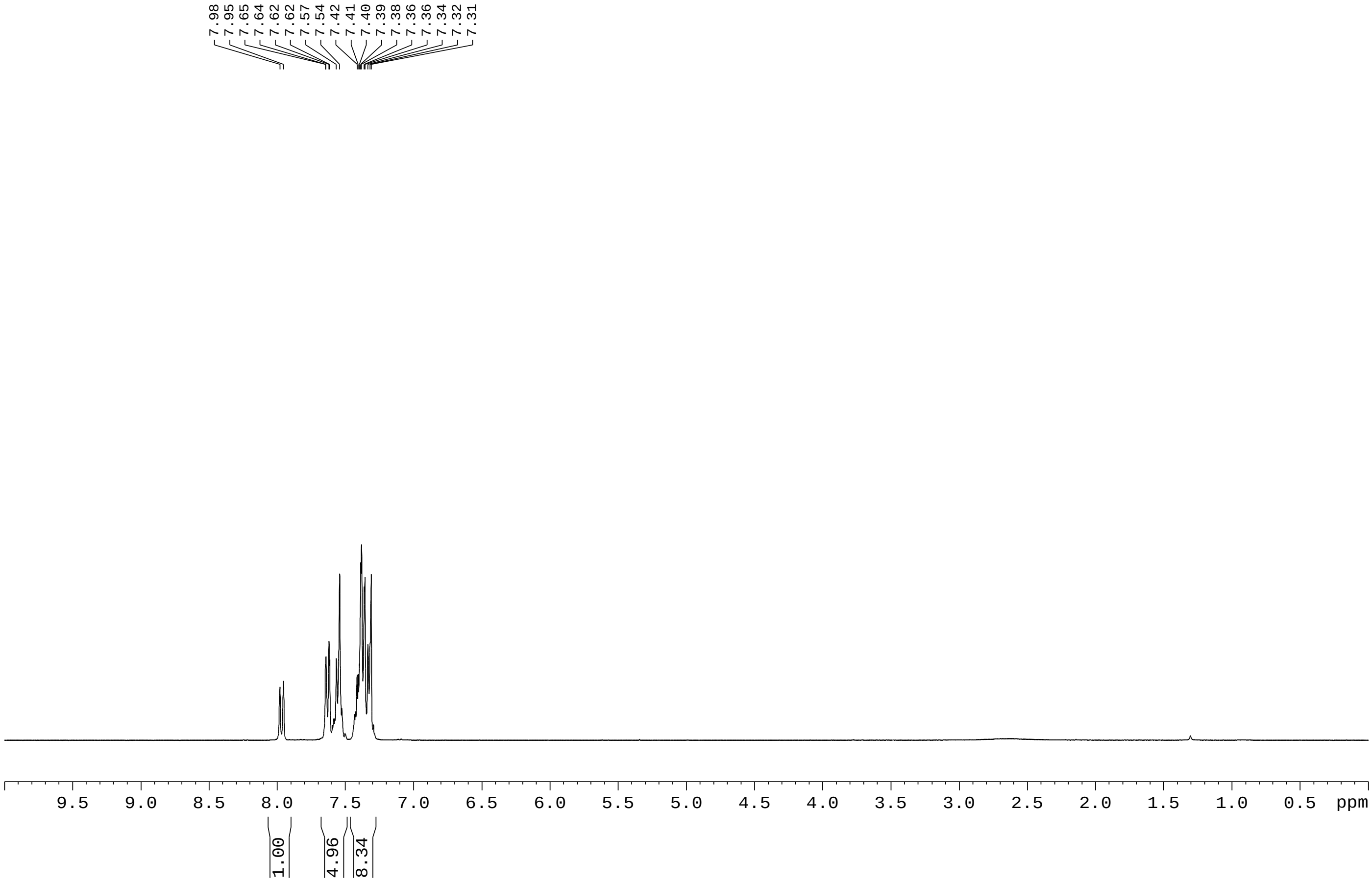

Proton-decoupled carbon NMR spectrum

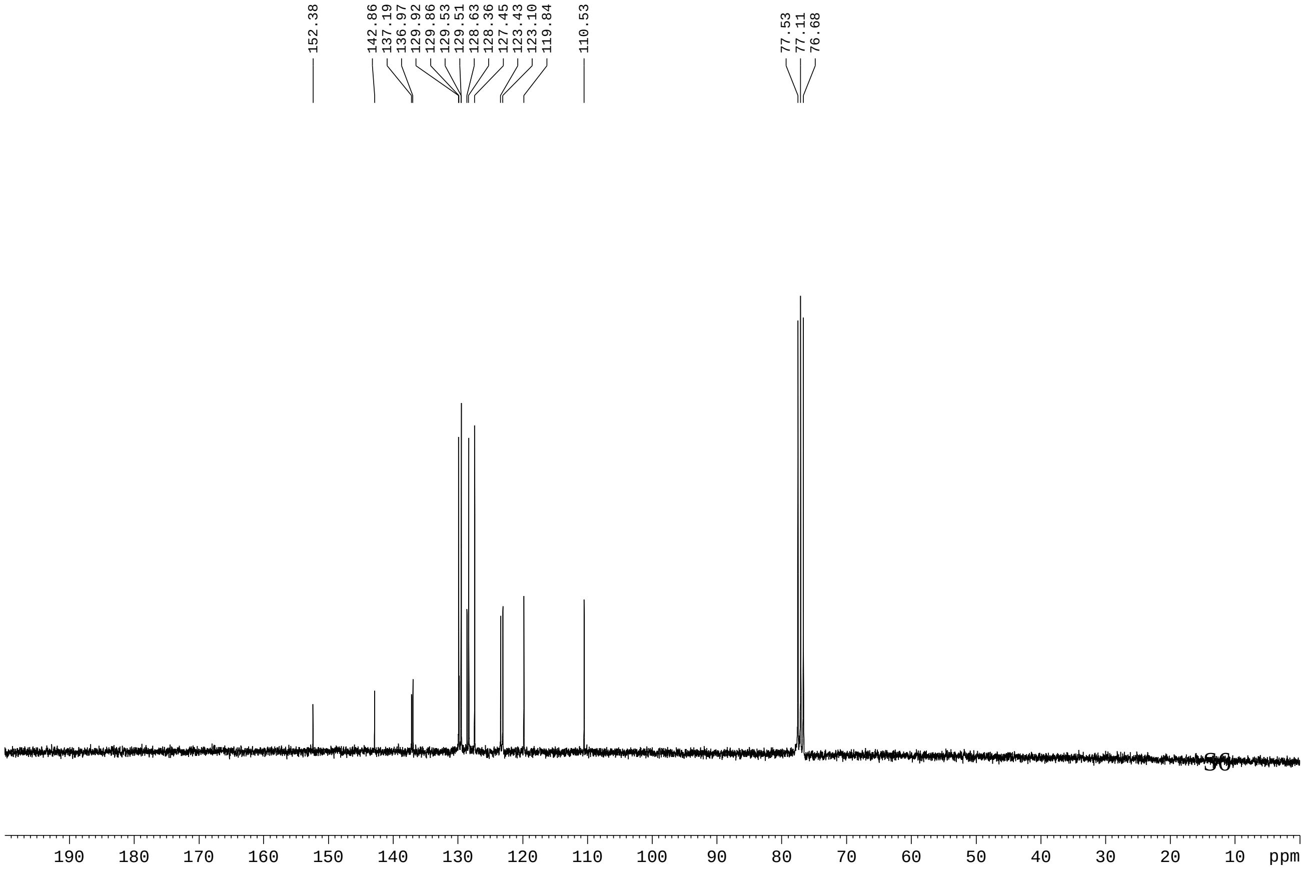

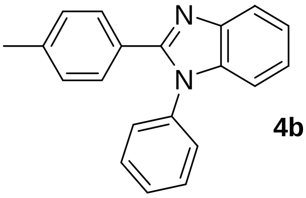

4b

1D proton NMR spectrum

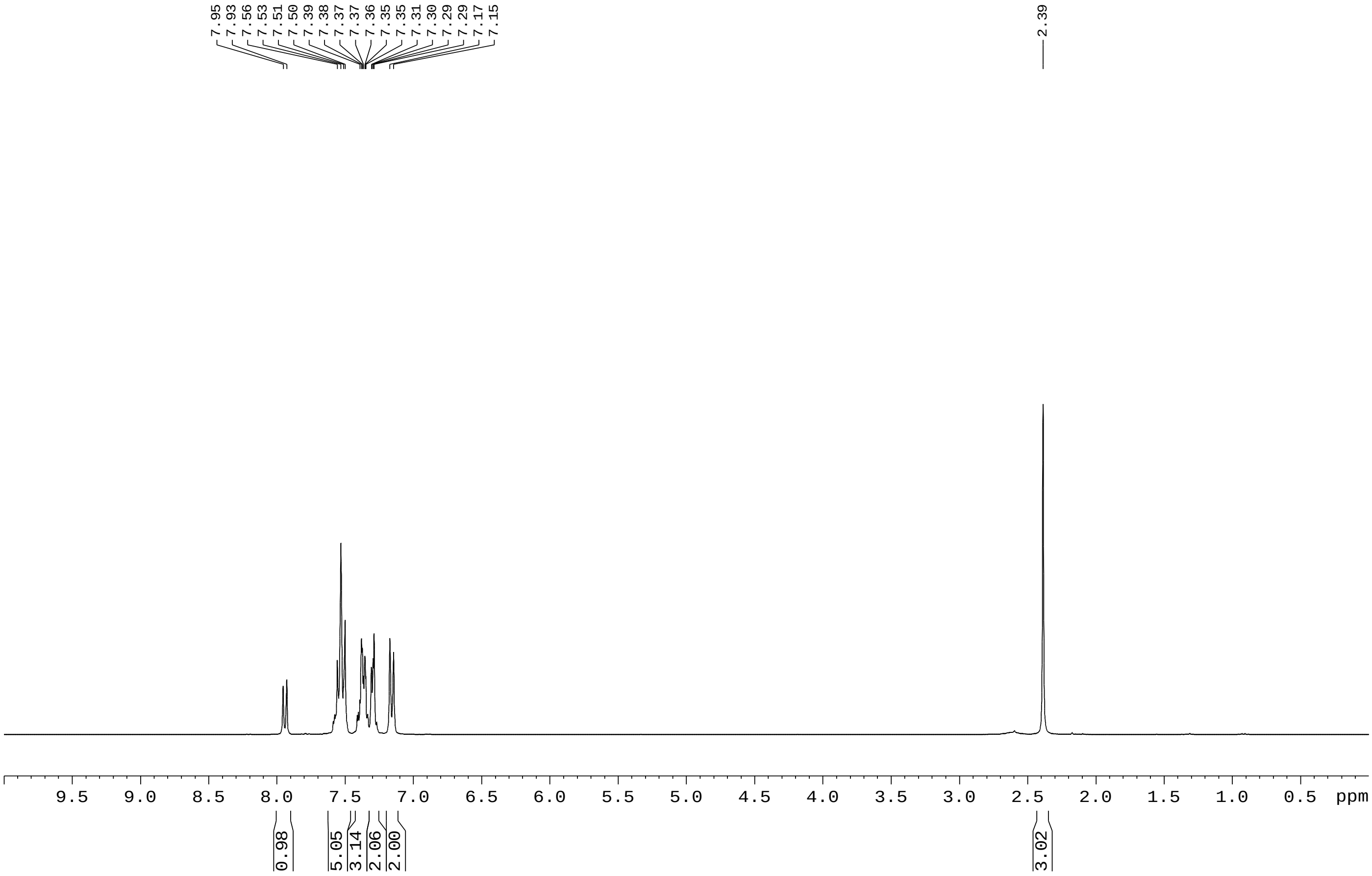

Proton-decoupled carbon NMR spectrum

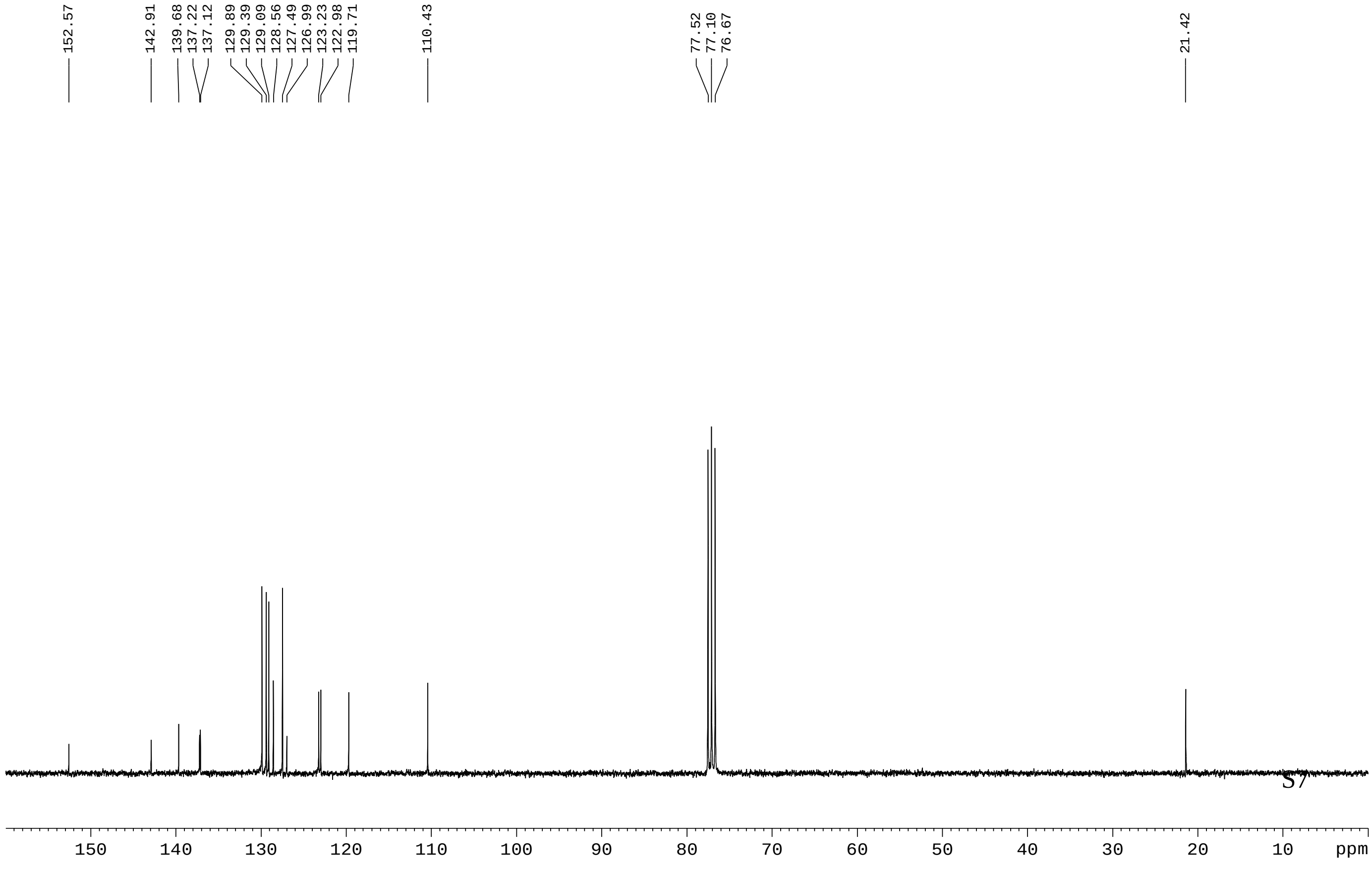

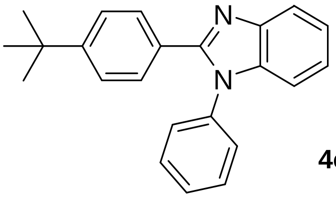

4c

1D proton NMR spectrum

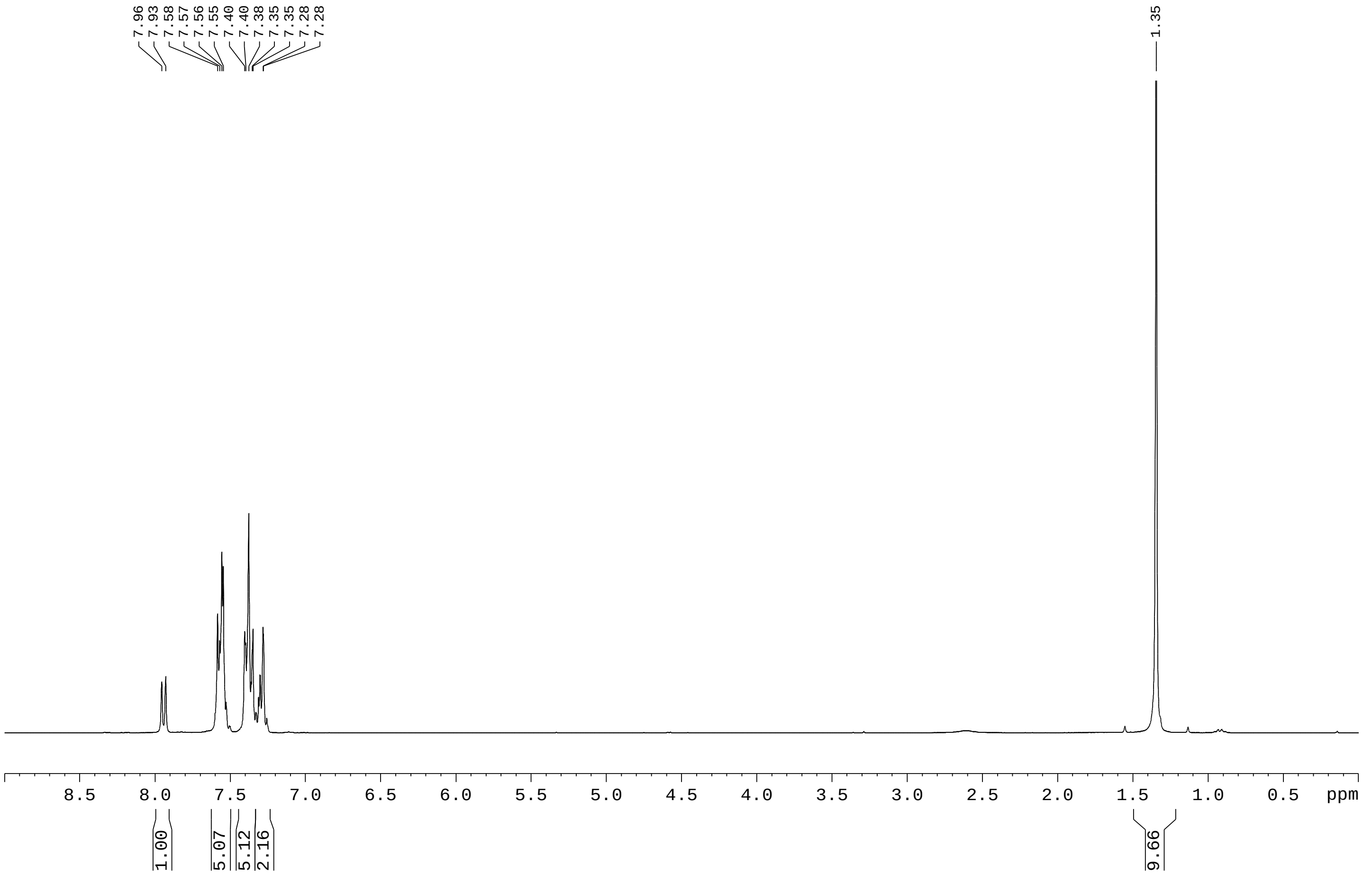

Proton-decoupled carbon NMR spectrum

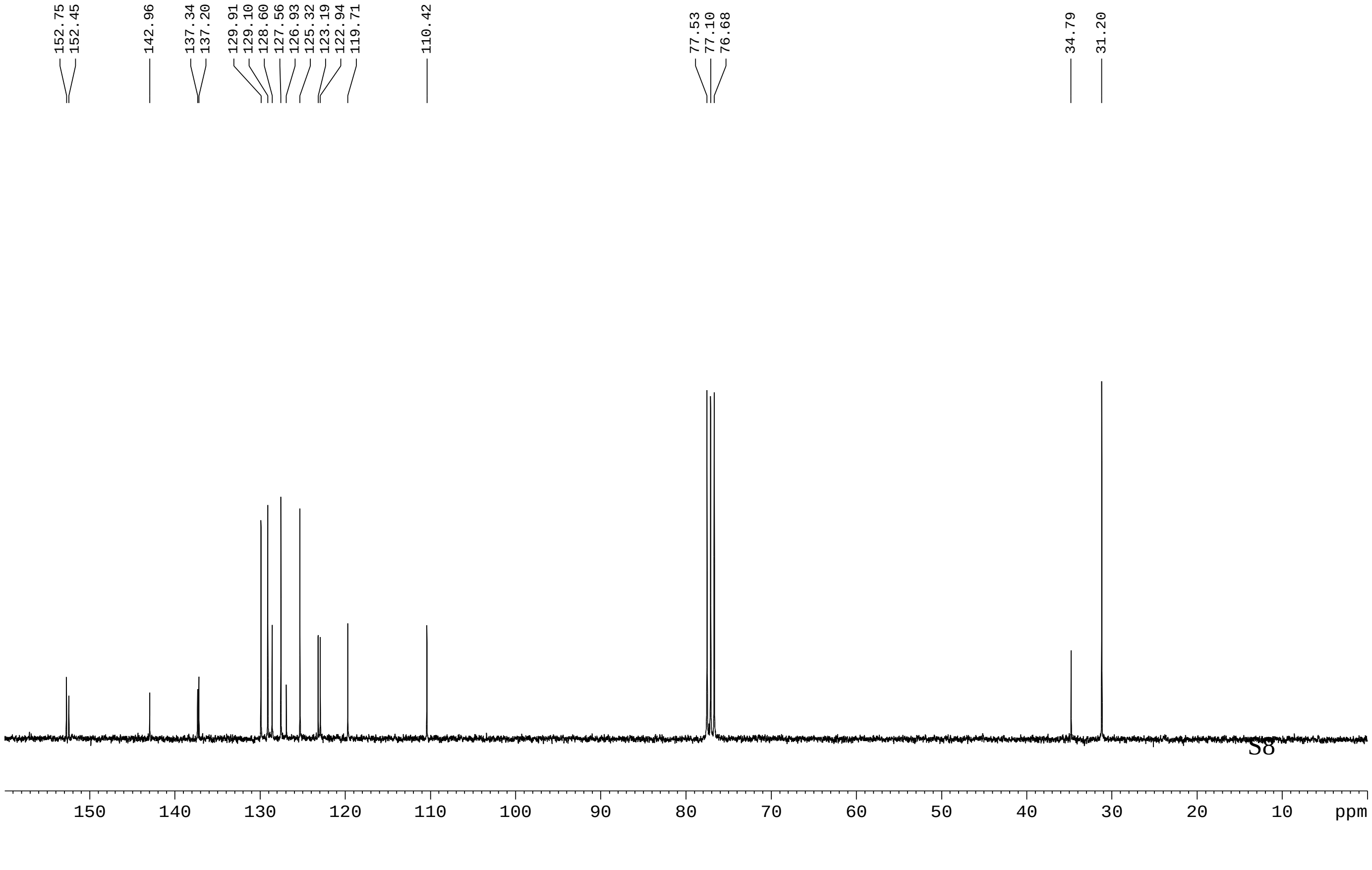

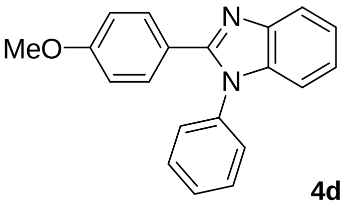

4d

1D proton NMR spectrum

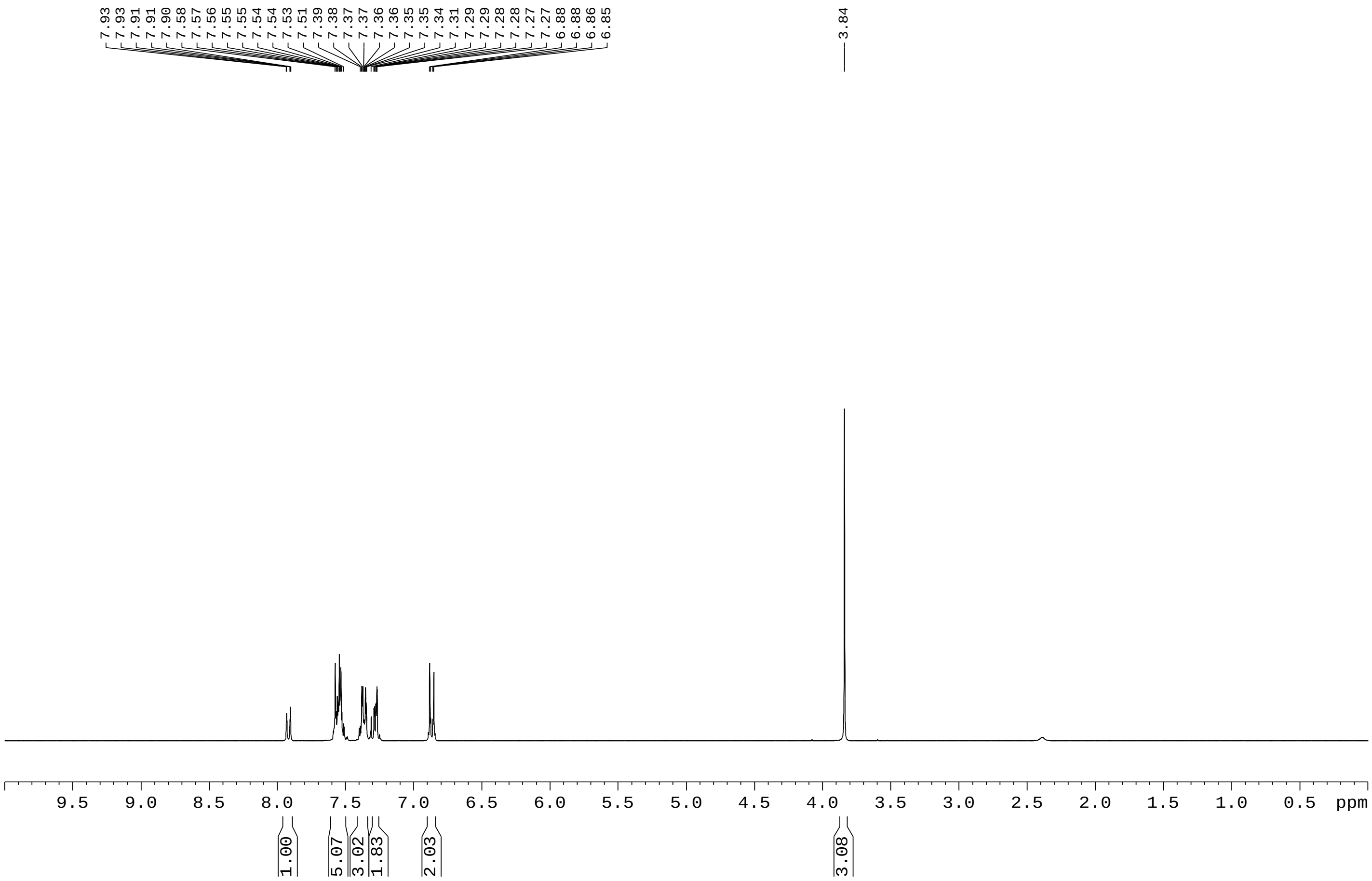

Proton-decoupled carbon NMR spectrum

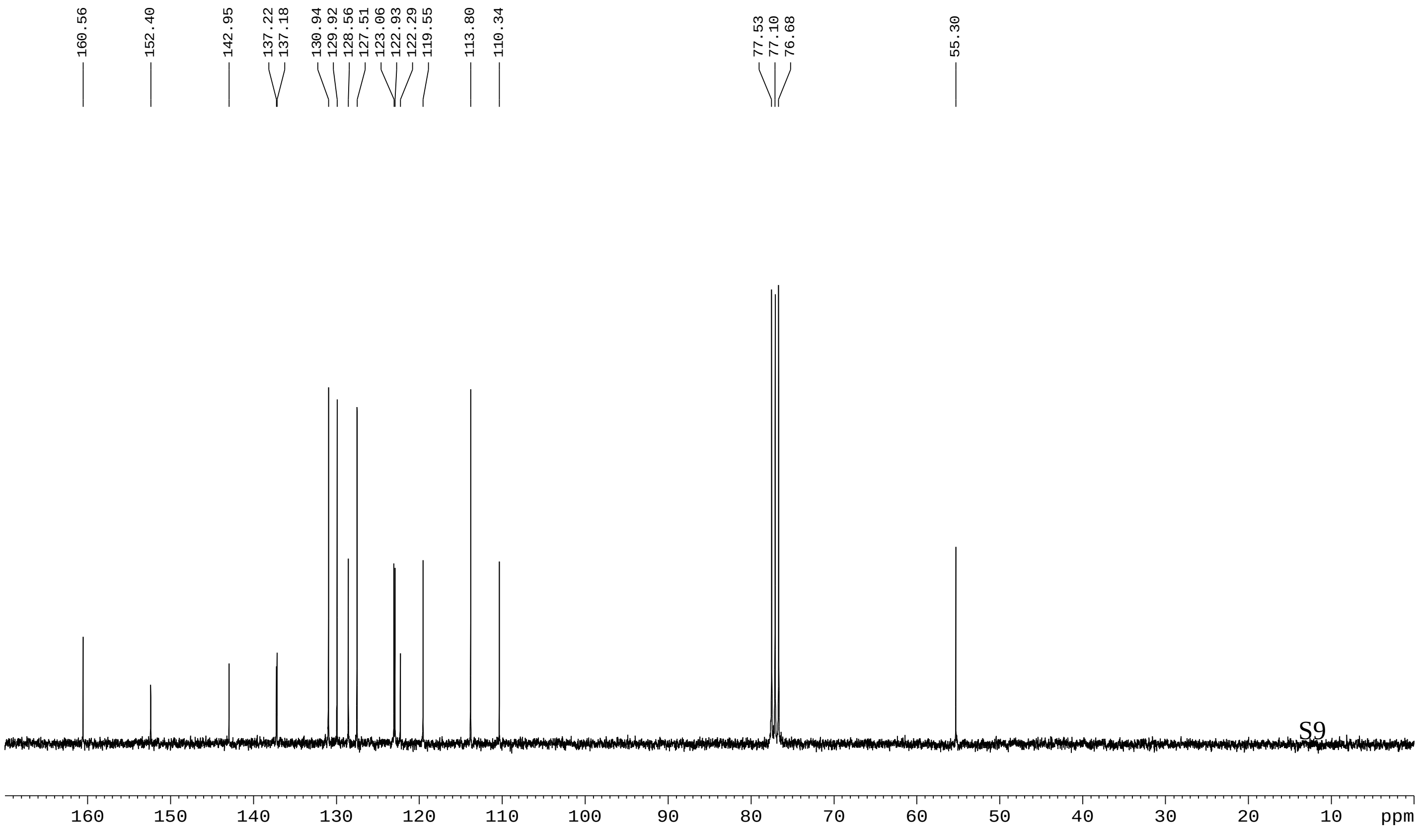

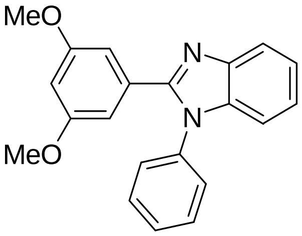

4e

1D proton NMR spectrum

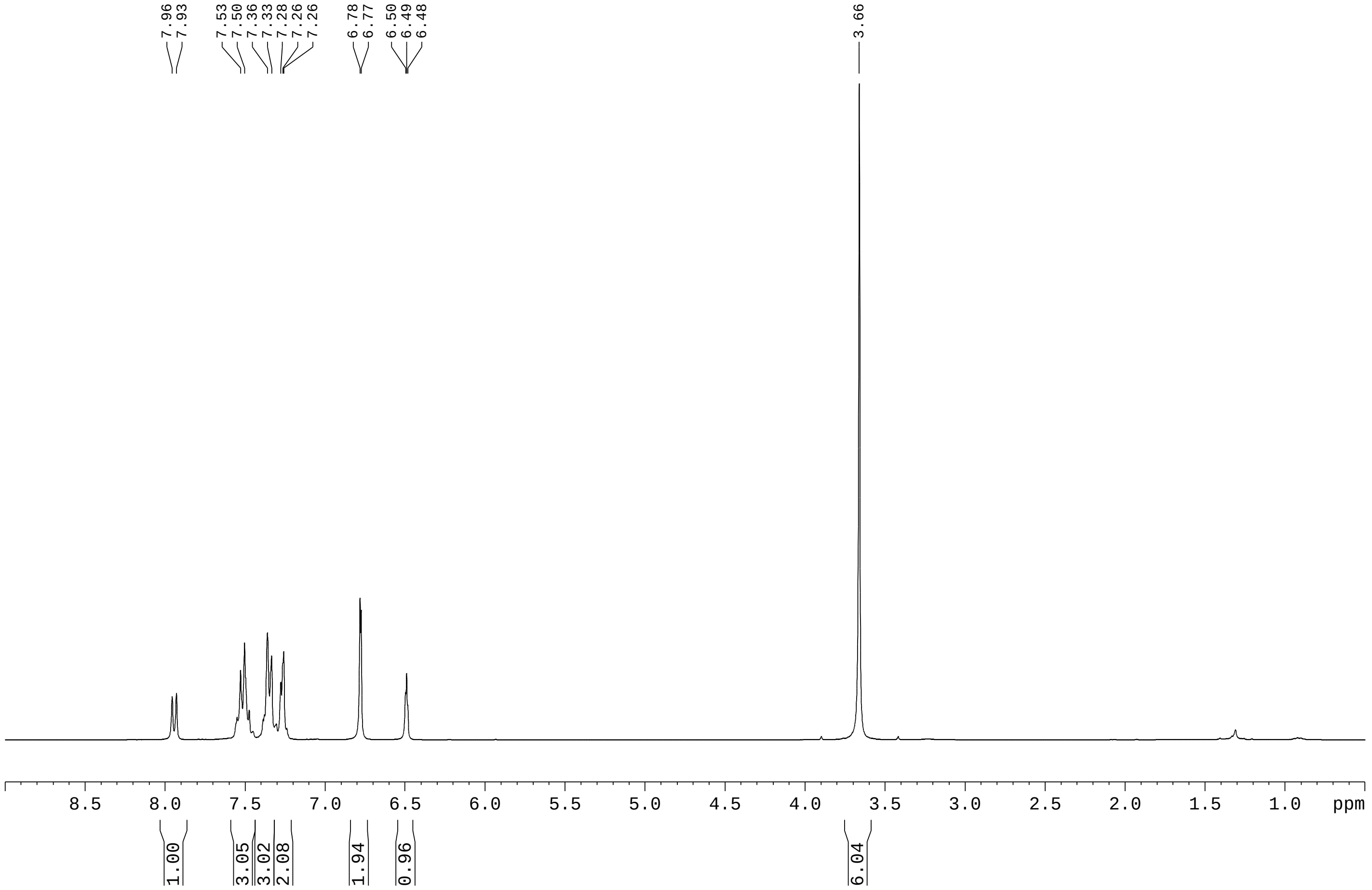

Proton-decoupled carbon NMR spectrum

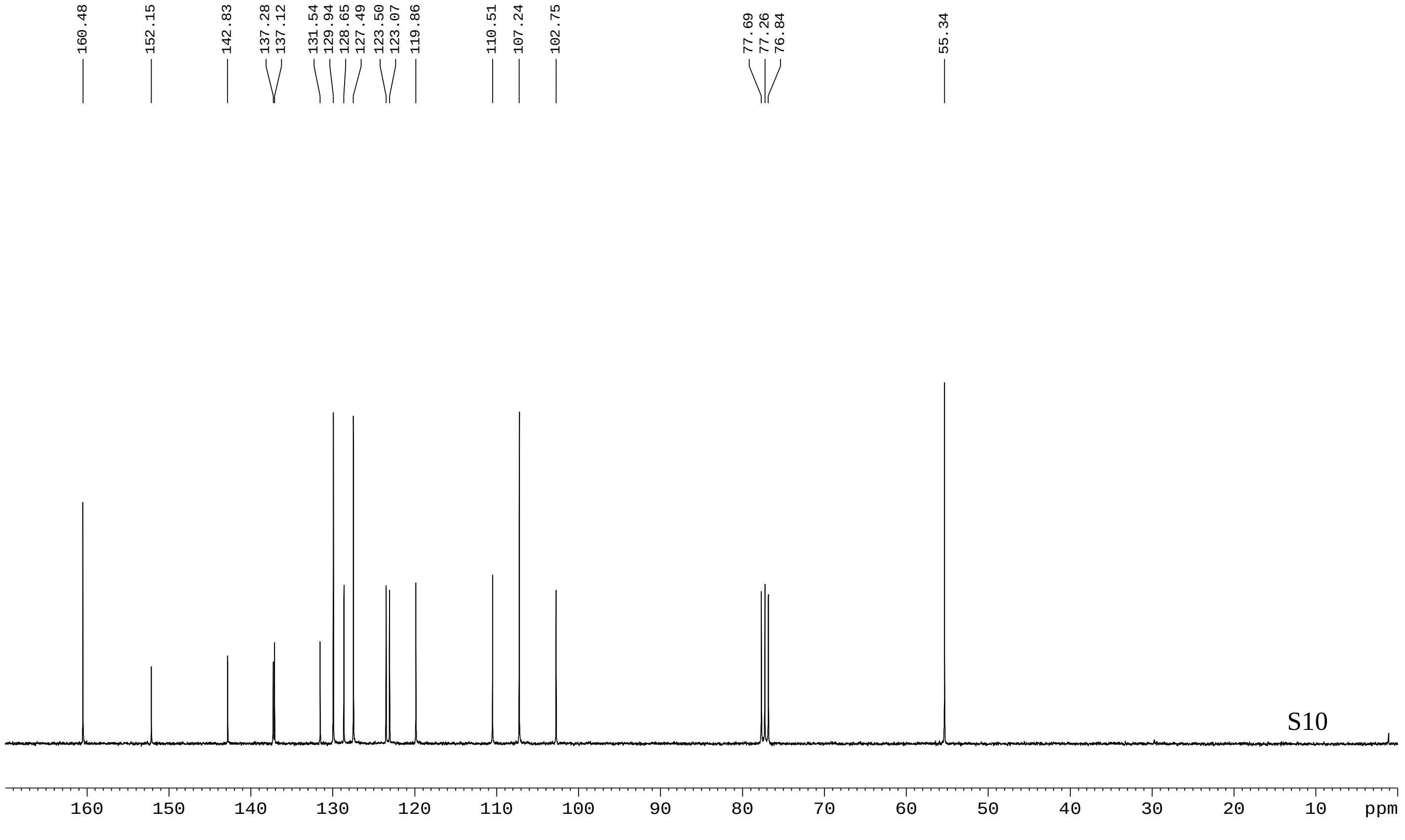

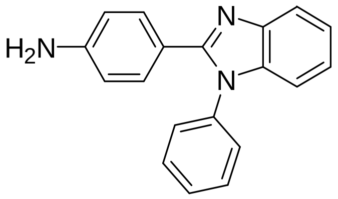

1D proton NMR spectrum

4f

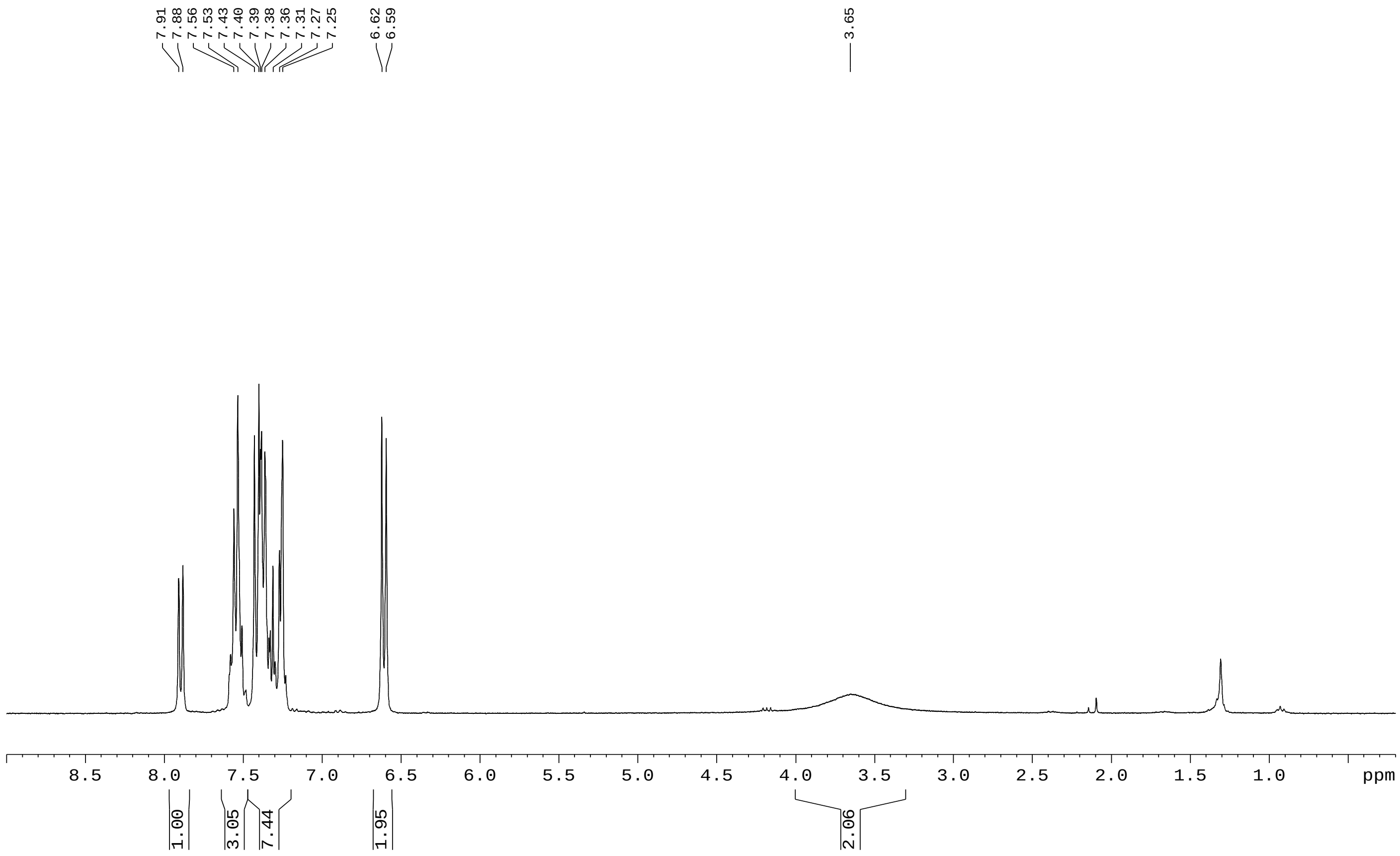

Proton-decoupled carbon NMR spectrum

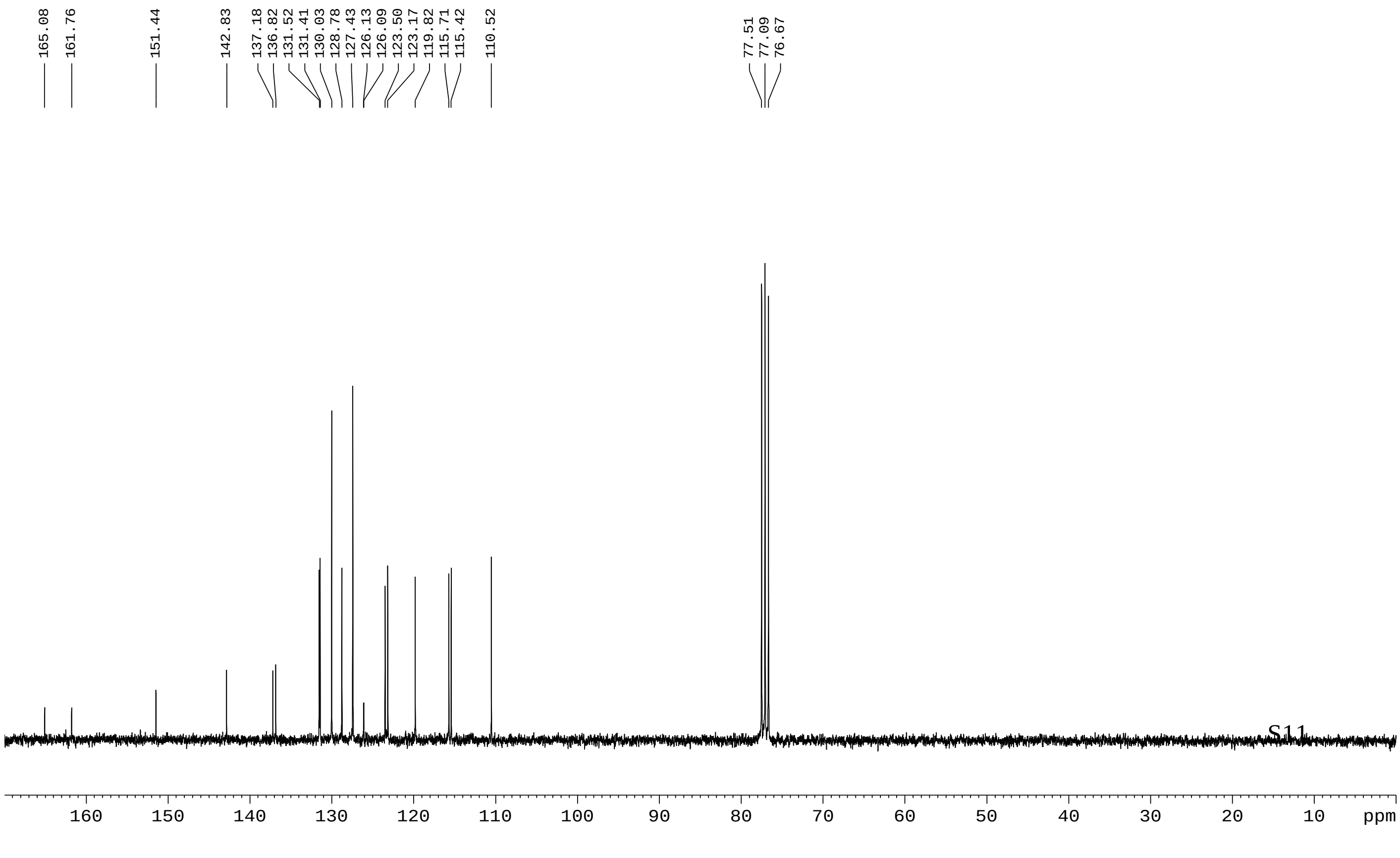

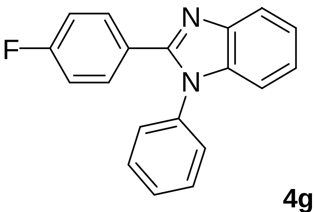

4g

1D proton NMR spectrum

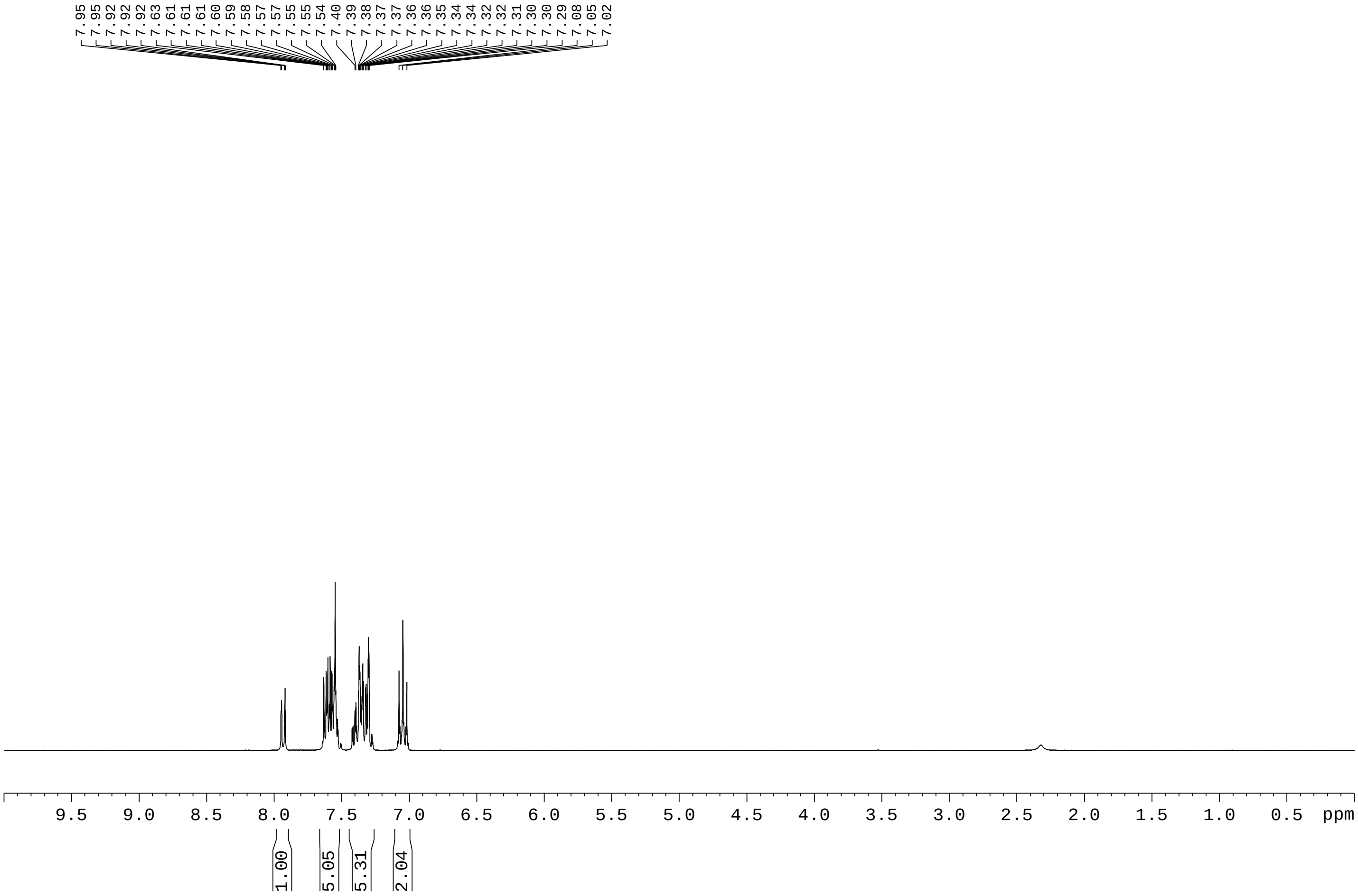

Proton-decoupled carbon NMR spectrum

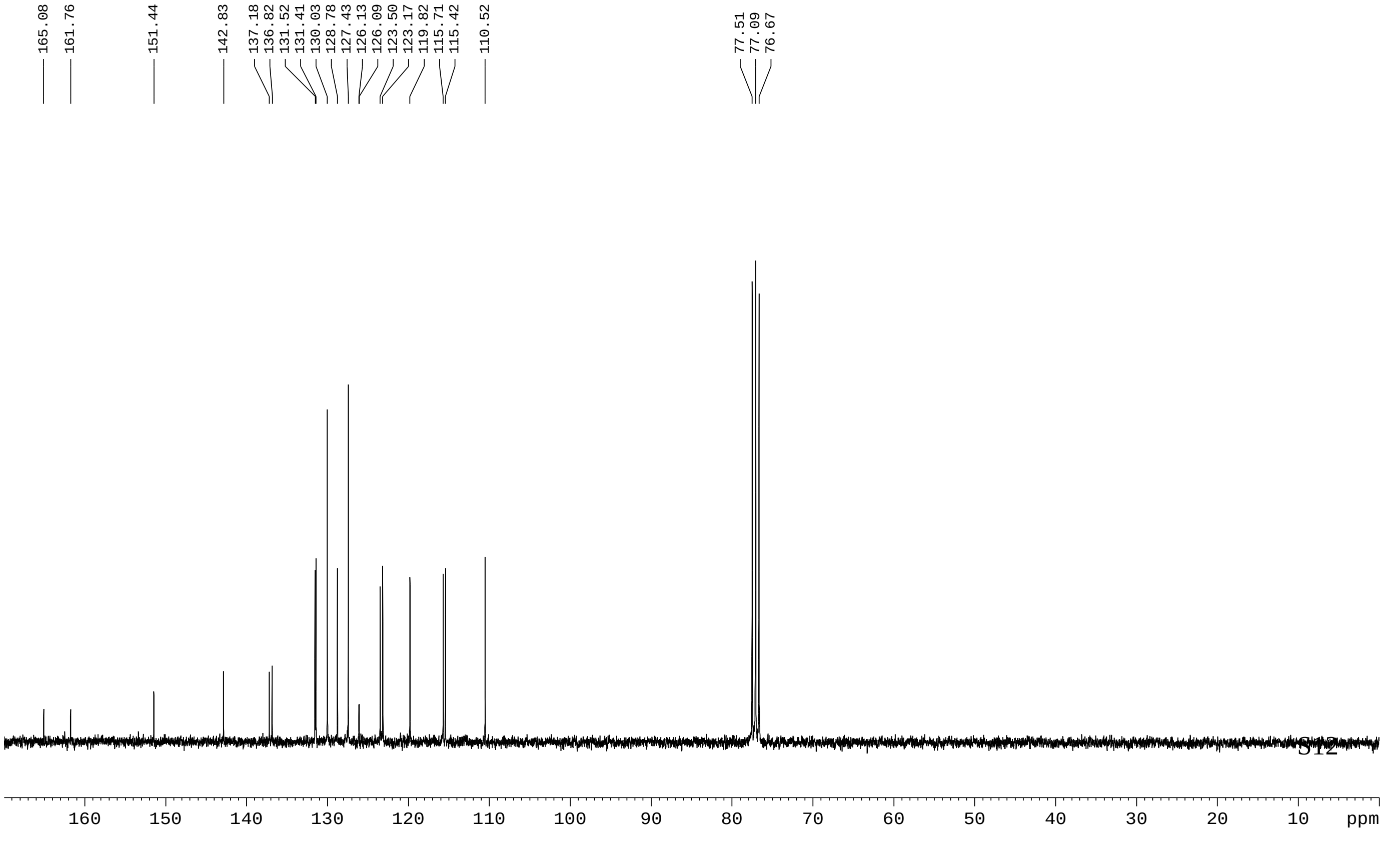

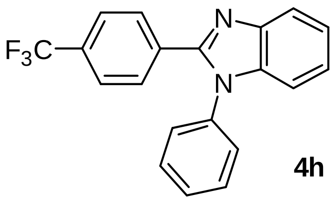

4h

1D proton NMR spectrum

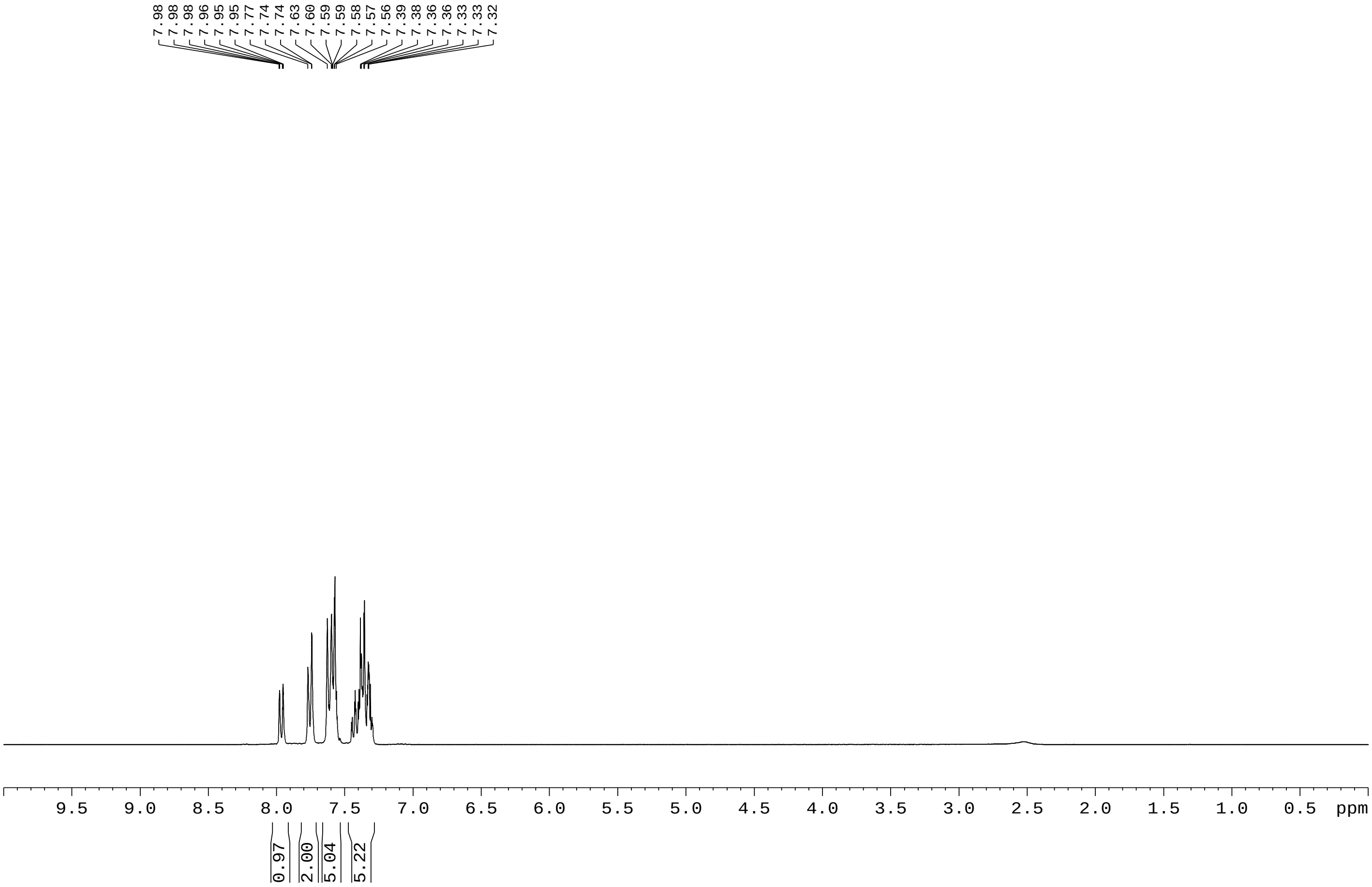

Proton-decoupled carbon NMR spectrum

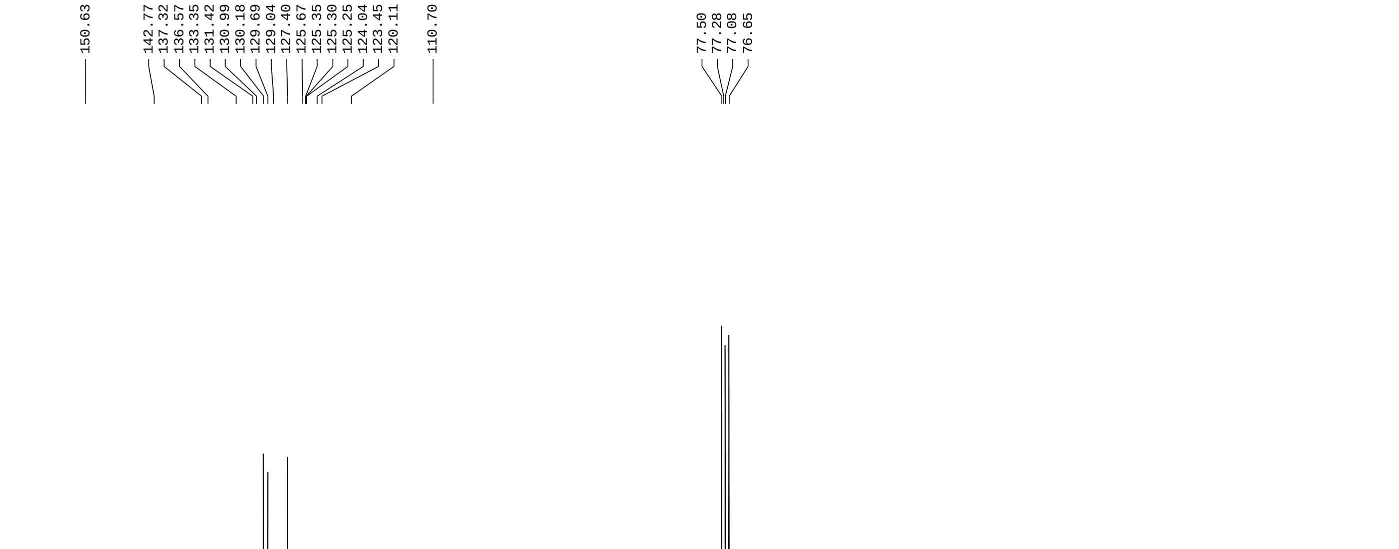

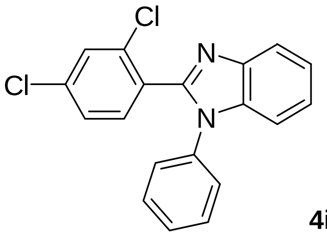

4i

1D proton NMR spectrum

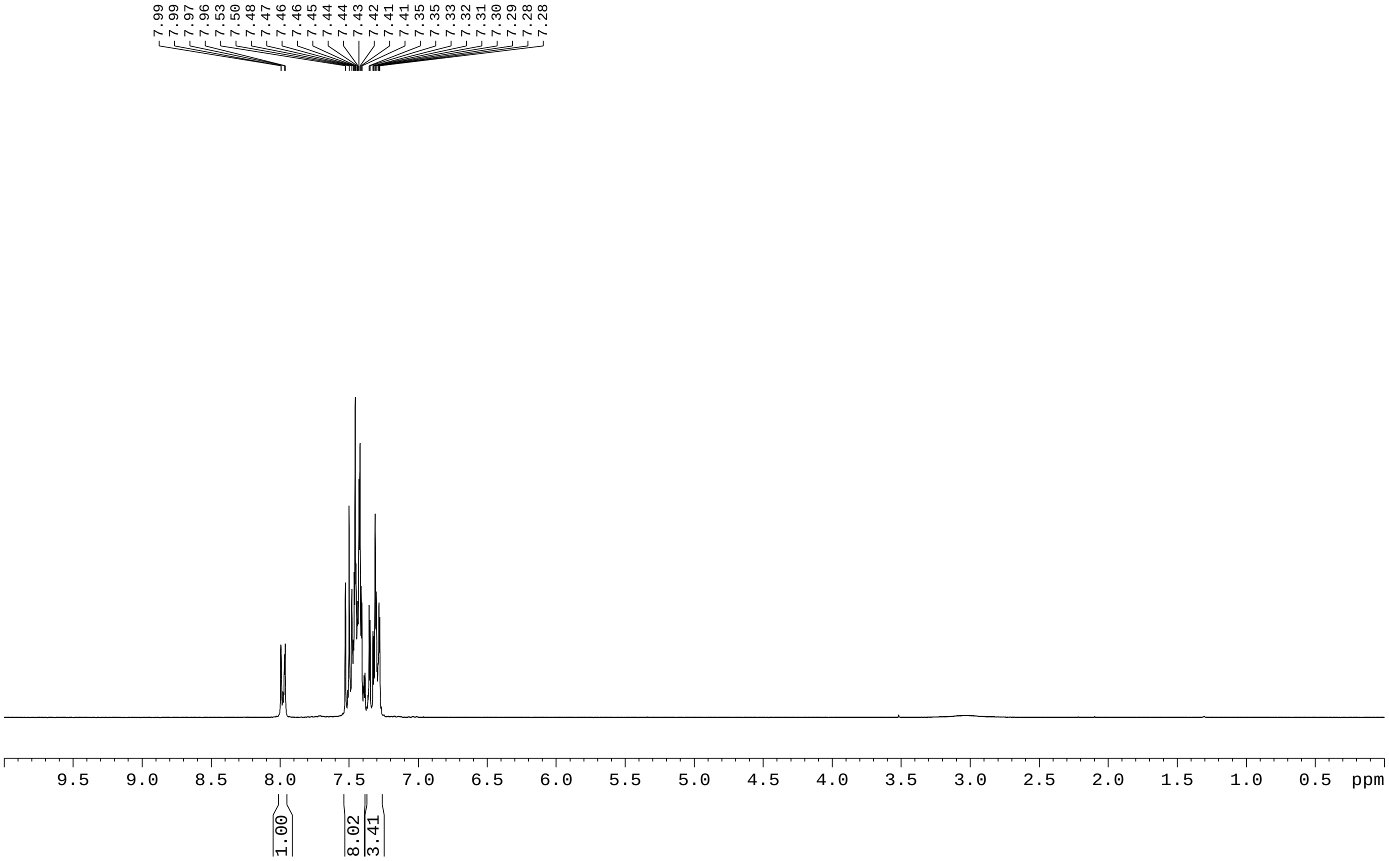

Proton-decoupled carbon NMR spectrum

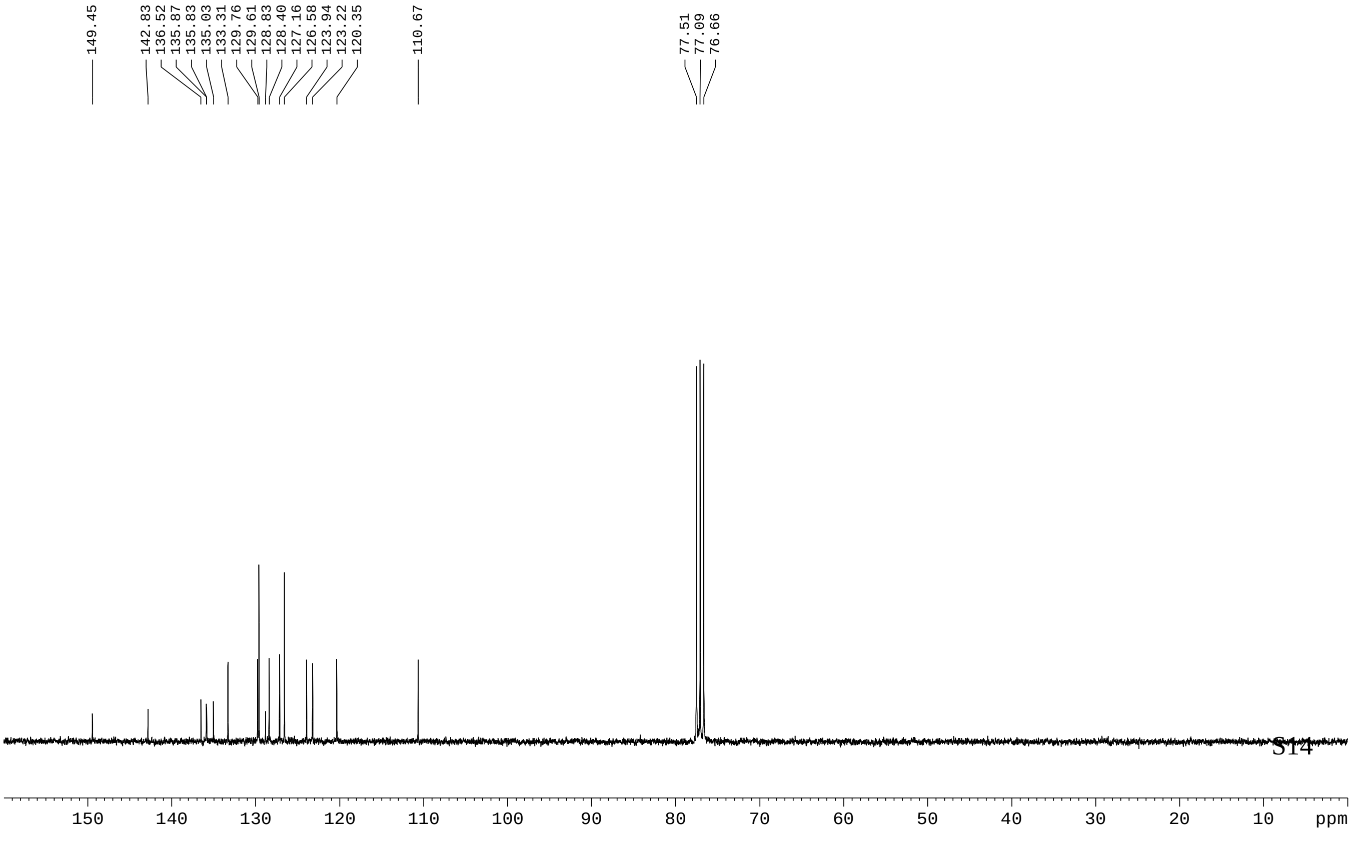

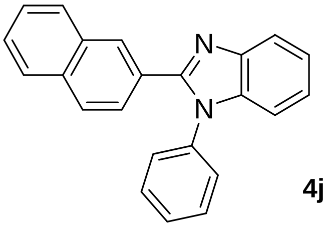

4j

1D proton NMR spectrum

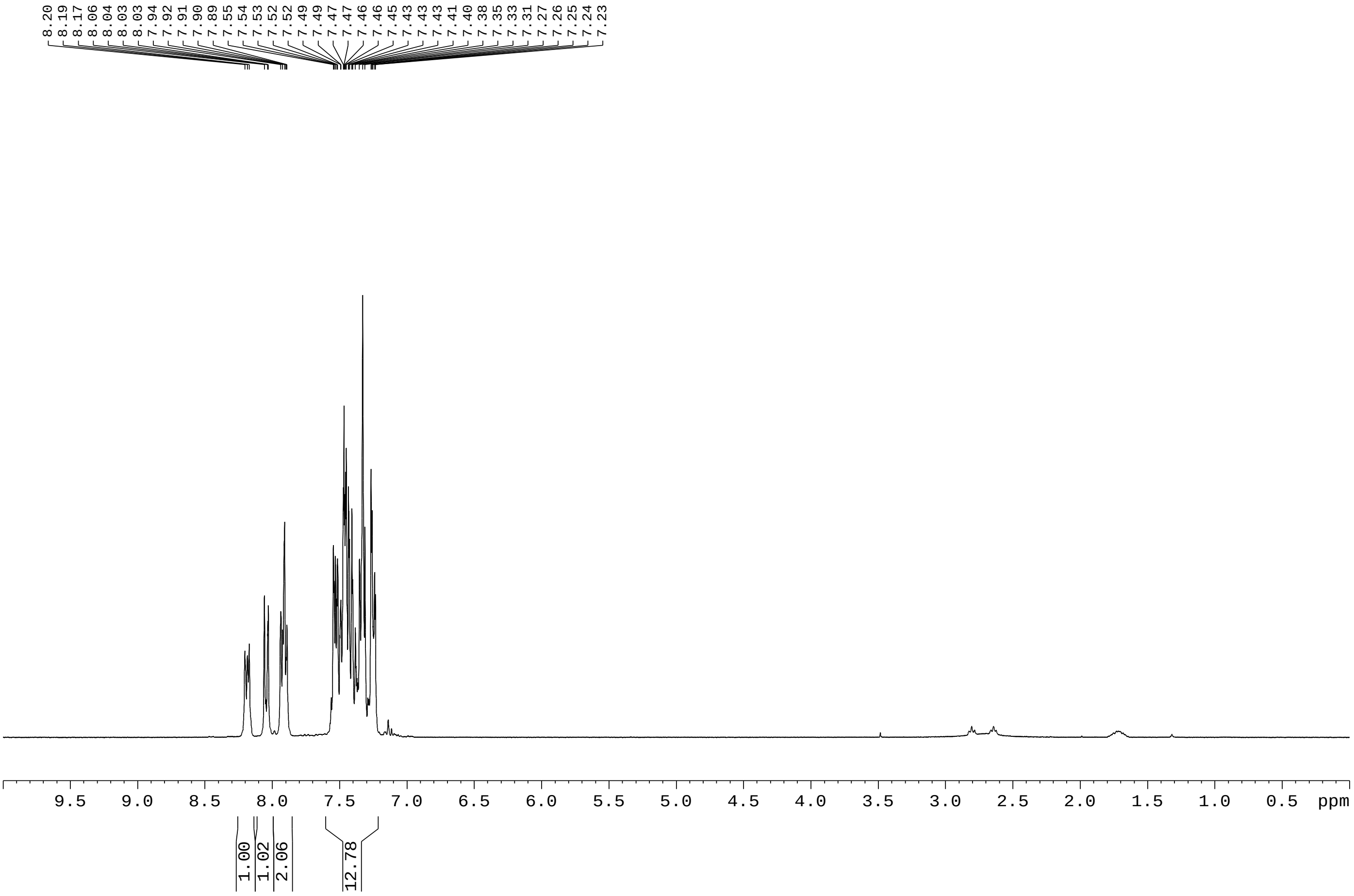

Proton-decoupled carbon NMR spectrum

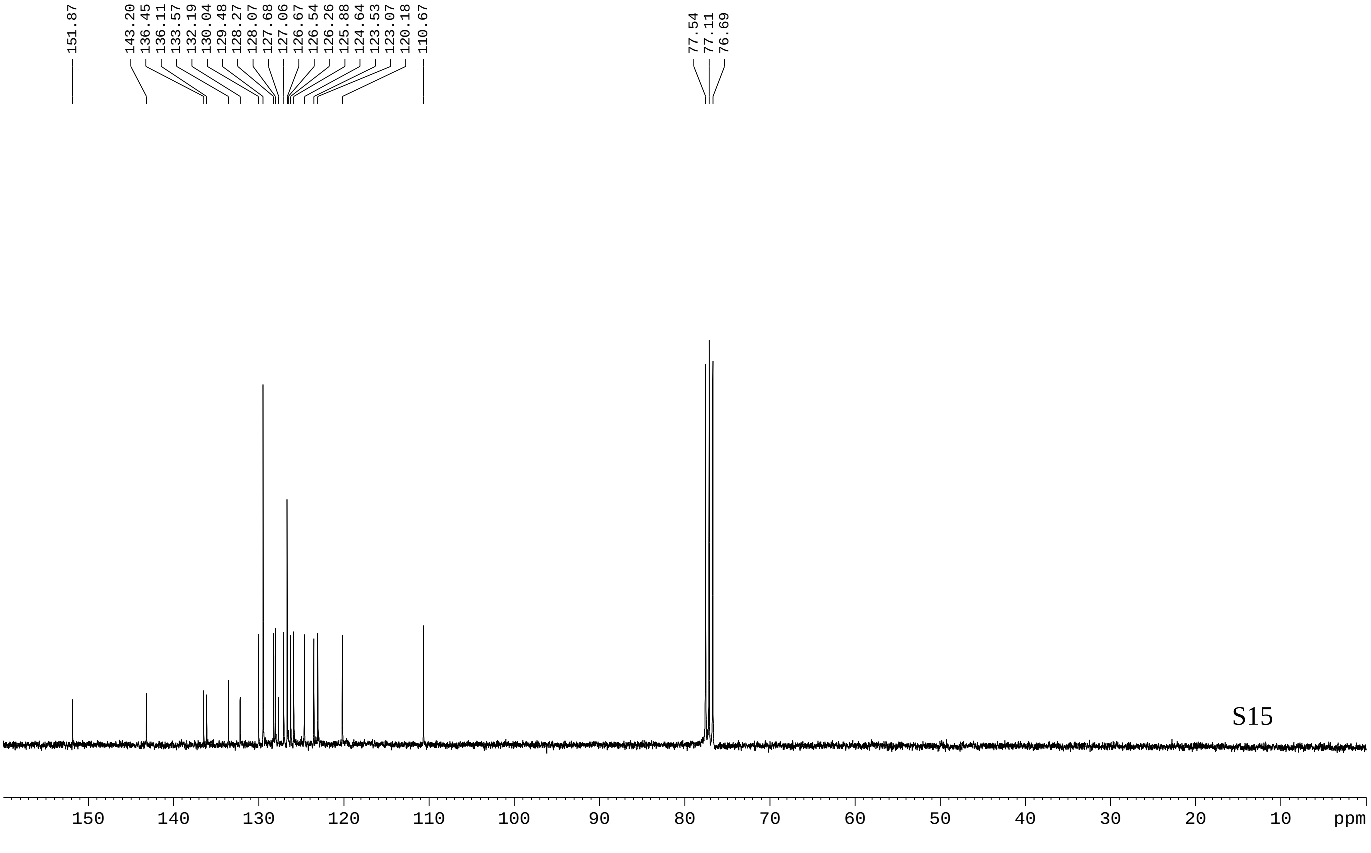

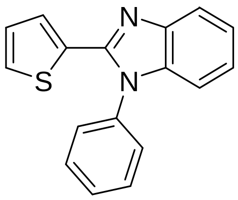

4k

1D proton NMR spectrum

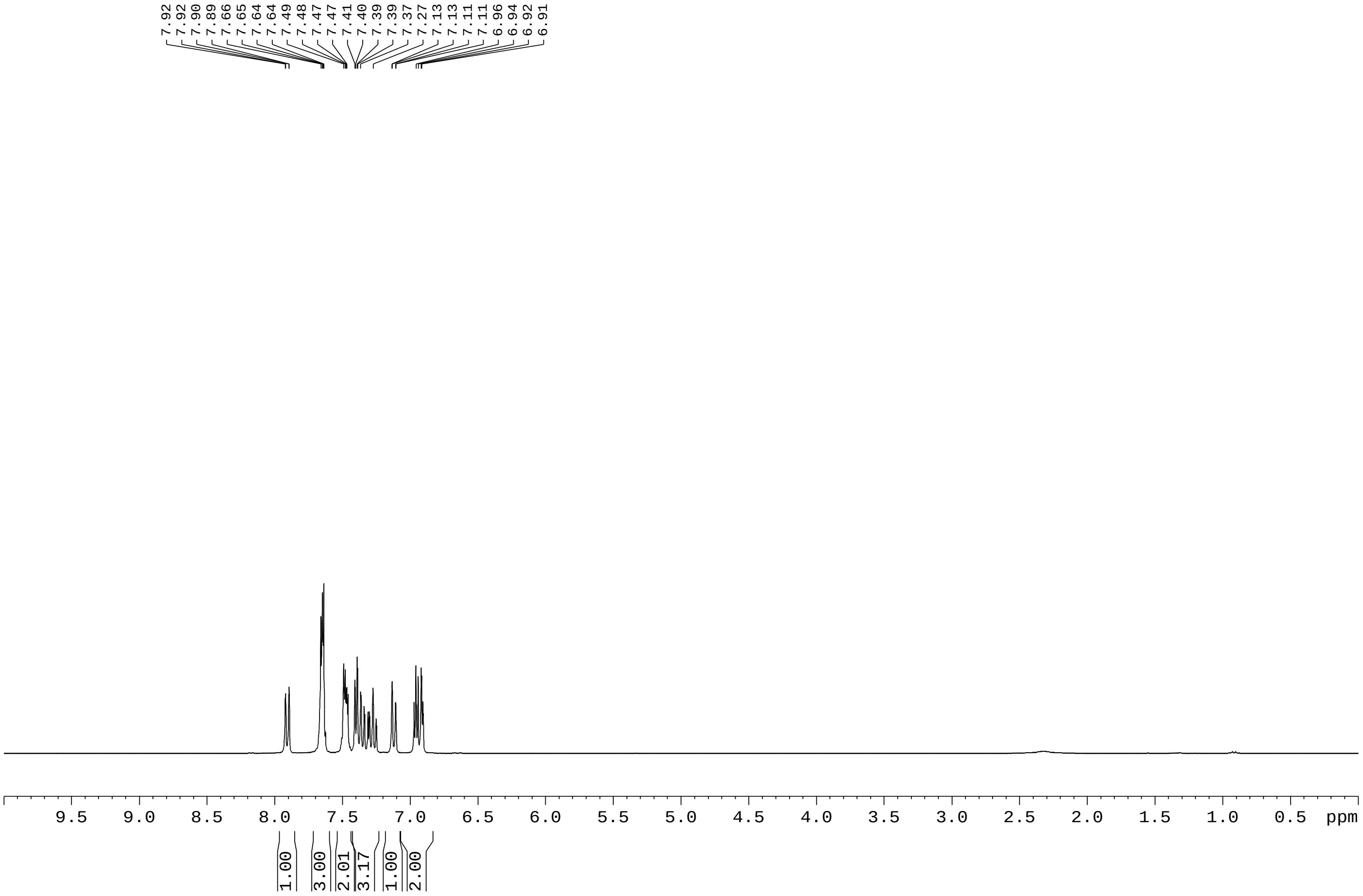

Proton-decoupled carbon NMR spectrum

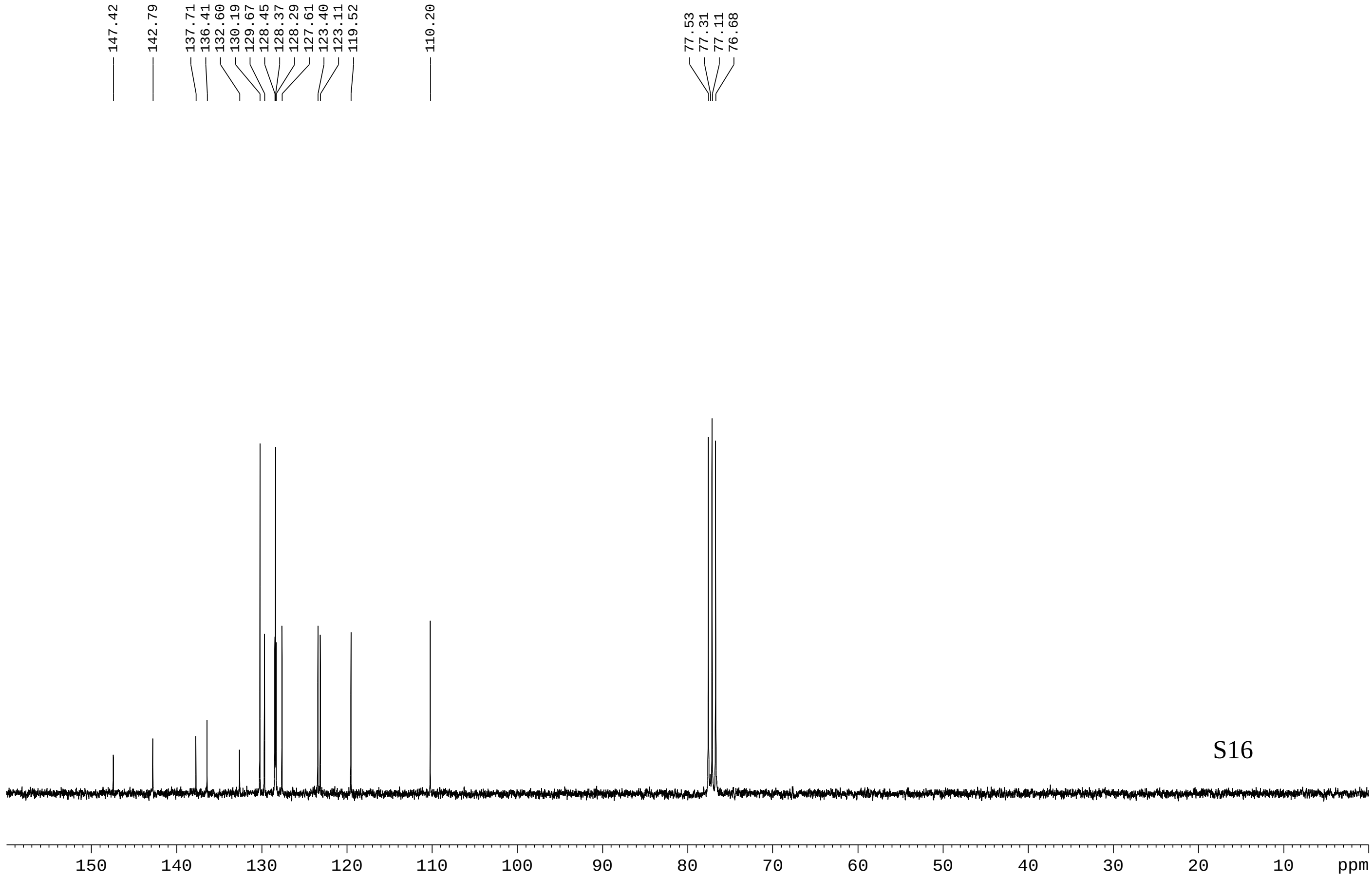

1D proton NMR spectrum

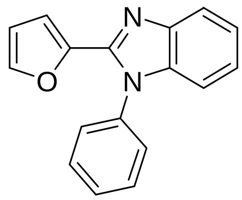

4l

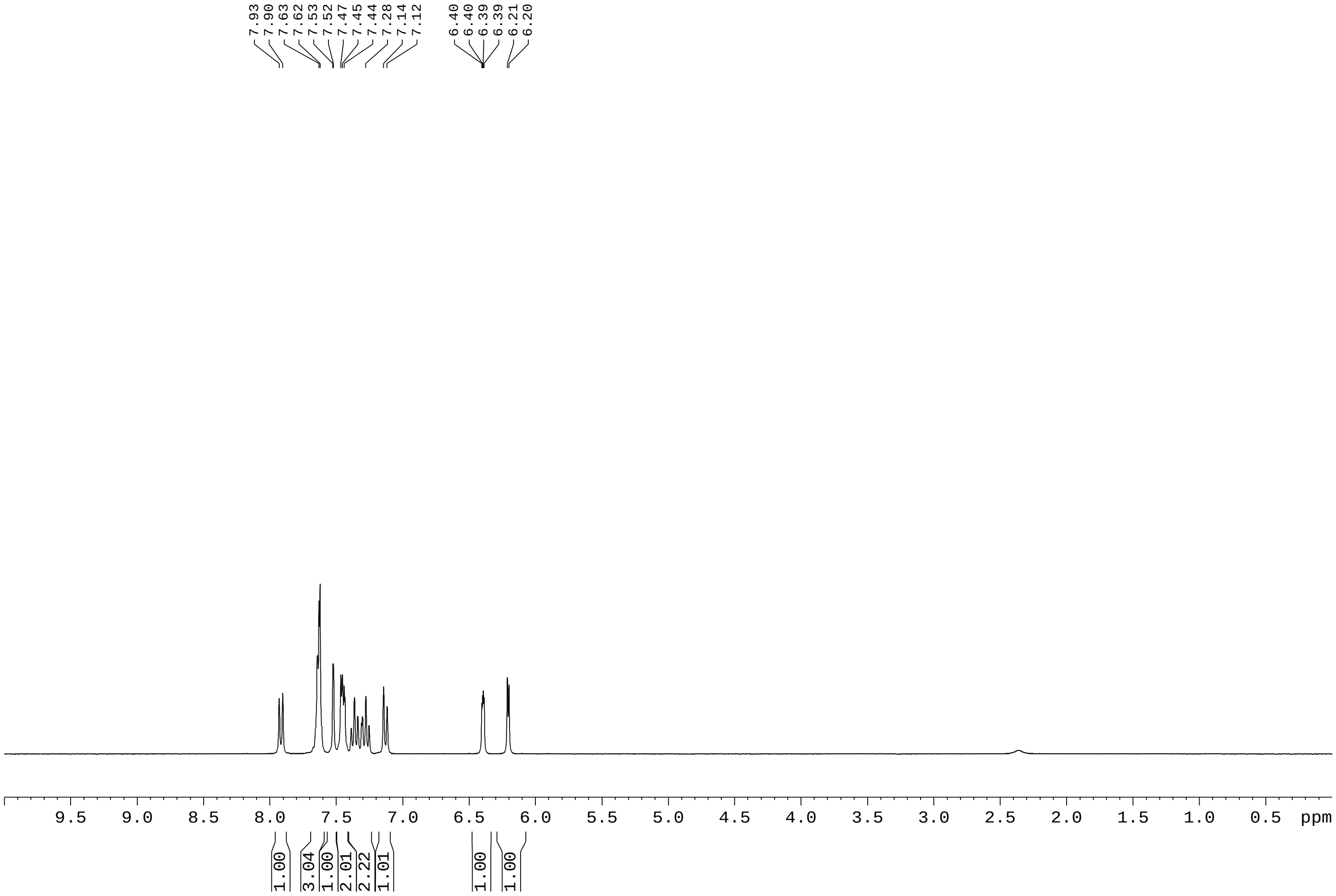

Proton-decoupled carbon NMR spectrum

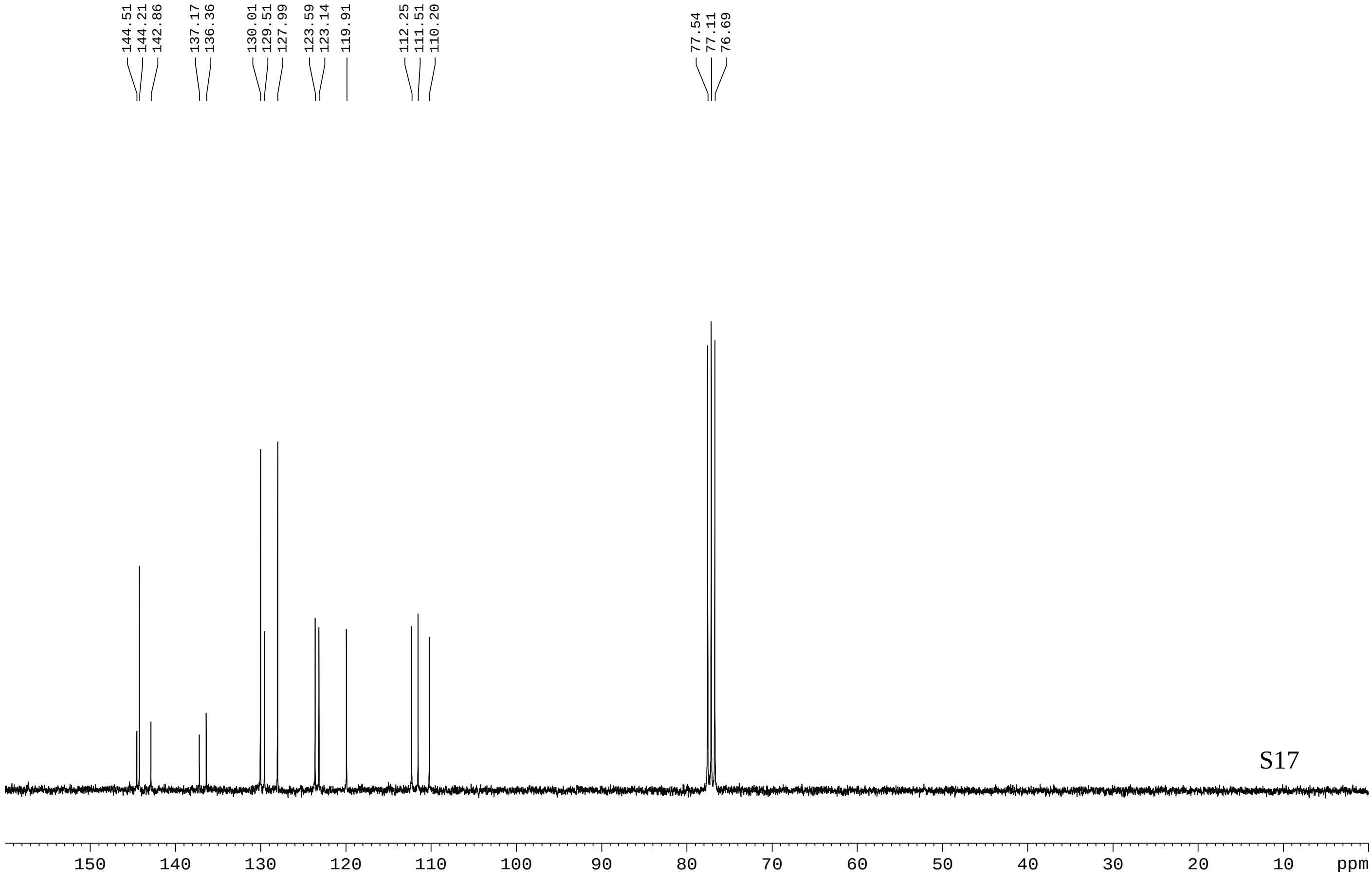

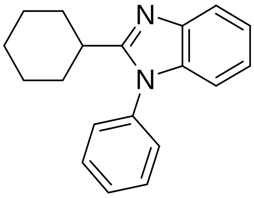

4m

1D proton NMR spectrum

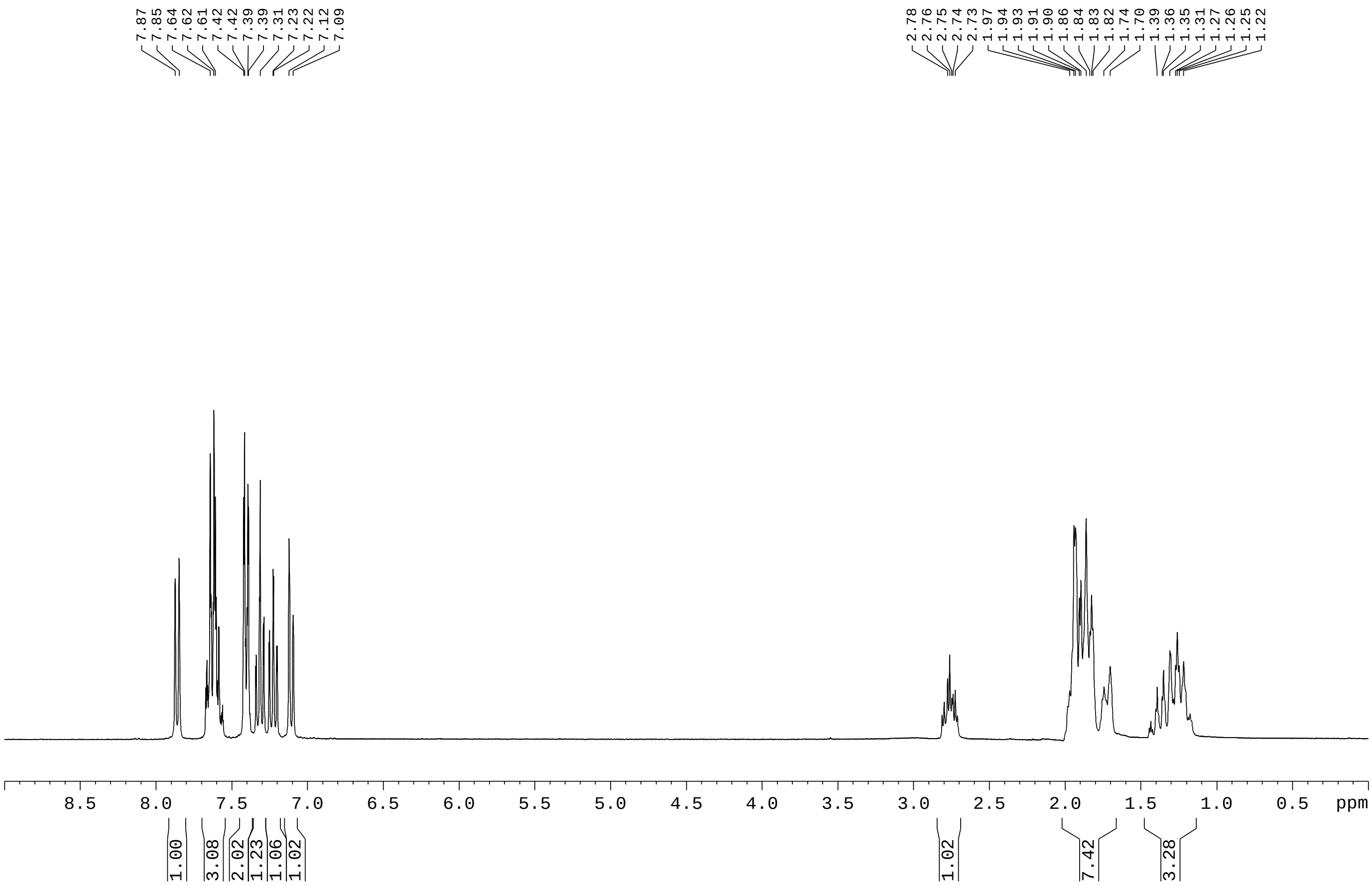

Proton-decoupled carbon NMR spectrum

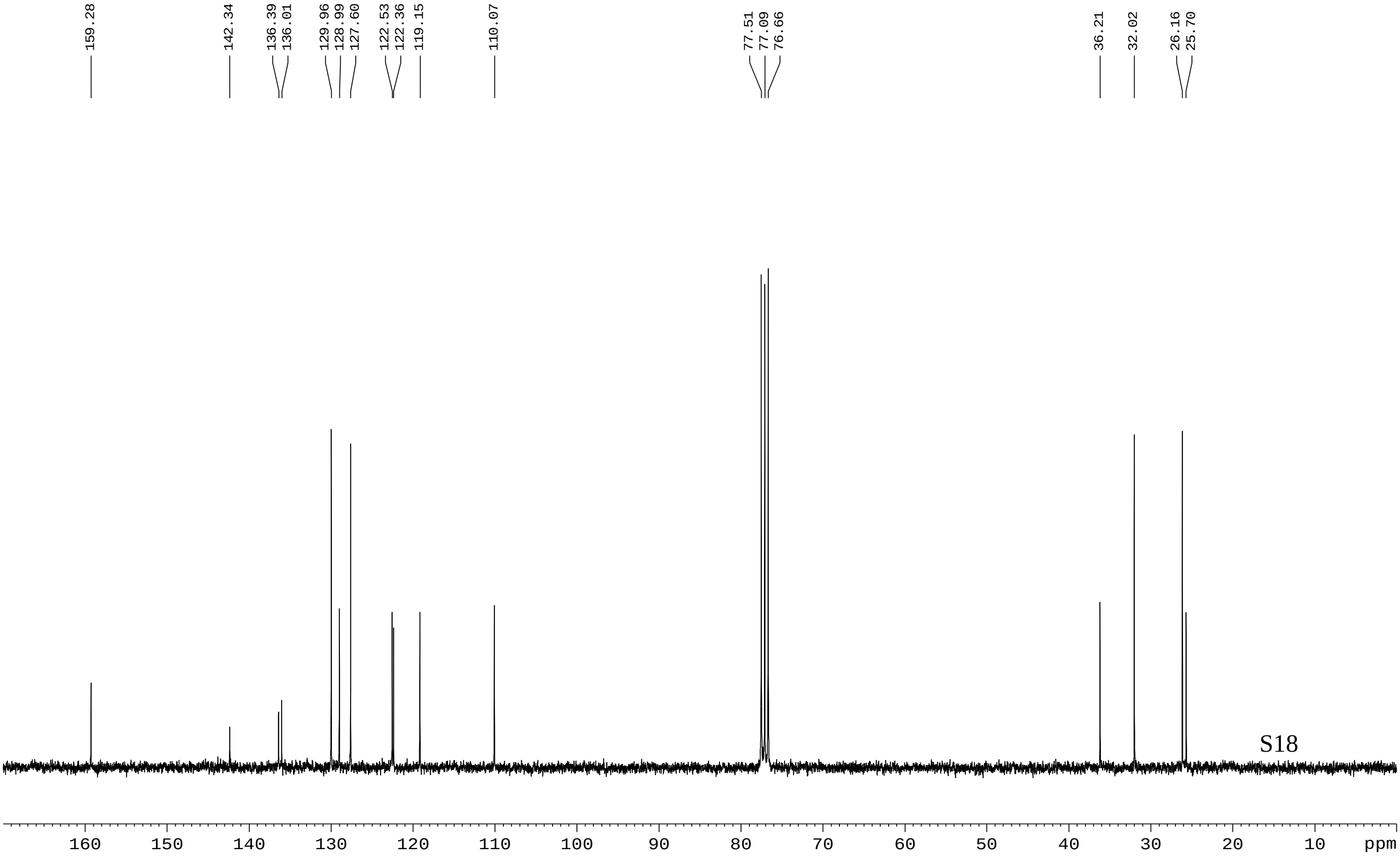

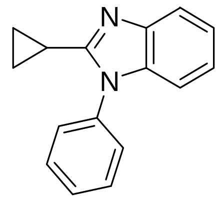

4n

1D proton NMR spectrum

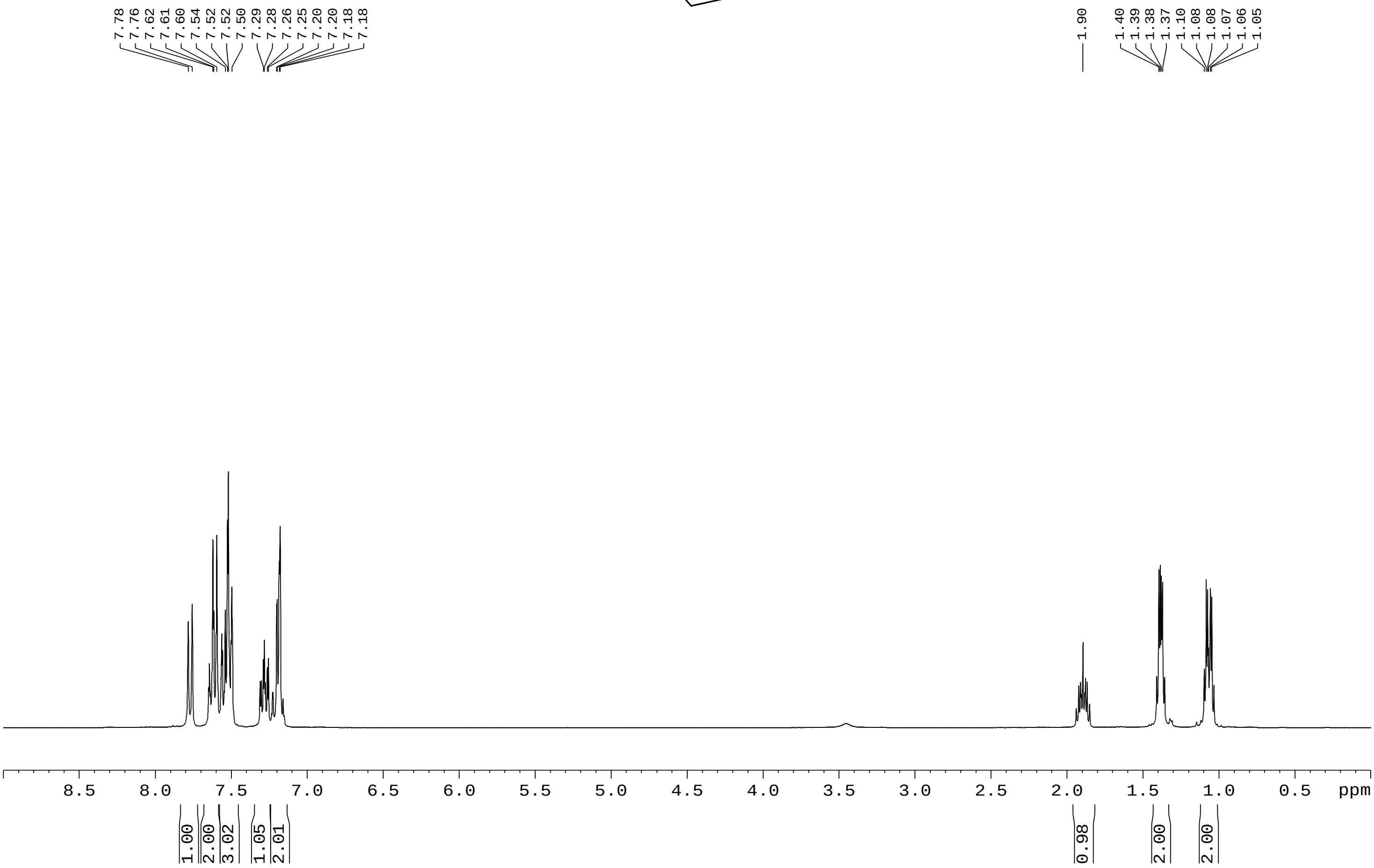

Proton-decoupled carbon NMR spectrum

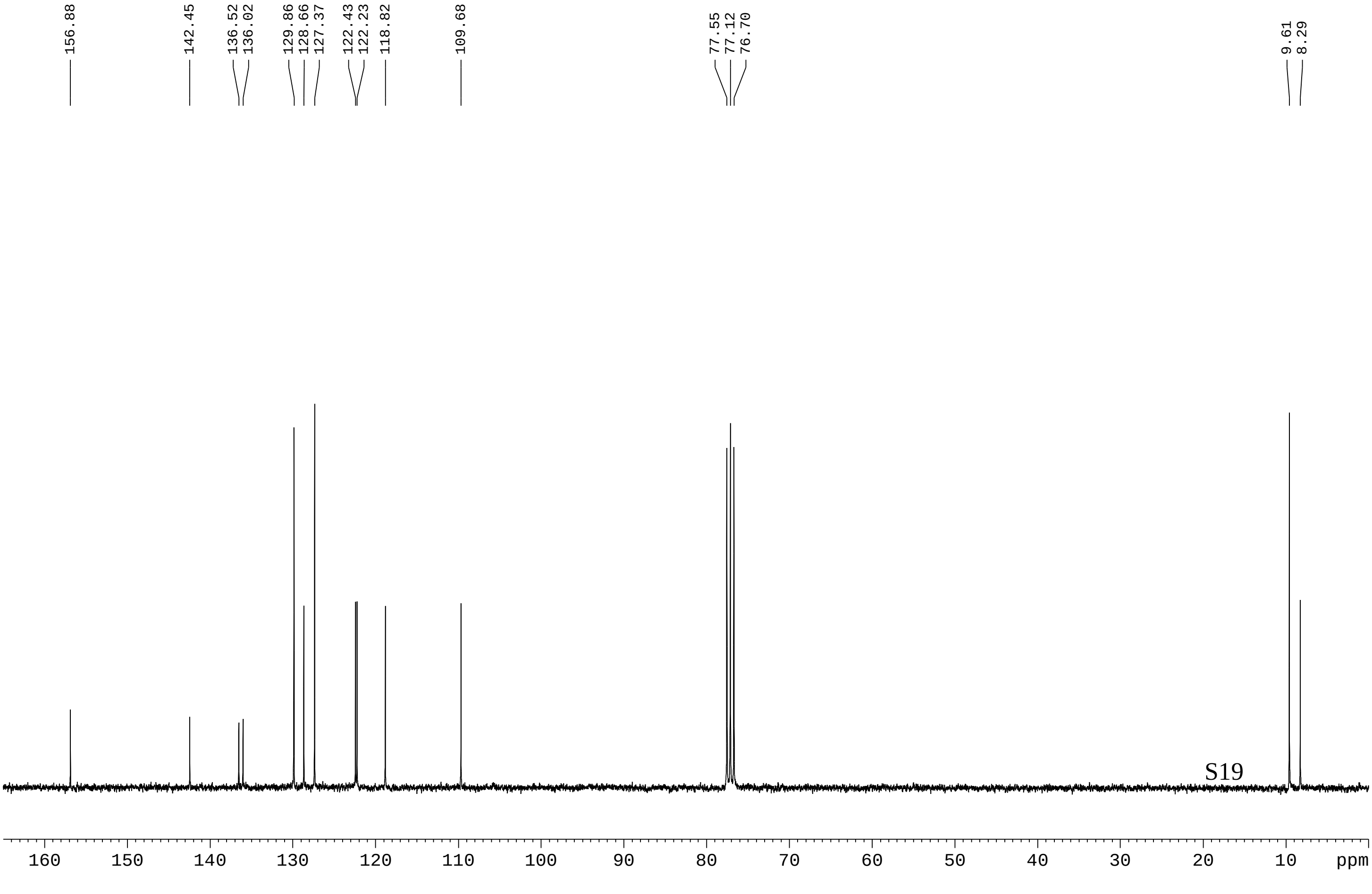

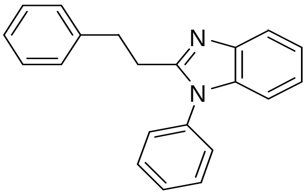

4o

1D proton NMR spectrum

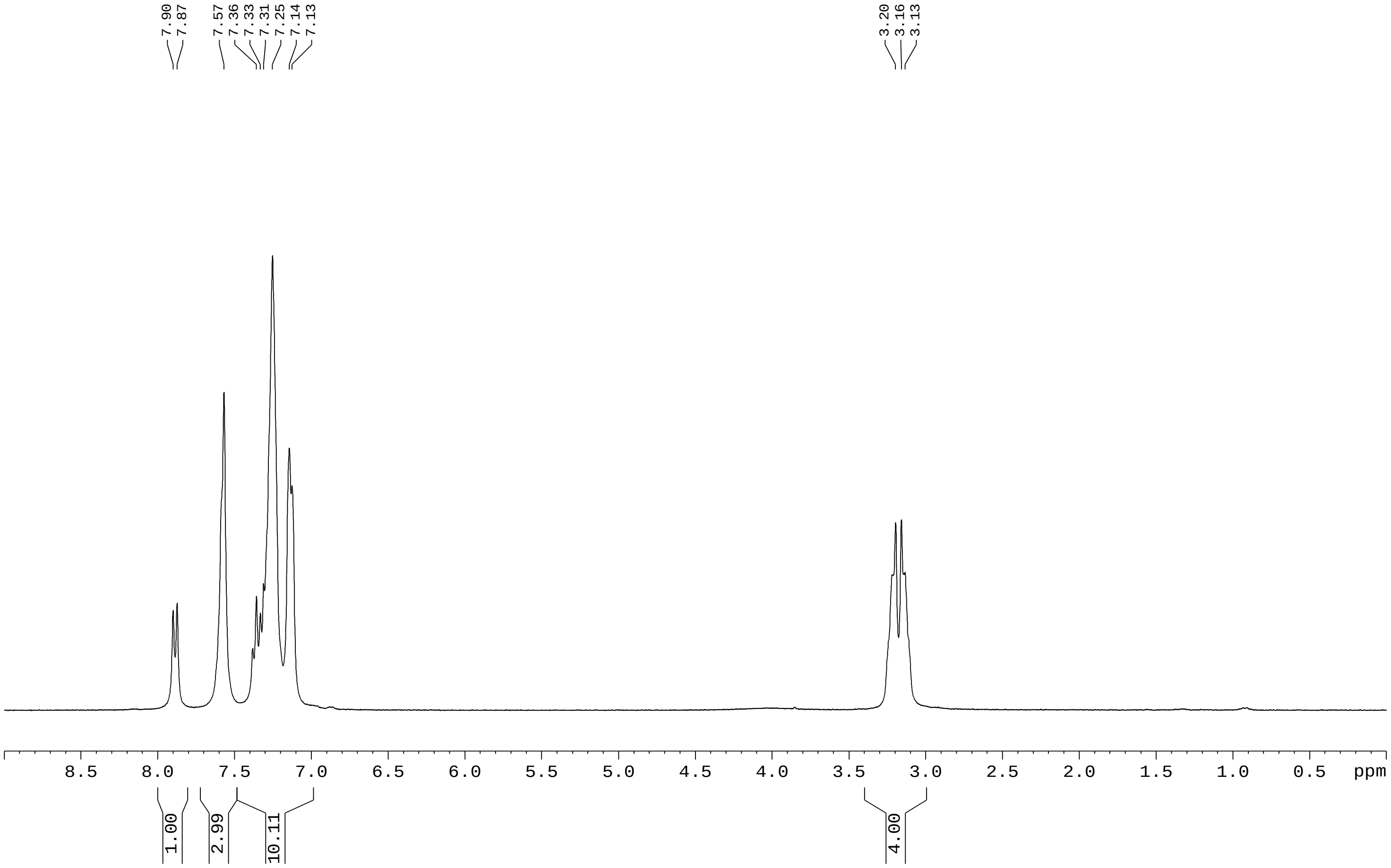

Proton-decoupled carbon NMR spectrum

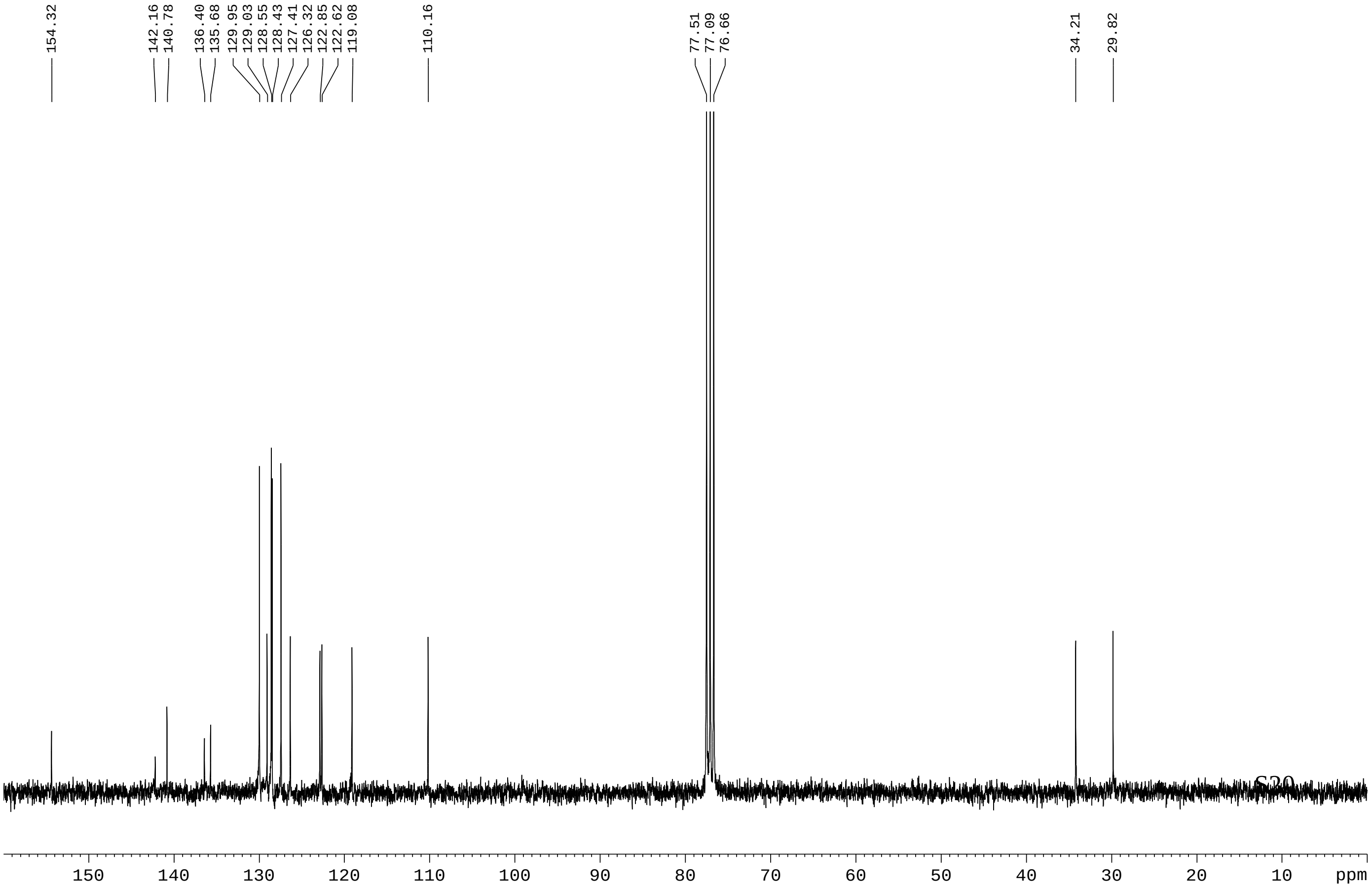

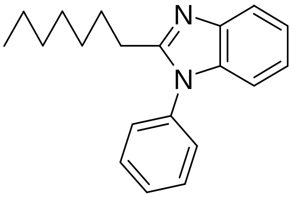

4p

1D proton NMR spectrum

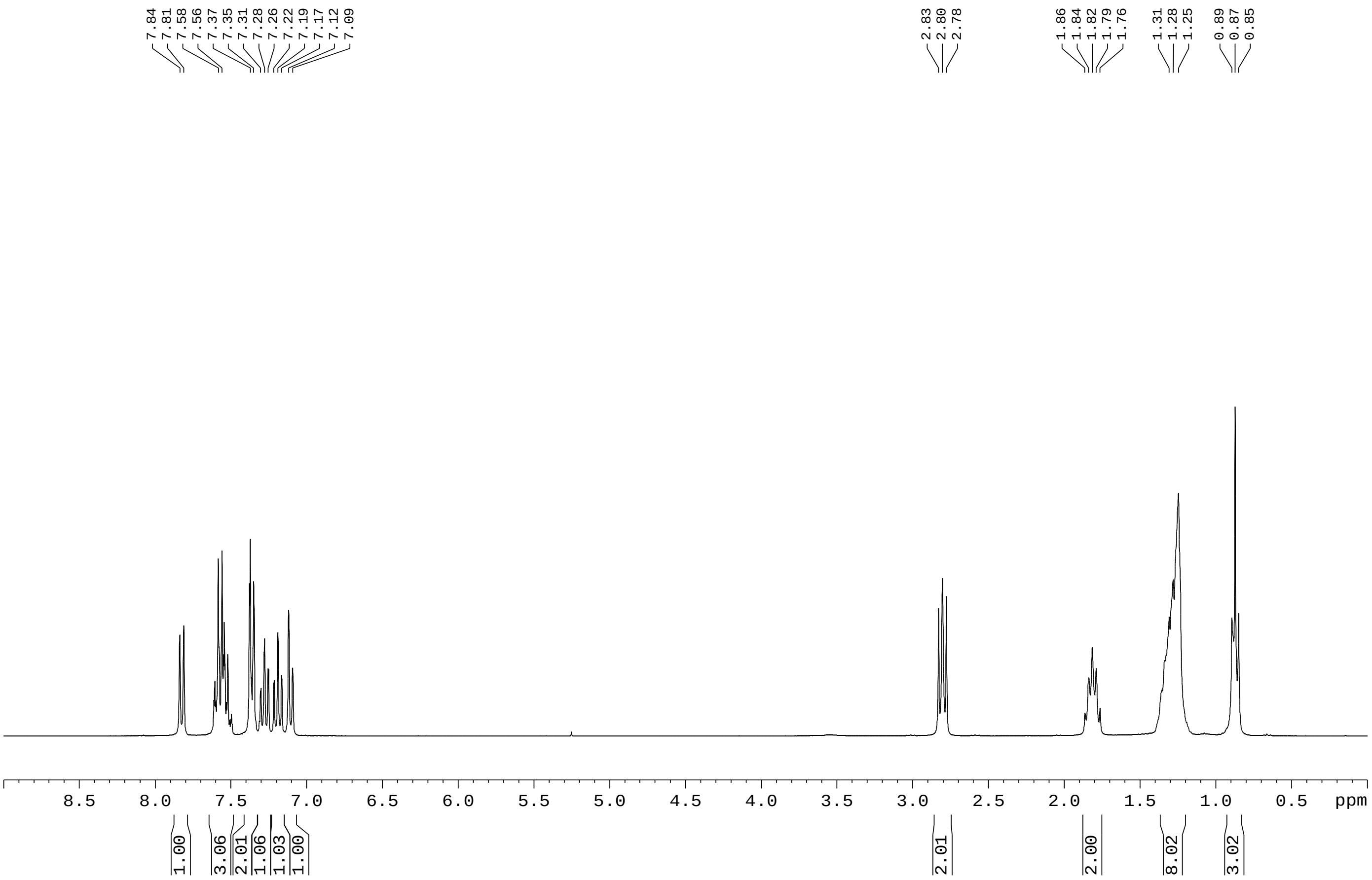

Proton-decoupled carbon NMR spectrum

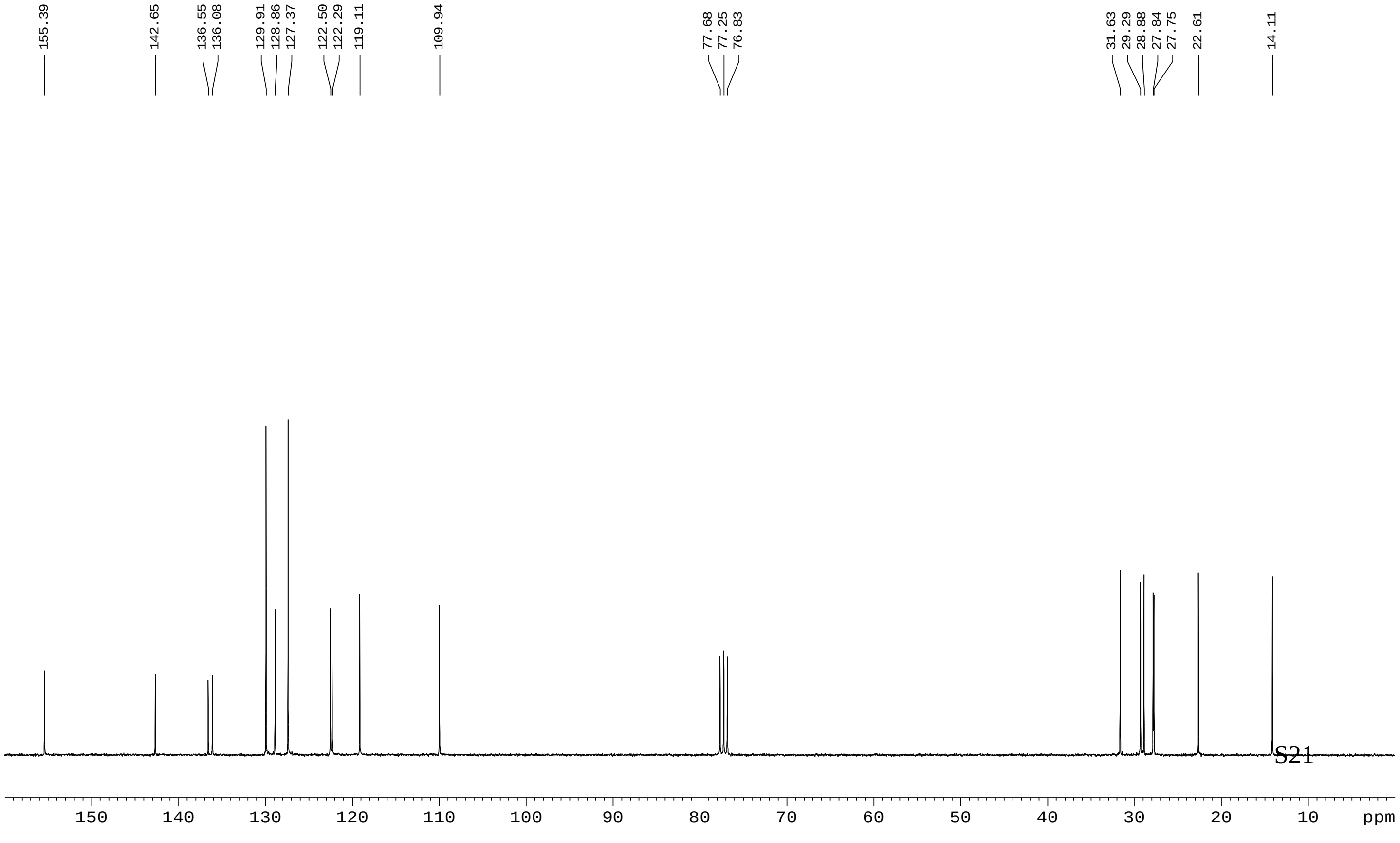

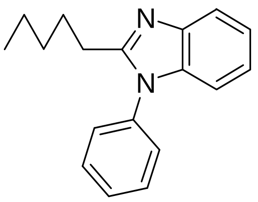

4q

1D proton NMR spectrum

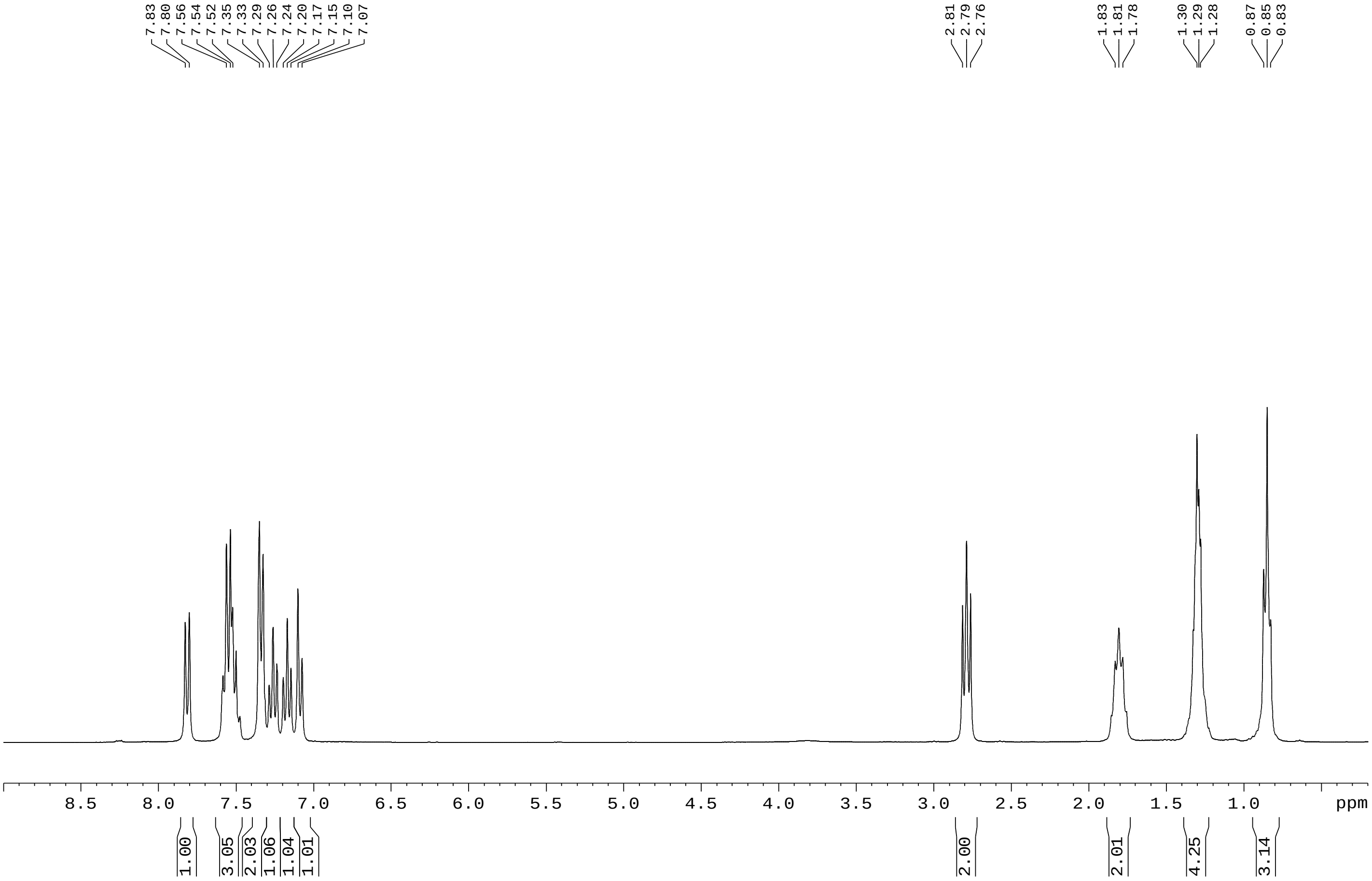

Proton-decoupled carbon NMR spectrum

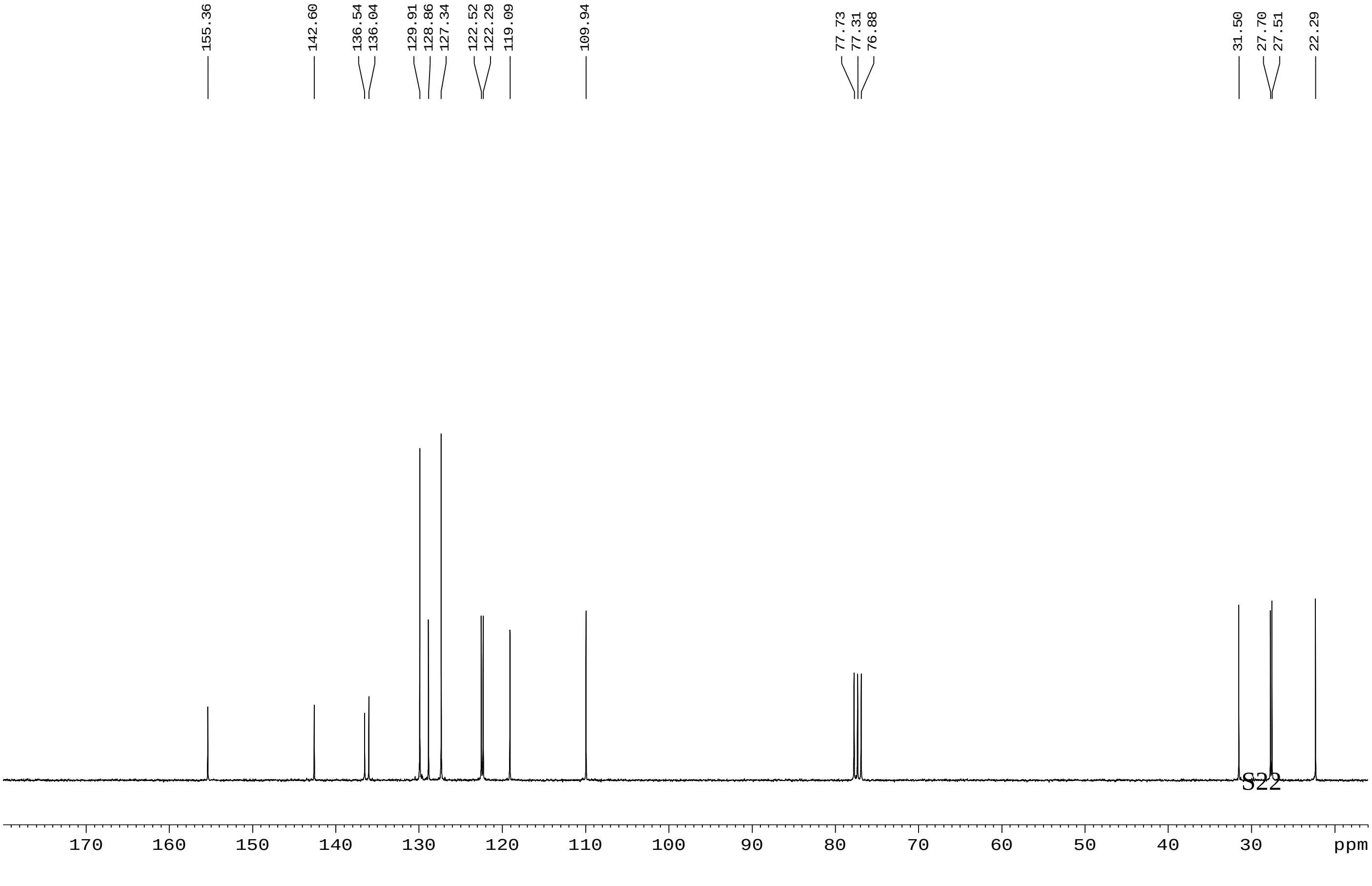

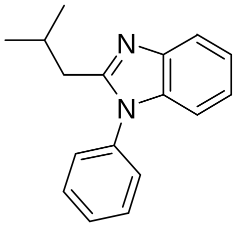

4r

1D proton NMR spectrum

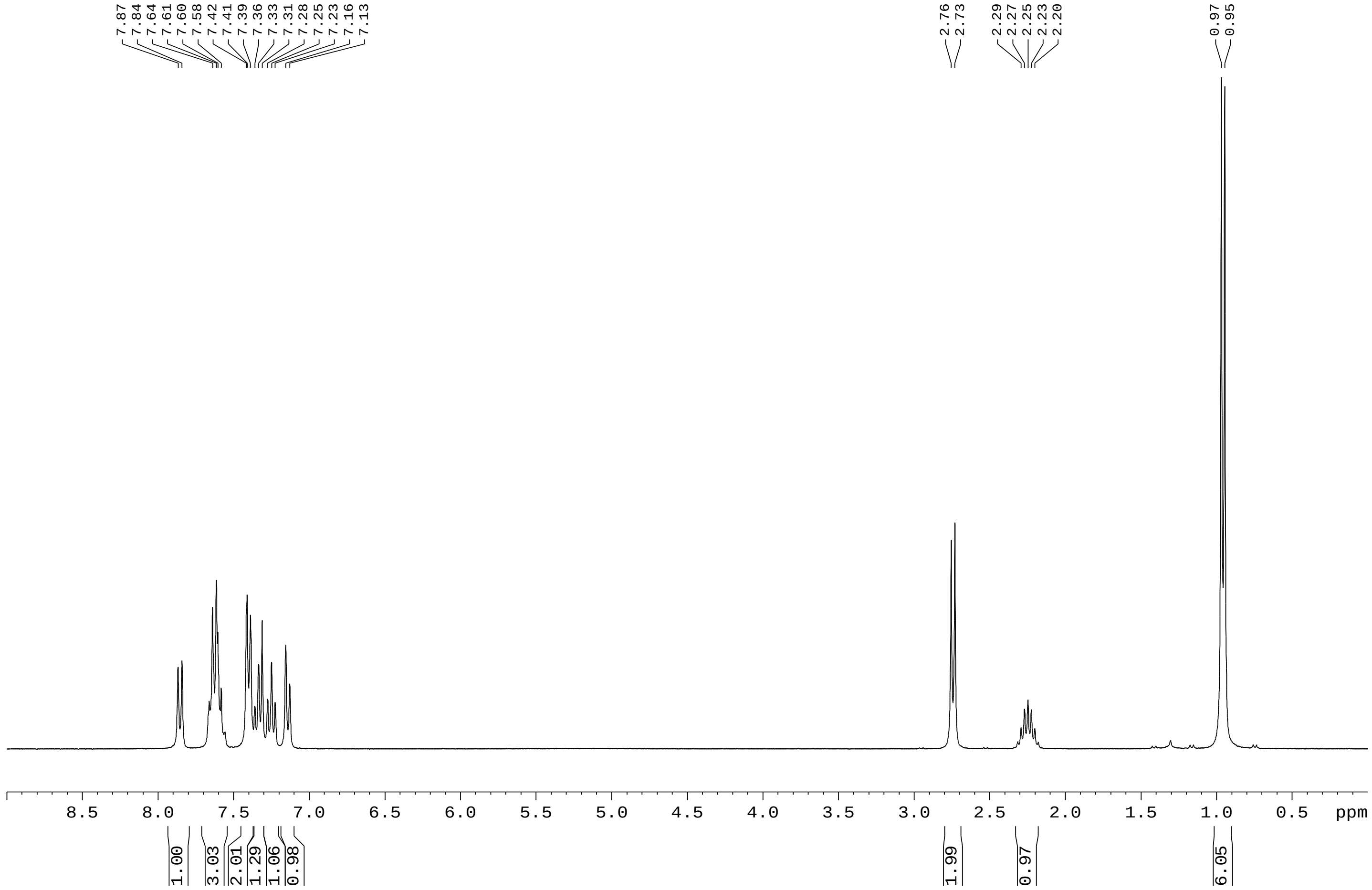

Proton-decoupled carbon NMR spectrum

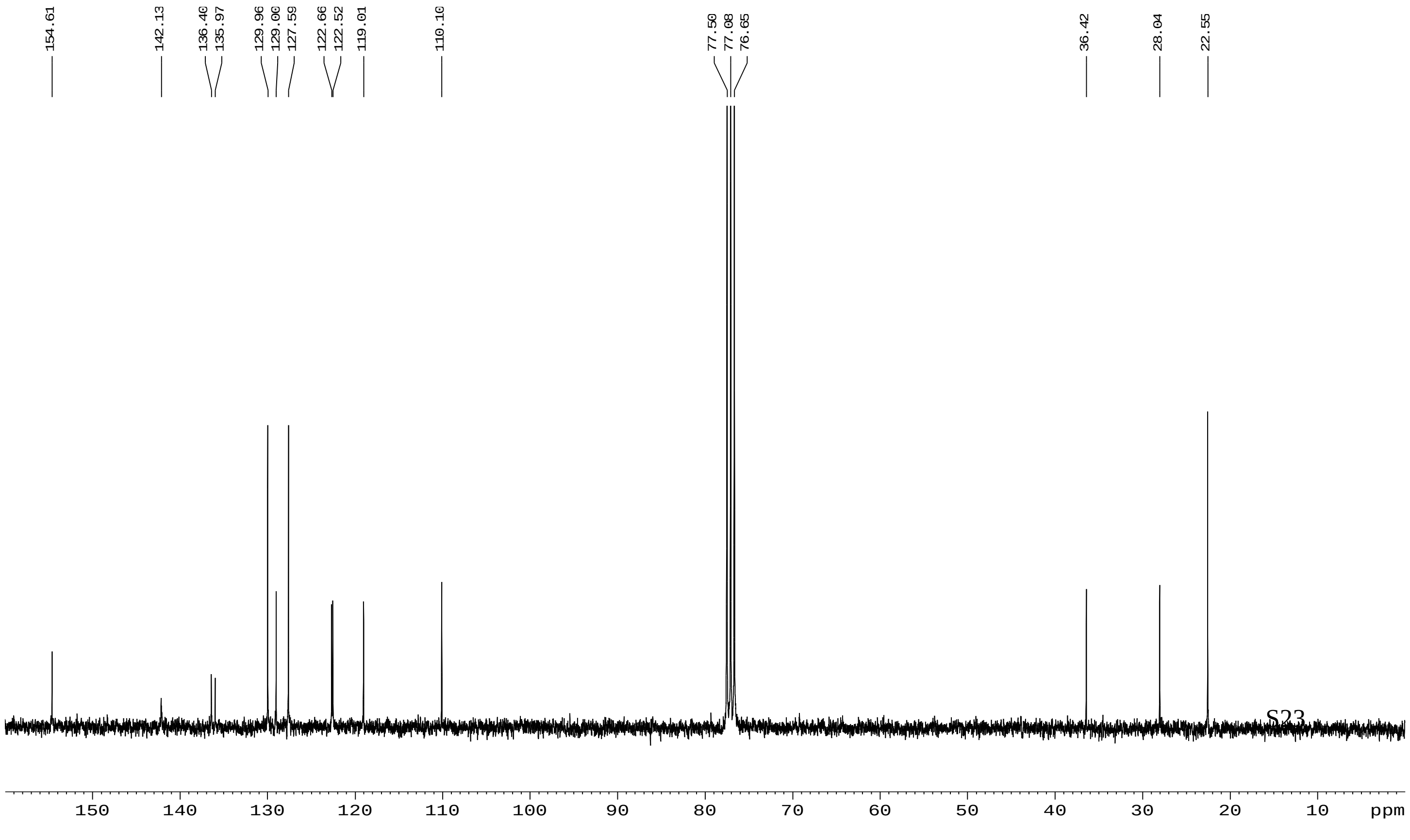

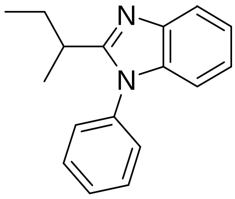

4s

1D proton NMR spectrum

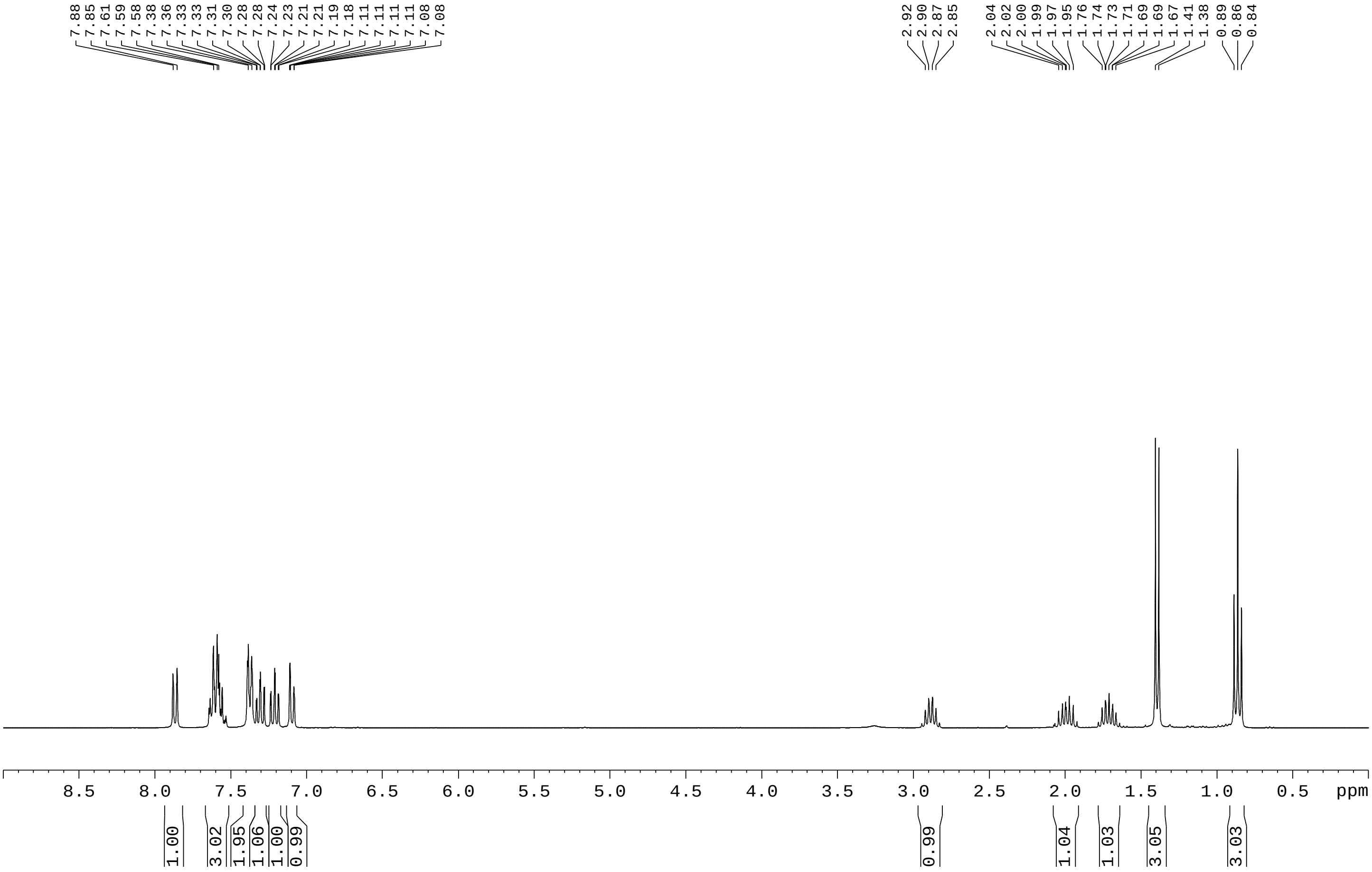

Proton-decoupled carbon NMR spectrum

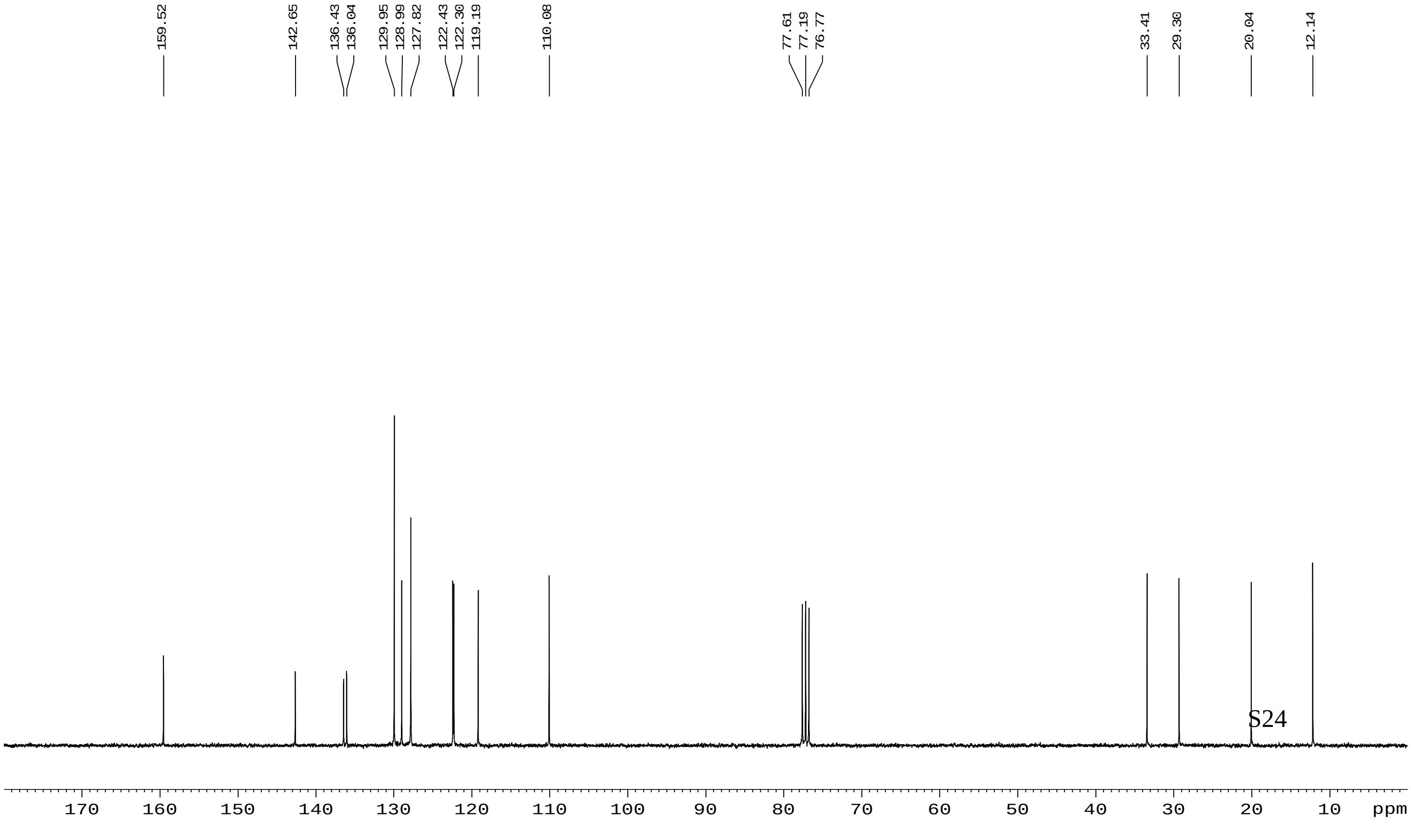

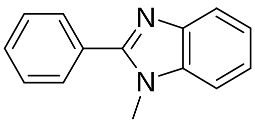

1D proton NMR spectrum

4t

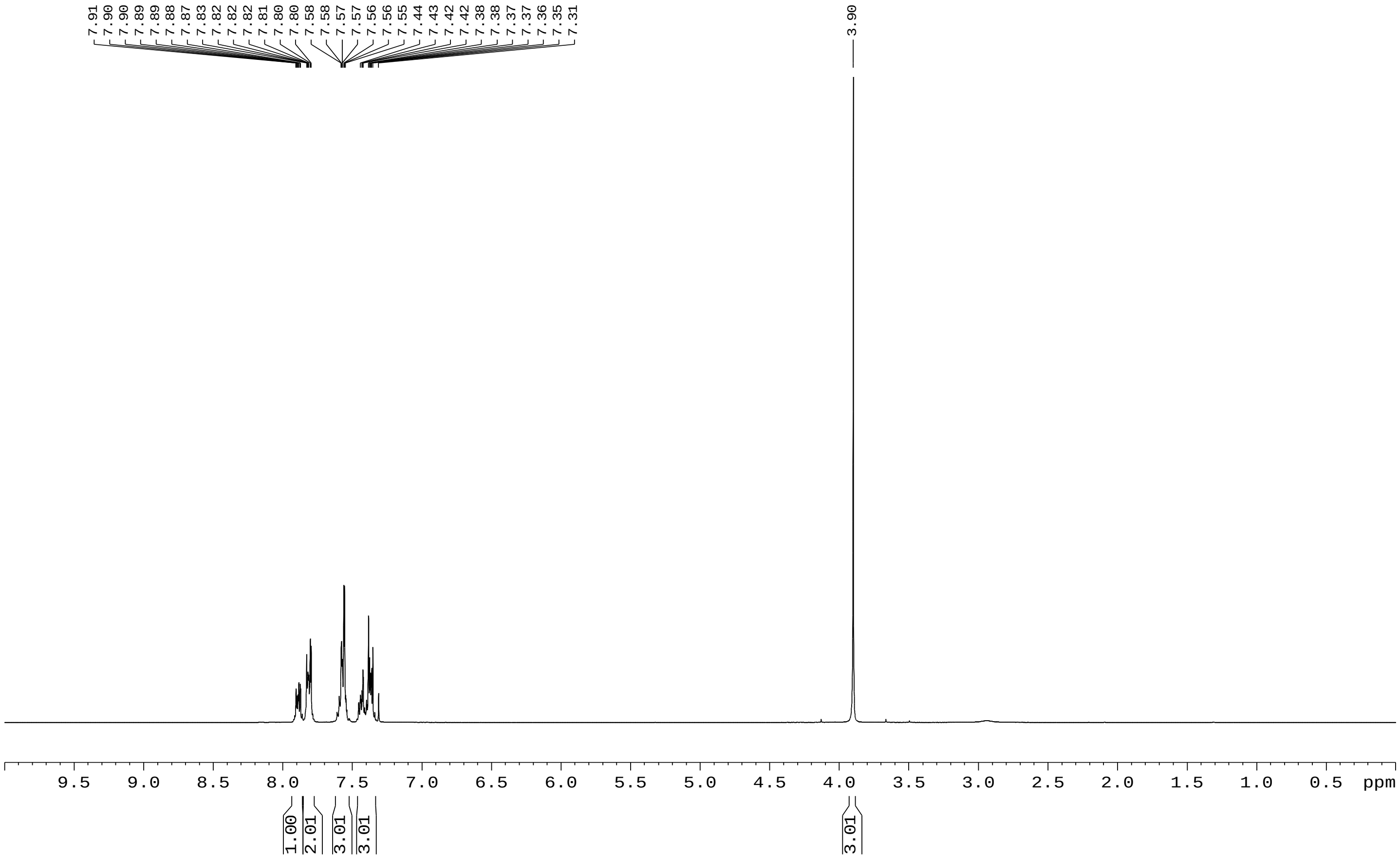

Proton-decoupled carbon NMR spectrum

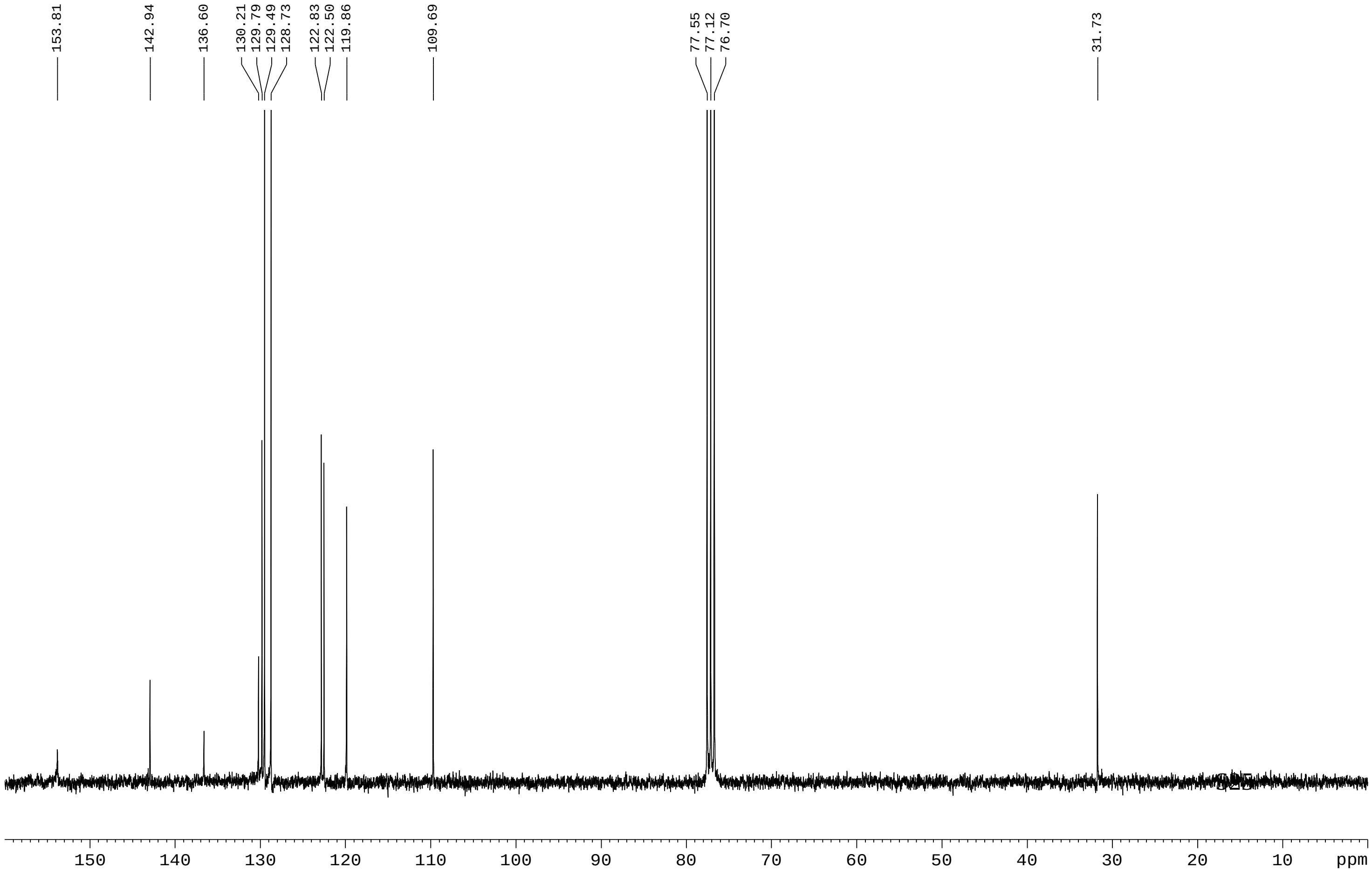

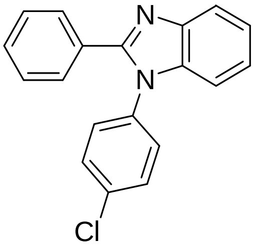

4u

1D proton NMR spectrum

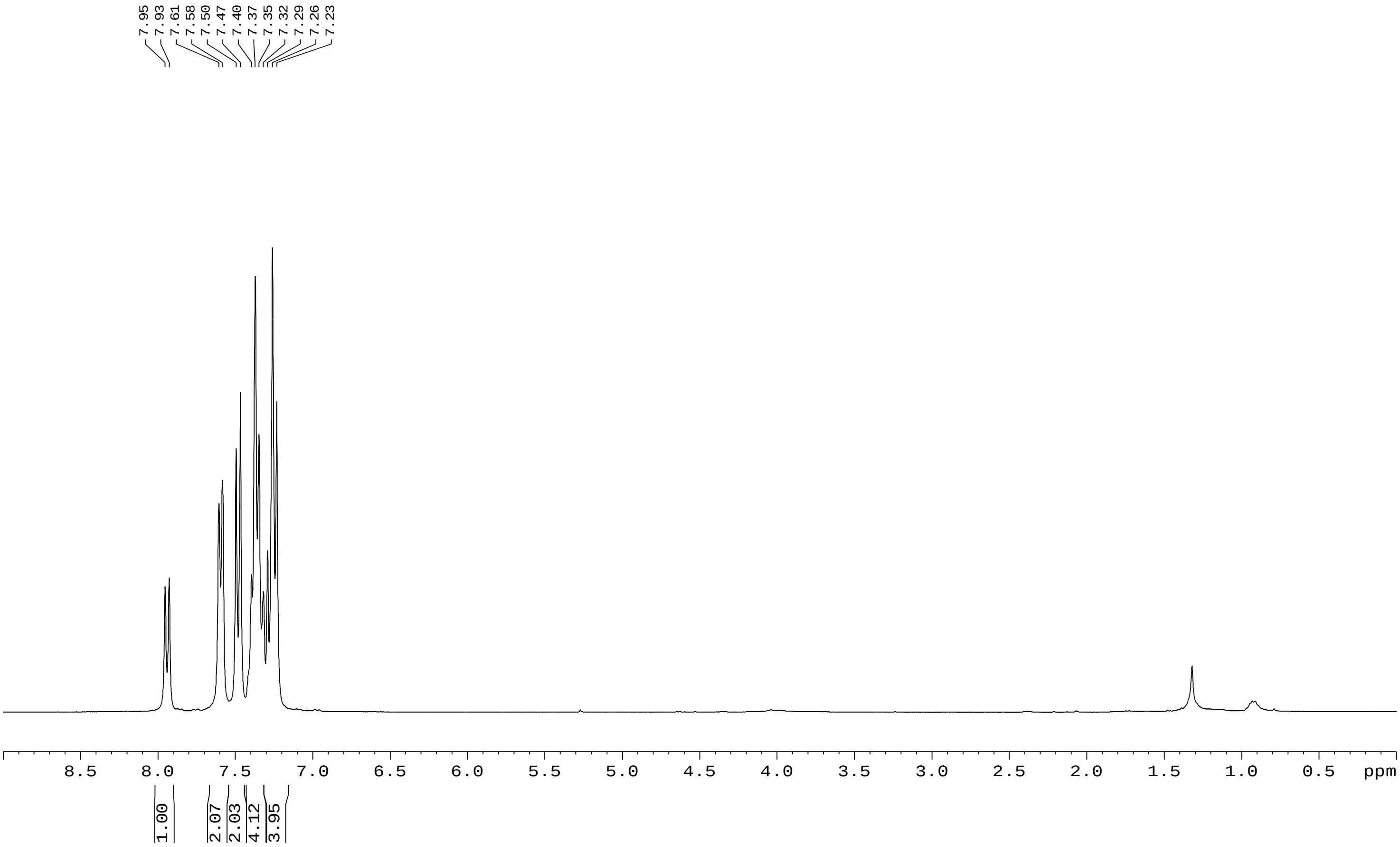

Proton-decoupled carbon NMR spectrum

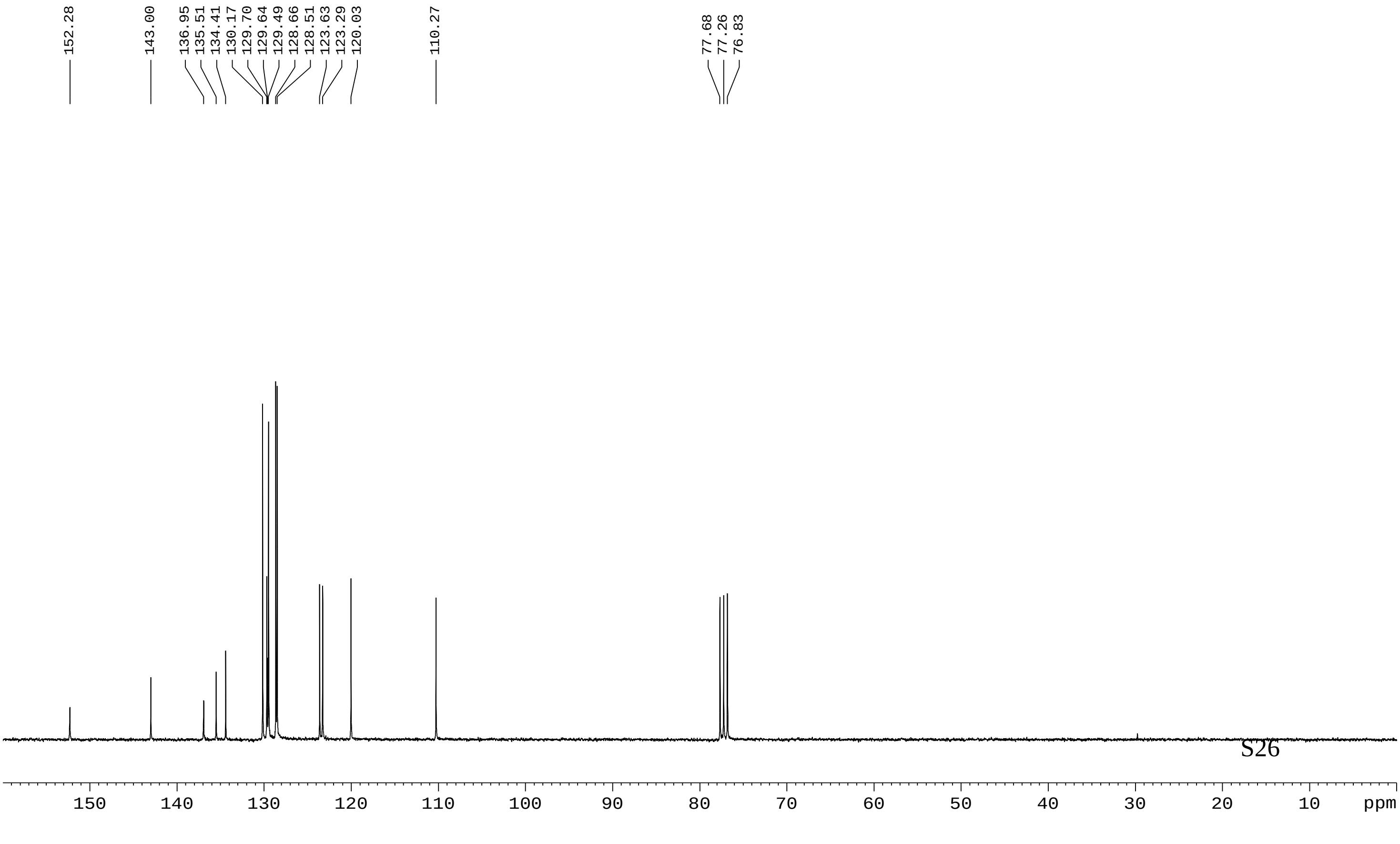

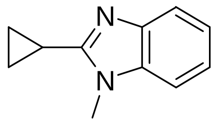

4v

1D proton NMR spectrum

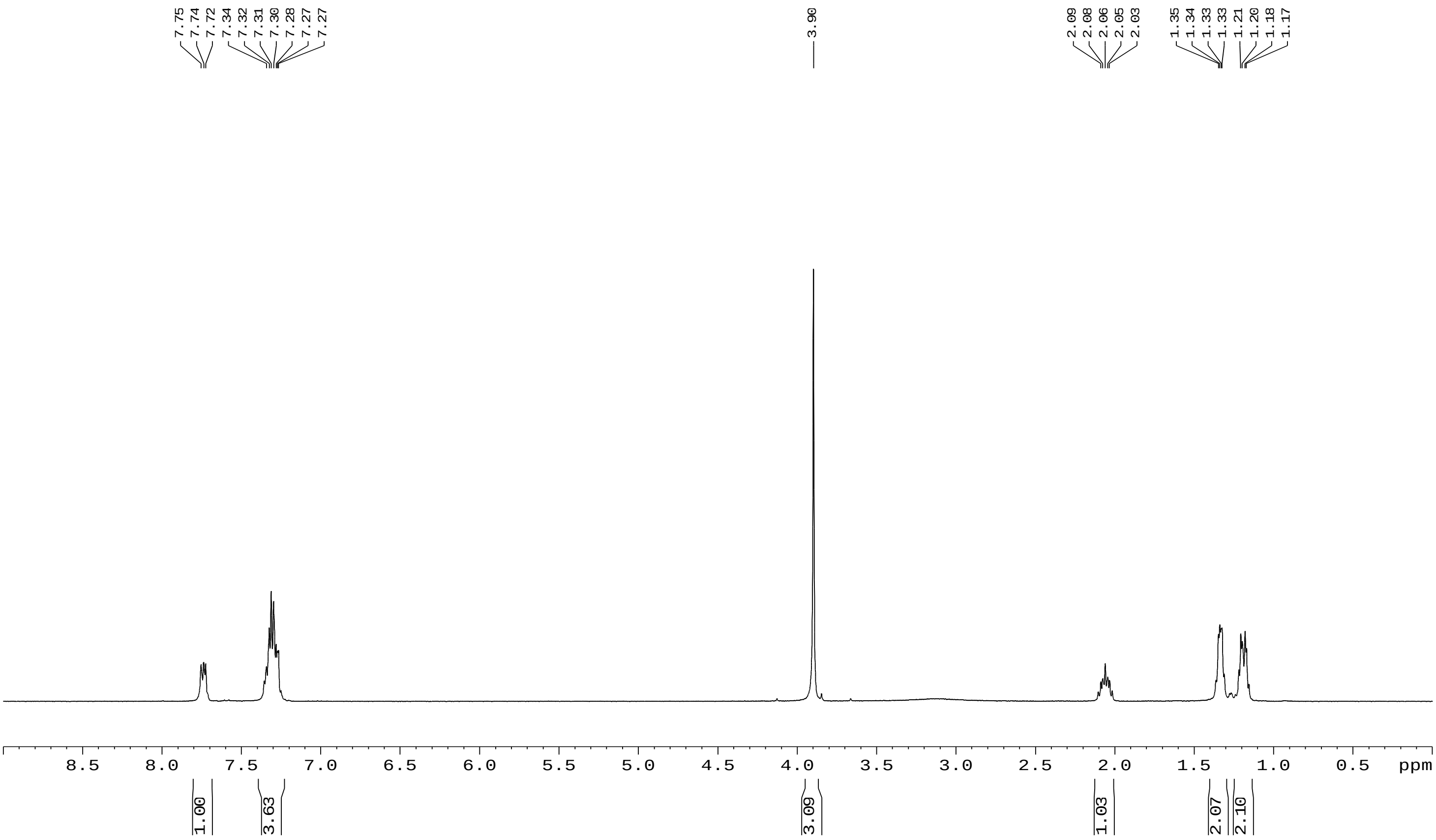

Proton-decoupled carbon NMR spectrum

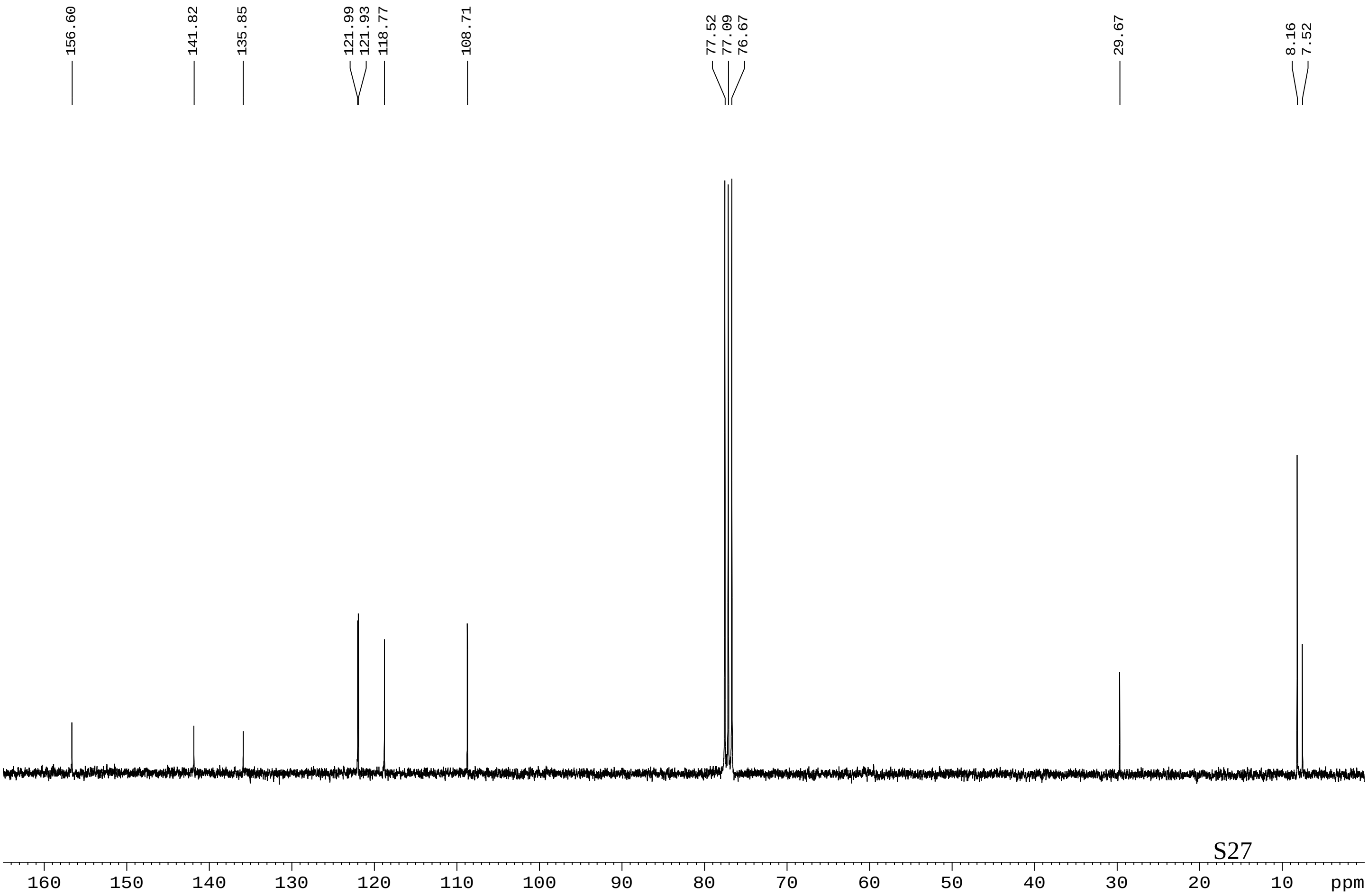

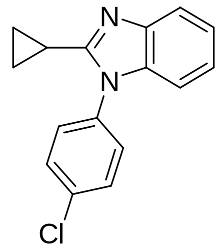

4w

1D proton NMR spectrum

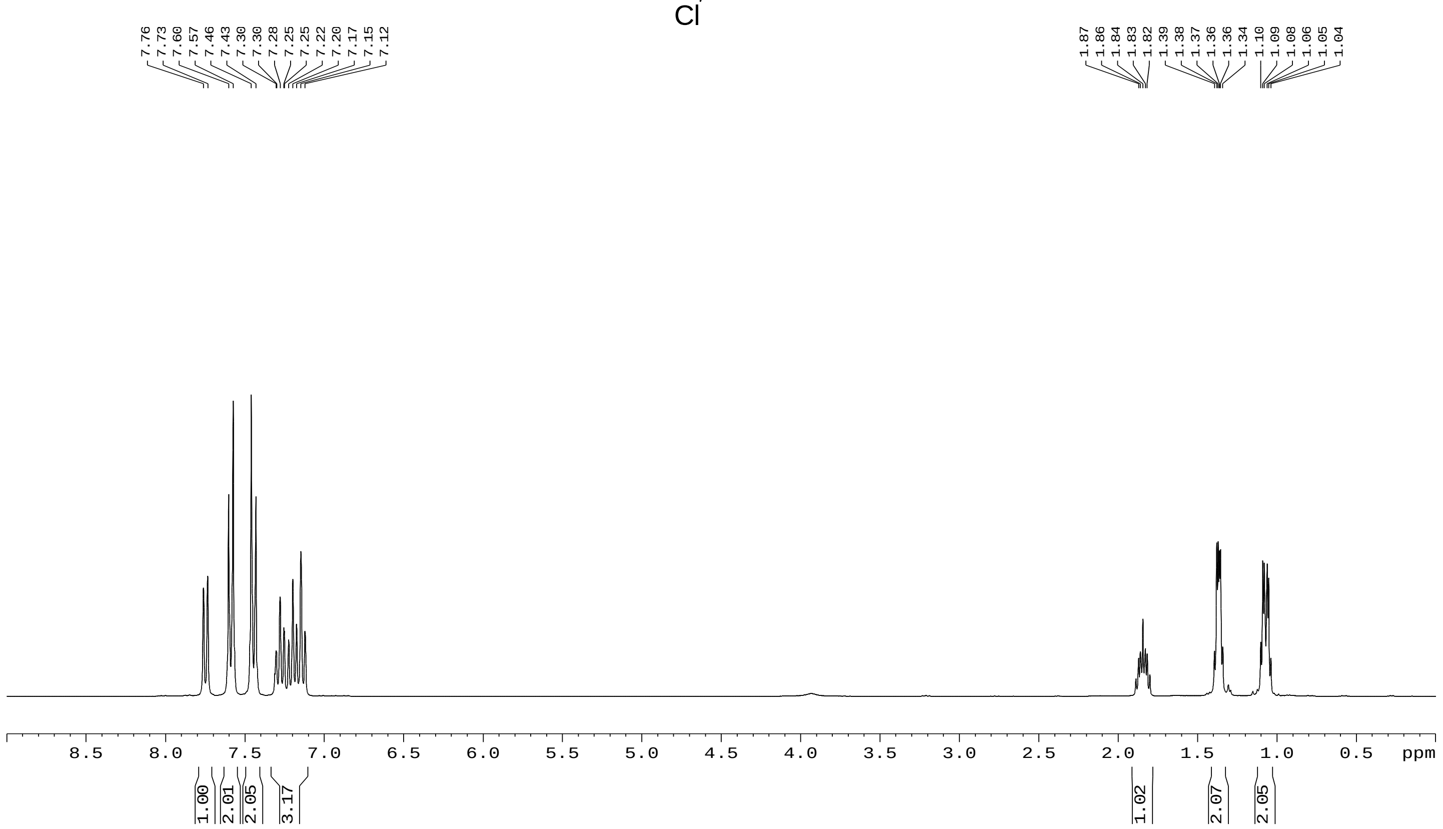

Proton-decoupled carbon NMR spectrum

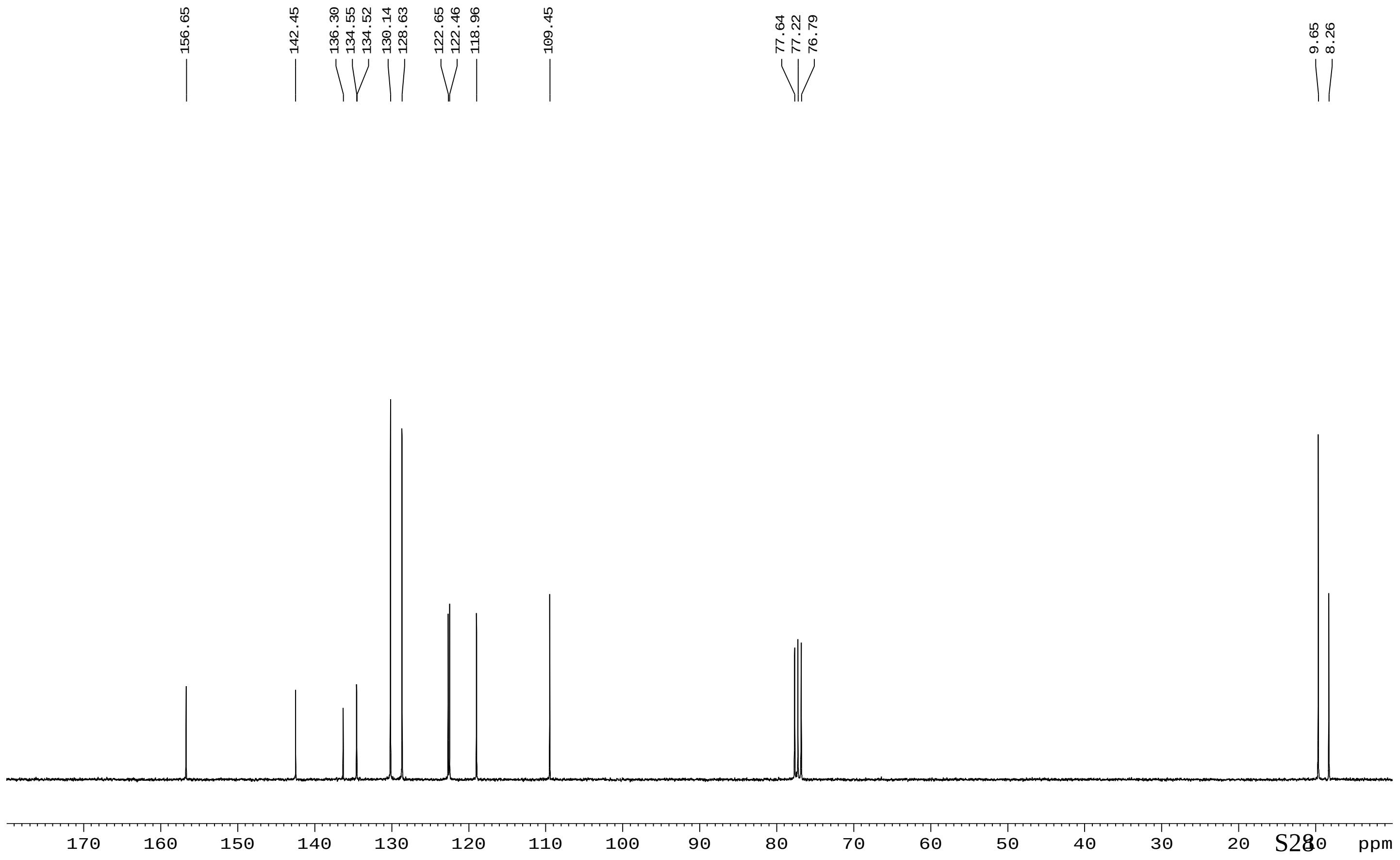

Supplement: Supplementary file 1 — miscellaneous_information [file chem0021-12606-sd1.pdf]
